# Dibucaine induces autophagic flux and reduces Ser129-phosphorylated *α*-synuclein in induced pluripotent stem cell–derived neurons with *SNCA* duplication

**DOI:** 10.3389/fneur.2026.1891393

**Published:** 2026-10-08

**Authors:** Mitsuhiro Kitagawa, Kengo Miyamoto, Kozo Yoshida, Kei-Ichi Ishikawa, Yukiko Sasazawa, Wado Akamatsu, Shinji Saiki, Taku Hatano, Masaya Imoto, Hidetaka Nagata, Nobutaka Hattori

**Affiliations:** 1 Department of Neurology, Juntendo University Faculty of Medicine, Tokyo, Japan; 2 Sumitomo Pharma Co., Ltd., Osaka, Japan; 3 Center for Genomic and Regenerative Medicine, Juntendo University Graduate School of Medicine, Tokyo, Japan; 4 Research Institute for Diseases of Old Age, Juntendo University Graduate School of Medicine, Tokyo, Japan; 5 Division for Development of Autophagy Modulating Drugs, Juntendo University Faculty of Medicine, Tokyo, Japan; 6 Department of Neurology, Institute of Medicine, University of Tsukuba, Ibaraki, Japan; 7 Neurodegenerative Disorders Collaboration Laboratory, RIKEN Center for Brain Science, Saitama, Japan

**Keywords:** alpha-synuclein, autophagy, dibucaine, neurodegenerative disease, Parkinson’s disease, protein aggregation

## Abstract

The accumulation of α-synuclein (α-syn) in the form of Lewy bodies and Lewy neurites is a neuropathological hallmark of Parkinson’s disease (PD) and is closely associated with disease progression. Targeting the autophagy pathway to enhance the clearance of α-syn aggregates therefore represents a promising therapeutic strategy. In the present study, we conducted high-throughput screening using rat embryonic primary neurons, followed by validation of the identified compounds in human induced pluripotent stem cell (iPSC)-derived neurons. We identified dibucaine, a local anesthetic, as a compound that induced autophagic flux in neuronal models. In human iPSC-derived dopaminergic neurons from a patient with PD carrying an *SNCA* duplication, dibucaine reduced cellular levels of Ser129-phosphorylated α-synuclein (pS129 α-syn). In SH-SY5Y cells, dibucaine increased 5’-AMP-activated protein kinase (AMPK) Thr172 phosphorylation. LY364947 partially attenuated dibucaine-induced LC3-II accumulation in the iPSC-derived neurons through an unidentified mechanism. Although the requirement for AMPK and the mechanism underlying the reduction in pS129 α-syn remain unresolved, our findings show that dibucaine induces autophagic flux and reduces pS129 α-syn levels in disease-relevant human neurons.

## Introduction

Parkinson’s disease (PD) is the most prevalent neurodegenerative movement disorder, pathologically characterized by the accumulation of α-synuclein (α-syn) aggregates—known as Lewy bodies—in dopaminergic neurons of the substantia nigra (1, 2). Pathological α-syn propagates in a prion-like manner by seeding endogenous α-syn, driving disease onset and progression (3, 4). Current dopaminergic treatments provide only symptomatic relief and often cause severe side effects, highlighting an urgent unmet medical need for disease-modifying therapies (5). While α-syn can be degraded by both the ubiquitin–proteasome system (UPS) and the autophagy–lysosome pathway (ALP) (6, 7), the UPS only handles small, soluble oligomers. Because autophagic activity declines with age and is distinctly impaired in PD patients, the ALP plays a more critical role under pathological conditions (8, 9). Both chaperone-mediated autophagy (CMA) and macroautophagy are primarily responsible for α-syn clearance (10). However, pathogenic *SNCA* mutations (e.g., A53T, A30P) and post-translationally modified α-syn species disrupt CMA by abnormally interacting with LAMP2A and impairing lysosomal uptake (11–15). Consequently, pharmacological intervention targeting macroautophagy has emerged as a promising therapeutic strategy for PD.

Macroautophagy (hereafter referred to as autophagy) delivers cytoplasmic material to lysosomes through the formation and subsequent lysosomal fusion of double-membrane autophagosomes (16). Autophagy is regulated through both mammalian target of rapamycin (mTOR)-dependent and mTOR-independent pathways. The mTOR-dependent pathway integrates signals related to nutrient availability, growth factors, cellular energy status, and stress, whereas several mTOR-independent pathways respond to diverse intracellular signals and pharmacological stimuli (17). In the mTOR-dependent pathway, mTOR complex 1 (mTORC1) negatively regulates autophagy by phosphorylating unc-51 like autophagy activating kinase 1 (ULK1), which is essential for autophagosome formation (18), and transcription factor EB, which is a master regulator of autophagy and lysosomal gene expression (19). Nutrient deprivation suppresses mTORC1 signaling and thereby facilitates autophagy induction (16). 5’-AMP-activated protein kinase (AMPK) can promote autophagy through direct regulation of ULK1 and through indirect modulation of mTORC1 signaling. Additional signaling pathways involving inositol, Ca^2+^/calpain, cAMP, and Beclin-1 can also regulate autophagy (20, 21). Because these pathways interact at multiple levels, autophagy induction may be accompanied by complete, partial, or no readily detectable suppression of canonical mTORC1-associated signaling, depending on the cellular and experimental context.

In the present study, we screened autophagy-modulating small molecules using several chemical libraries in rat embryonic primary neurons and subsequently evaluated the identified compounds in human induced pluripotent stem cell (iPSC)-derived neurons. We identified dibucaine, also known as cinchocaine, as a compound that induces autophagic flux in neuronal models. We further found that dibucaine treatment reduced the cellular level of pS129 α-syn in iPSC-derived neurons from a patient with PD carrying an *SNCA* duplication. We also examined AMPK- and mTOR-associated signaling and the effects of LY364947 to explore pathways potentially associated with the cellular response to dibucaine.

## Materials and methods

### Animals

All animal procedures were approved by the Institutional Animal Experiment Ethics Committee of Sumitomo Pharma and were conducted in accordance with the Sumitomo Pharma guidelines for the care and use of laboratory animals. All efforts were made to minimize animal suffering and the number of animals used. A total of 27 pregnant Crlj:WI (Wistar) rats were purchased from Jackson Laboratory Japan (Tsukuba, Japan). Primary neurons were prepared from embryos at embryonic day 18 (E18). Embryos from each litter were processed separately for each culture preparation. Embryonic sex was not determined. Pregnant rats were euthanized by CO₂ inhalation before embryo collection. The animals were used exclusively to obtain embryonic tissues, and no *in vivo* treatment or experimental group allocation was performed; therefore, randomization and blinding at the animal level were not applicable.

### Compound library

We constructed a screen of 1,637 bioactive compounds with annotated target information. For the breakdown of the libraries indexed for this screening, a kinase inhibitor library comprising 390 compounds and an autophagy-related library comprising 421 compounds were purchased from Selleck Biotechnology (Yokohama, Japan) and MedChemExpress (Monmouth Junction, NJ, United States), respectively. Additionally, a US Food and Drug Administration (FDA)-approved compound library, comprising 754 compounds, was purchased from the Prestwich Chemical Library (Prestwick Chemical Inc., Illkirch-Graffenstaden, France), along with a library of 72 compounds exhibiting high brain transferability.

### Viral vector production

Viral vectors were packaged in three plasmids. Each plasmid expressed the adeno-associated virus (AAV) 2 replicase, AAV-Dj capsid protein (pAAV-Dj; CELL BIOLABS Inc., San Diego, CA, United States) and adenovirus helper proteins (pHelper; Agilent, Santa Clara, CA, United States). The plasmids were co-transfected into AAV-293 cells (Agilent) at a molar ratio of 1:1:1. In a transfer plasmid, enhanced green fluorescent protein (EGFP)-LC3B-mCherry (22) was inserted between the human synapsin (hSyn) I promoter and bGH polyA. This recombinant AAV (rAAV) vector was prepared using the adenovirus-free triple-plasmid transfection system and the purification method previously reported by Matsushita et al. (23). Viral vectors were stored at −80 °C, and the genomic titer of each virus was determined using quantitative polymerase chain reaction.

### High-throughput screening in neuronal cells

Dissociated cortical cultures from embryonic day 19 Crlj:WI (Wistar) rats were prepared as described previously.^1^ Briefly, after papain enzyme treatment (Sigma-Aldrich, St. Louis, MO, United States), the dissociated neurons were plated onto poly-D-lysine-coated 384-well plates (Corning Incorporated, Corning, NY, United States; 354663) at a density of 1 × 10^4^ cells/well. After 7 days *in vitro*, the neurons were infected with AAVdj-hSynI-EGFP-LC3B-mCherry at a multiplicity of infection of 1 × 10^5^ viral genomes per cell. After 11 days *in vitro*, each compound was dissolved in the medium, and the solution was added to each well of the assay plate at a final concentration of 10 μM using an EDR-384SR (BioTec Co., Ltd., Tokyo, Japan). The cells were then incubated for 24 h. To analyze autophagic flux, the cells were fixed with 4% paraformaldehyde, and nuclear staining was performed using Hoechst (Dojindo, Kumamoto, Japan, Cat # H342, 1:1000). Following the previously reported method (24), image acquisition was performed using an IN Cell Analyzer 6,000 (Cytiva, Marlborough, MA, United States) at 20 × magnification in the nonconfocal mode with Hoechst 33342, fluorescein isothiocyanate (FITC) and Texas Red channel set. Nine images per well were collected. Data analysis was performed using IN Cell Investigator Developer Toolbox (1.9.2) software (24).

### Human iPSC culture

The human *SNCA* duplicated iPSC line DupA13 used in this study was established in a previous study (25) under a protocol approved by the Juntendo University School of Medicine Ethics Committee (approval no. M08-0449), with written informed consent from the donor. No new human participants were recruited, and no new samples were collected for the present study. And all experiments involving human iPSCs were conducted in compliance with relevant guidelines and regulations. The iPSCs were cultured in feeder-free conditions in StemFit AK02N (Reprocell, Yokohama, Japan) medium on iMatrix-511 (Takara Bio, Shiga, Japan)-coated plates and passaged every 7 days as reported (26).

### *In vitro* differentiation into midbrain dopaminergic neurons

The differentiation of Parkinson’s disease 4 (PARK4) DupA13 cells into dopaminergic neurons was achieved via neurospheres, as described previously (26, 27), to maintain relatively high reproducibility of efficient differentiation for this cell line. We passaged DupA13 neurospheres twice to prepare a large amount of progenitor cells, which were then seeded onto poly-L-ornithine/fibronectin-coated plates. Next, the cells were differentiated into dopaminergic neurons in neuronal medium (KBM Neural Stem Cell Medium with 1xB27 Plus [Thermo Fisher Scientific, Waltham, MA, United States] and Culture One [Gibco, Waltham, MA, United States]) supplemented with 20 ng/mL brain-derived neurotrophic factor (BioLegend, San Diego, CA, United States), glial cell-derived neurotrophic factor (PeproTech Inc., Cranbury, NJ, United States), 200 μM ascorbic acid (Sigma-Aldrich), 0.5 mM dibutyryl-cyclic adenosine monophosphate (Nacalai Tesque, Kyoto, Japan), 1 ng/mL transforming growth factor β3 (BioLegend), and 10 μM of the Notch signaling inhibitor DAPT (Sigma-Aldrich), under a humidified atmosphere with 5% CO_2_ and 4% O_2_. For the first 3 days, 3 μM of CHIR99021 (Reprocell) was in the medium, and every 2 days, half of the medium was changed to medium without CHIR99021. After more than 10 days of incubation, neurons developed and were used for the experiments.

### Cells and reagents

Human neuroblastoma SH-SY5Y cells were purchased from the American Type Culture Collection (ATCC, Manassas, VA, United States). Dibucaine (Cat# 049-20011) and tetracaine (Cat# 207-09031) were obtained from Fujifilm Wako Pure Chemical Corporation (Osaka, Japan). Lidocaine was purchased from Sigma-Aldrich (Cat# L5647), and proparacaine was obtained from Tokyo Chemical Industry (TCI, Tokyo, Japan; Cat# P2156). LY364947 was purchased from TargetMol (Boston, MA, United States; Cat# T2048), and SB431542 was from TCI (Cat# B4003). Other general chemicals and reagents were obtained from commercial sources and were of analytical grade unless otherwise specified.

### Western blot analysis

Protein extracts were electrophoresed and incubated with corresponding specific antibodies against LC3B (Medical & Biological Laboratories Co., Ltd., Tokyo, Japan, Cat # PM036, 1:1000; Sigma-Aldrich, Cat # L7543, 1:5000), glyceraldehyde 3-phosphate dehydrogenase (GAPDH; Merck Millipore, Darmstadt, Germany, Cat # CB1001, 1:1000; Cell Signaling Technology [CST], Tokyo, Japan, Cat # 2118, 1:2000), phosphorylated AMPKα (Proteintech, Tokyo, Japan, Cat # 80209-6-RR, 1:2000), AMPKα (CST, Cat # 5831, 1:1000), mTOR (CST, Japan, Cat#2929, 1:1000), p-mTOR (S2448) (CST, Japan, Cat#2971, 1:1000), ULK1 (CST, Japan, Cat#8054, 1:1000), p-ULK1 (S757) (CST, Japan, Cat#6888, 1:1000), P70 S6 Kinase (CST, Japan, #2708, 1:1000), p-P70 S6 Kinase (T389) (CST, Japan, Cat#9234, 1:1000), S6 Ribosomal Protein (CST, Japan, Cat#2217, 1:1000), p-S6 Ribosomal Protein (S235/236) (CST, Japan, Cat#4858, 1:1000), α-tubulin (Proteintech, Cat # 66031-1-Ig, 1:5000), phosphorylated α-syn (CST, Cat # 23706, 1:5000), α-syn (Abcam, Cambridge, United Kingdom, Cat # ab138501, 1:10000), and tyrosine hydroxylase (Merck Millipore, AB152, 1:5000) at 4 °C overnight. They were then incubated with appropriate secondary antibodies for 1 h before being developed by Fusion FX (M&S Instruments, Osaka, Japan). The amount of protein was estimated using ImageJ software.

### Statistical analysis

Statistical analyses and graph preparation were performed using Prism 7.0 (GraphPad Software, Inc., Boston, MA, United States). Data are presented as the mean and standard deviation (SD). Differences between multiple groups were analyzed using one-way ANOVA followed by Dunnett’s *post-hoc* test for comparisons against the control, or two-way ANOVA followed by Tukey’s *post-hoc* test for factorial designs. Values of *p* < 0.05 were considered statistically significant.

## Results

### High-throughput screening for autophagy inducers in neuronal cells

We developed and optimized a high-throughput phenotypic screening system to identify compounds that induce autophagy in neuronal cells. Figure 1A illustrates the schematic workflow of the screening process. The system used a 384-well plate format. Neurons derived from the fetal rat cerebral cortex were seeded into 384-well plates and cultured for 7 days. Following infection with a rAAV carrying an autophagy flux probe (AAVdj-hSynI-EGFP-LC3B-mCherry), the cells were maintained for an additional 4 days. Compounds were added to a final concentration of 10 μM, and autophagy activation was confirmed 24 h later. The tandem EGFP-LC3-mCherry probe enables quantification of autophagy flux based on the differential pH sensitivity of the two fluorophores. Upon fusion of the autophagosome with the lysosome to form an autolysosome, the acidic lumen selectively quenches EGFP fluorescence while mCherry remains stable. Thus, a reduced GFP/mCherry ratio serves as an indicator of enhanced autophagy flux, facilitating the identification of autophagy-inducing compounds (representative images of fluorescence probes and cell detection for analysis are shown in Supplementary Figures S1A–E).

**Figure 1:**
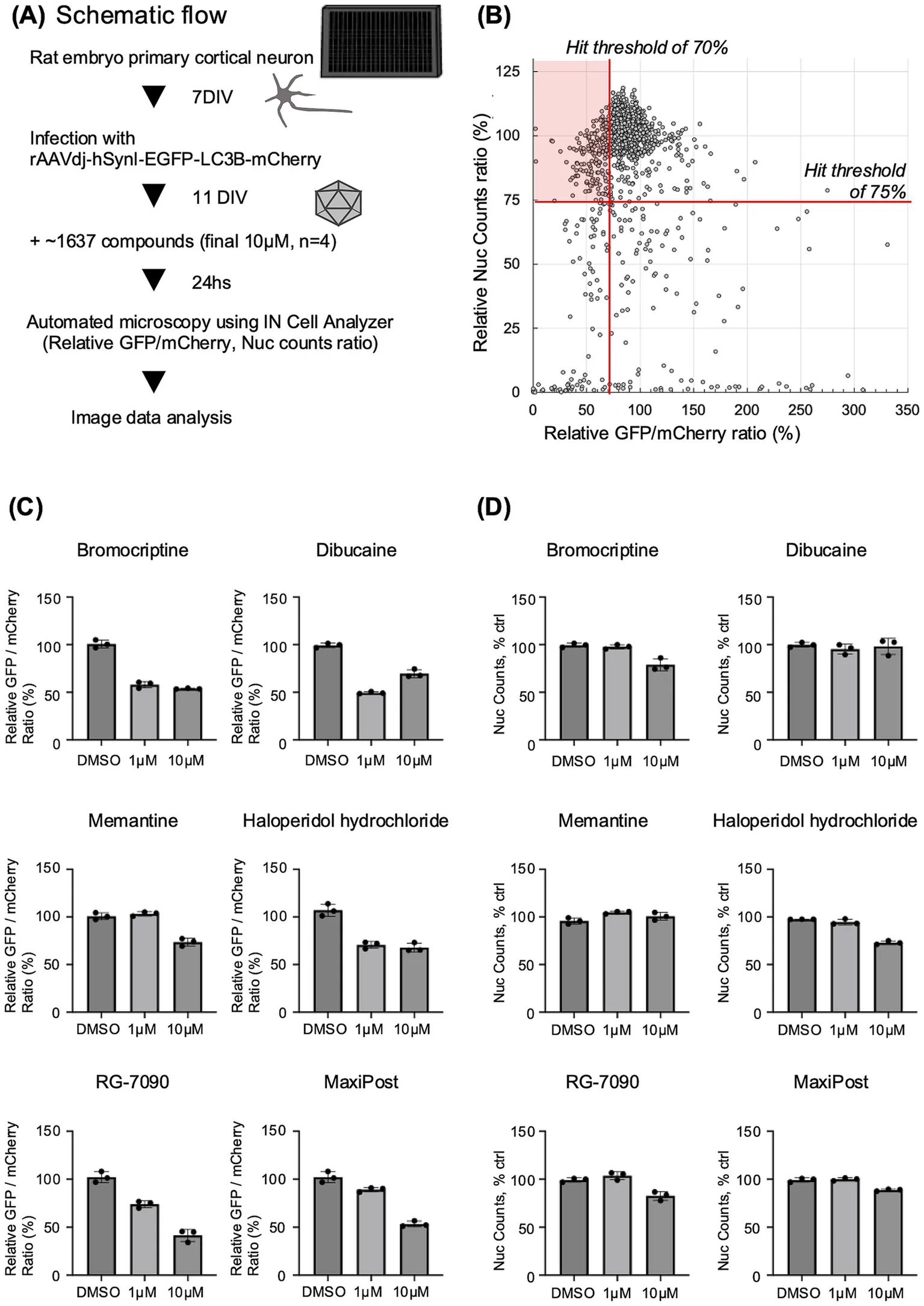
HTS for autophagy-inducing compounds in primary rat cortical neurons using an autophagy flux probe. **(A)** Schematic workflow of the HTS for autophagy inducers. **(B)** Results of the HTS of 1,637 compounds. Each data point corresponds to the mean of technical replicates (*n* = 4). Hit compounds were defined as those with a relative green fluorescent protein (GFP)/mCherry ratio ≤ 70% and a relative nuclear counts ratio ≥ 75% (% of DMSO-treated control), corresponding to the region highlighted in red. **(C)** Relative GFP/mCherry ratio and **(D)** relative nuclear counts ratio of the six hit samples. Data are presented as means ± SD of technical replicates (*n* = 3) from a representative experiment. Similar qualitative trends were confirmed in two independent experiments.

Screening was performed using an autophagic flux probe across four compound libraries (Supplementary Table S1): an FDA-approved drug library (754 compounds), an autophagy-focused library (421 compounds), a kinase-focused library (390 compounds), and a brain-penetrant compound set (72 compounds). Assay performance was evaluated on a plate-by-plate basis using the *Z*′ factor, calculated with dimethyl sulfoxide (DMSO) as the negative control and a structurally undisclosed compound as the positive control, as described by Bar et al. (28). *Z′* factors ranged from 0.04 to 0.69, indicating variable assay performance, with values below 0.5 considered marginal (Supplementary Figure S1F; Supplementary Table S2). Because the primary screen was intended to select candidates for subsequent confirmation, hits from all plates, including those with marginal *Z*′ factors, were retained for retesting. Plate-level *Z*′ factors and the source plates of the confirmed hits are provided in Supplementary Table S2.

Primary hits were defined using two prespecified criteria: (1) a GFP/mCherry ratio of <70% of the DMSO-treated control, indicating autophagy activation; and (2) a relative nuclear count of >75% of the DMSO-treated control, to exclude compounds associated with marked cell loss during the 24-h screening period. The mean number of Hoechst-stained nuclei in the DMSO-treated group was defined as 100%, and nuclear counts in the compound-treated groups were expressed relative to this value. Both thresholds corresponded to four standard deviations below the respective means of the DMSO-treated controls (Figure 1B). A total of 162 compounds met these criteria, corresponding to an overall primary hit rate of 9.9%. All 162 primary hits were subsequently retested using freshly prepared compound solutions. Six compounds reproducibly met the prespecified confirmation criteria, corresponding to a confirmation rate of 3.7%: dibucaine, memantine, and haloperidol from the FDA-approved drug library; bromocriptine from the autophagy-focused library; and RG-7090 and MaxiPost from the brain-penetrant compound set (Figure 1C). In the autophagy-flux probe assay, all six compounds showed autophagy activation at a concentration of 10 μM. At the lower concentration of 1 μM, all compounds except memantine induced autophagy activation. Hoechst-positive nuclear counts were also evaluated during the confirmation assay (Figure 1D). Bromocriptine, haloperidol, RG-7090, and MaxiPost were associated with reduced nuclear counts at one or both concentrations tested, whereas no apparent reduction in nuclear count was observed with dibucaine or memantine under the 24 h screening conditions.

### Secondary evaluation of candidate compounds using endogenous LC3-II

Upon autophagy induction, the cytosolic form of LC3 (LC3-I) is conjugated to phosphatidylethanolamine to form LC3-II, which specifically recruits to autophagosomal membranes. Because LC3-II exhibits faster electrophoretic mobility on sodium dodecyl sulfate-polyacrylamide gel electrophoresis (SDS-PAGE) than LC3-I, the conversion of LC3-I to LC3-II—or the accumulation of LC3-II—serves as a primary hallmark of autophagosome formation. To confirm the results from the autophagy-flux probe assay, we next evaluated endogenous LC3-II levels by western blot analysis. Torin-1 was included as a positive control in these experiments (Figures 2A,B; Supplementary Figure S2). The experiment was performed twice using independently prepared primary neuronal cultures, and the results were interpreted descriptively.

**Figure 2:**
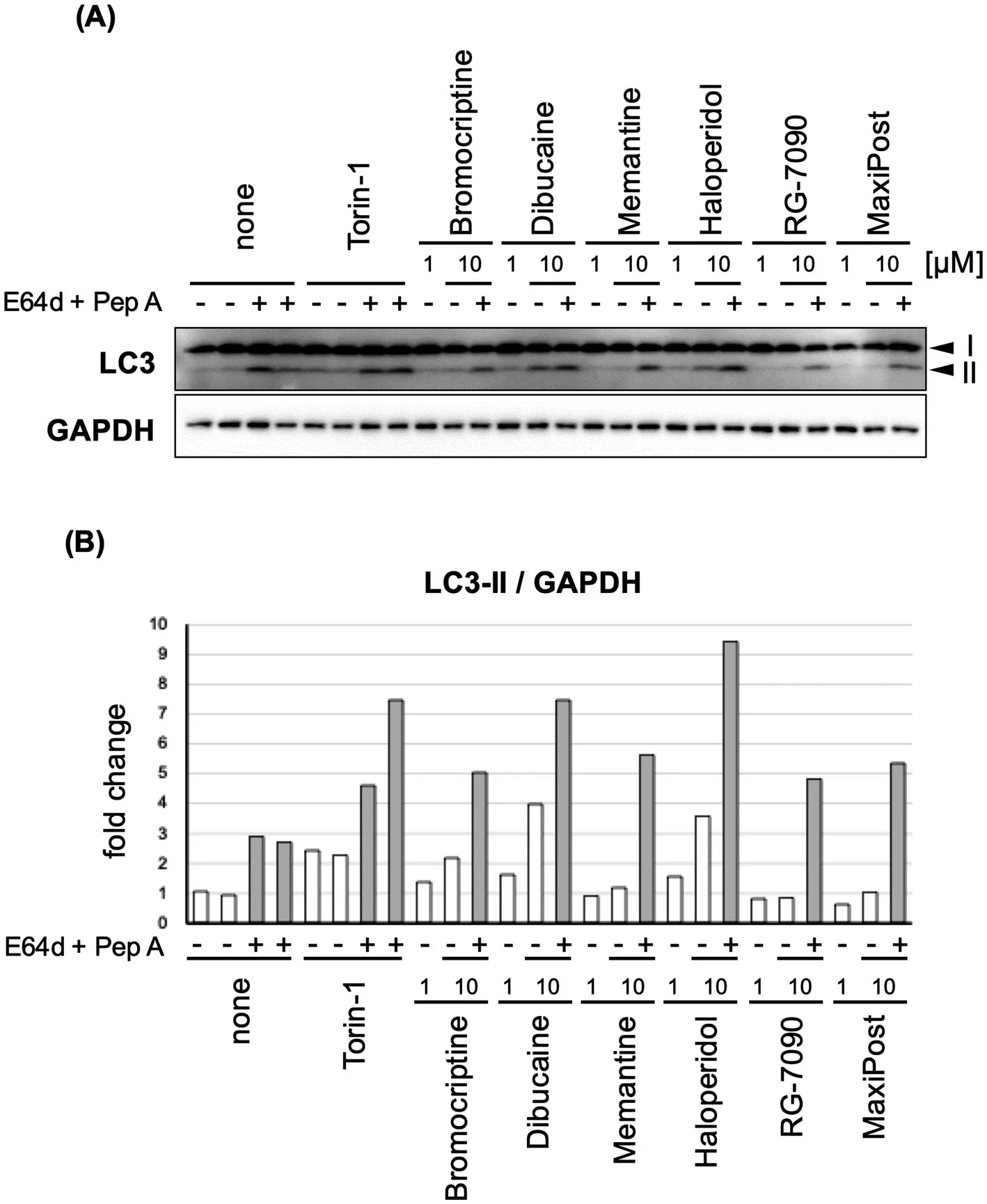
Dose-dependent effects and autophagy-inducing activity of the identified hit compounds in primary rat cortical neurons. Primary rat cortical neurons were treated with hit compounds at 1 or 10 μM, with or without lysosomal protease inhibitors (E64d and Pepstatin A), for 24 h. Torin-1 (0.2 μM) was included as a positive control for autophagy induction. **(A)** Representative western blots of indicated antibodies. **(B)** Quantification of the LC3-II/GAPDH ratio obtained from **(A)**. Data are presented as a representative result from two independent experiments (*n* = 1).

For all six compounds, the increase in the LC3-II/GAPDH ratio at 1 μM was minimal. At 10 μM, dibucaine exhibited the highest effect among the six compounds tested, increasing the LC3-II/GAPDH ratio by approximately three- to four-fold compared with DMSO. Although haloperidol also induced a marked increase in the LC3-II ratio that exceeded the level of Torin-1; however, it was concurrently associated with a reduction in nuclear count in the primary screening assay (Figure 1D). The remaining four compounds exhibited much weaker autophagy-inducing activity than Torin-1. Among the compounds tested, dibucaine combined a reproducible tandem-fluorescence reporter response with increased endogenous LC3-II and no apparent reduction in nuclear count during the 24 h assay, reinforcing its selection as the primary candidate. Next, we assessed the accumulation of LC3-II in the presence of E64d and Pepstatin A, which inhibit lysosomal proteolysis and thereby reduce the degradation of LC3-II after delivery to lysosomes (Figures 2A,B; Supplementary Figure S2). Under these conditions, dibucaine induced only a modest accumulation of LC3-II compared with the other compounds, suggesting that dibucaine triggers autophagy through a mechanism that is distinct from those of the other compounds. However, we cannot rule out the possibility that system saturation prevents a further increase.

Taken together, the tandem-fluorescence reporter response and the endogenous LC3-II results in the presence and absence of lysosomal protease inhibitors were consistent with dibucaine increasing autophagic flux in primary neuronal cultures. Because only two independent experiments were performed, the relative magnitude of the effect compared with the other compounds was treated as exploratory.

### Dibucaine induces autophagic flux and reduces pS129 α-synuclein levels in iPSC-derived dopaminergic neurons

Next, we examined whether dibucaine also induces autophagy in human iPSC-derived dopaminergic neurons. For this purpose, DupA13 iPSCs derived from a familial PD patient (PARK4; duplication of the *SNCA* gene) were differentiated into dopaminergic neurons. Immunofluorescence analysis confirmed successful differentiation, with tyrosine hydroxylase (TH)-positive dopaminergic neurons accounting for 87% ± 2% of the total cell area (Supplementary Figure S3; similar results were obtained in an independent experiment). We then treated these iPSC-derived dopaminergic neurons with dibucaine or Torin-1. Both compounds increased the expression levels of LC3-II in PARK4 iPSC-derived dopaminergic neurons (Figures 3A,B). Dibucaine- or Torin-1-induced LC3-II expression was further increased in the presence of E64d and Pepstatin A, supporting that both compounds enhance autophagic flux in iPSC-derived dopaminergic neurons.

**Figure 3:**
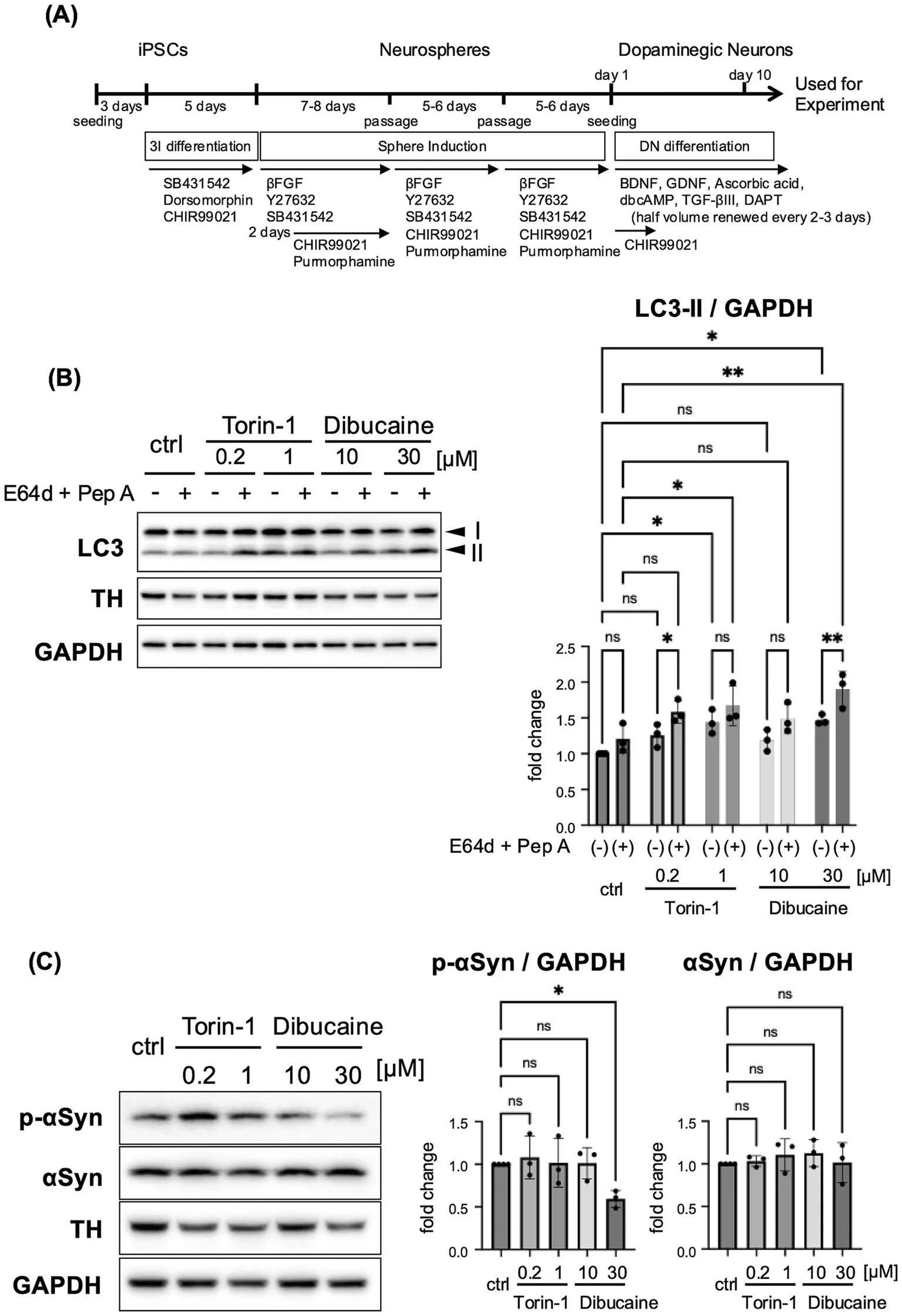
Dibucaine induces autophagy in dopaminergic neurons derived from PARK4 patient iPSCs. **(A)** Schematic diagram of the differentiation protocol from human iPSCs to dopaminergic neurons. **(B,C)** Effects of dibucaine or Torin-1 on autophagy induction and phosphorylated α-syn in PARK4 patient-derived iPSCs (DupA13). DupA13 dopaminergic neurons were treated with dibucaine or Torin-1 for 4 h in the presence or absence of E64d plus Pepstatin A **(B)**, or for 5 days without E64d plus Pepstatin A **(C)**, and the cell lysates were then immunoblotted with the indicated antibodies. Data are presented as mean ± SD. **(B)** Statistical significance was assessed using two-way ANOVA followed by Tukey’s *post-hoc* test for multiple comparisons (*n* = 3, independent biological replicates; * *p* < 0.05, ** *p* < 0.01, *** *p* < 0.001, **** p < 0.0001). **(C)** Statistical significance was assessed using one-way ANOVA followed by Dunnett’s *post-hoc* test for multiple comparisons against a control (*n* = 3–4 independent biological replicates per group; * *p* < 0.05, ** *p* < 0.01, *** *p* < 0.001, **** *p* < 0.0001 versus control).

In PD, α-syn is extensively phosphorylated at Ser129 within synucleinopathic lesions, and this phosphorylation is a hallmark of aggregated α-syn (29, 30). When DupA13 cells were treated with dibucaine for 5 days, phosphorylated α-syn expression was decreased by 30 μM dibucaine (Figure 3C). Although 0.2 and 1 μM Torin-1 also decreased phosphorylated α-syn, a concurrent reduction in tyrosine hydroxylase and GAPDH levels was observed; normalization against GAPDH revealed that the apparent decline in phosphorylated α-syn was not a genuine effect of the treatment. Torin-1 also reduced total protein yield per well, whereas dibucaine treatment (up to 30 μM) showed no reduction in total protein yield (Supplementary Figure S4). Because total protein yield is not a direct measure of cell viability or neuronal integrity, these results do not exclude cytotoxic or other nonspecific cellular effects of long-term dibucaine treatment.

These results suggest that dibucaine induced autophagic flux and was associated with a reduction in the cellular pS129 α-syn signal. However, the present experiments do not distinguish autophagic degradation of pS129 α-syn from reduced Ser129 phosphorylation.

### Effects of dibucaine-related local anesthetics on autophagy-associated readouts in SH-SY5Y cells

To investigate signaling events associated with the cellular response to dibucaine in an experimentally tractable human cell system, we used SH-SY5Y neuroblastoma cells. Following treatment with 30 μM dibucaine for 4 h, no apparent reduction in total protein yield per well was observed relative to vehicle control (Supplementary Figure S4). This measurement was used as a gross assessment of protein recovery and does not constitute a direct evaluation of cell viability. Dibucaine is a local anesthetic that blocks sodium channels (31). We therefore examined the effects of other local anesthetics on autophagy induction in SH-SY5Y cells. In two independent experiments, proparacaine, lidocaine, and tetracaine produced smaller increases in LC3-II abundance than 30 μM dibucaine; these findings were interpreted descriptively (Figure 4A; Supplementary Figure S5A). These findings suggest that the effect of dibucaine on LC3-II accumulation might not be solely attributable to sodium-channel blockade.

**Figure 4:**
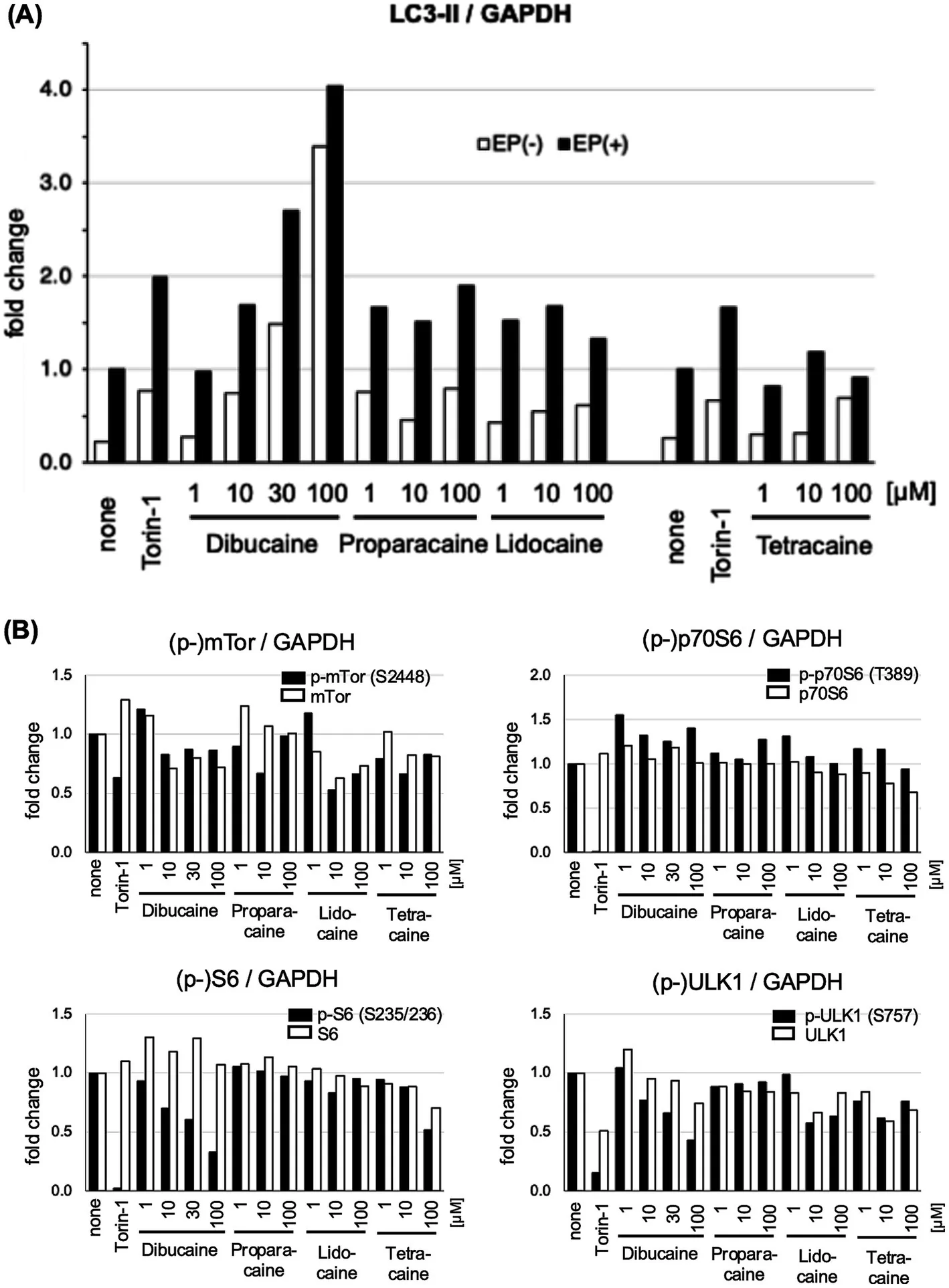
Effects of dibucaine-related local anesthetics on autophagy induction in SH-SY5Y cells. Quantification of the relative expression levels of the indicated proteins in SH-SY5Y cells treated with local anesthetics structurally related to dibucaine for 4 h **(A)** or 24 h **(B)**. Corresponding western blot images are shown in Supplementary Figures S5A,B. Data are presented as representative results from two independent experiments (*n* = 1).

### Dibucaine induces autophagy without broad suppression of canonical mTORC1 signaling

mTOR inhibitors such as rapamycin and Torin-1 are the best-understood inducers of autophagy. mTORC1 negatively regulates autophagy by phosphorylating ULK1 at Ser757, which is required for autophagosome formation (18). We therefore examined the effects of dibucaine on canonical mTORC1-associated phosphorylation markers. Torin-1 (0.2 μM) markedly reduced the phosphorylation signals of mTOR, p70S6K, S6, and phosphorylated ULK1 at Ser757 (Figure 4B; Supplementary Figure S5B), consistent with its known effect of mTOR inhibition. In two independent experiments, dibucaine (30 μM) did not appreciably reduce the phosphorylation of mTOR at Ser2448 or p70S6K but partially reduced the phosphorylation of S6 Ser235/236 and ULK1 at Ser757 (Figure 4B; Supplementary Figure S5B). Because these phosphorylation events can also be influenced by non-mTOR kinases, phosphatases, and signaling crosstalk, the partial changes do not necessarily indicate broad mTORC1 inhibition (32–34). These findings are consistent with dibucaine inducing autophagy without broad suppression of canonical mTORC1 signaling, although a contribution from partial attenuation of mTORC1 signaling cannot be excluded.

### Dibucaine increases AMPK Thr172 phosphorylation during autophagy induction

Previously, we have reported that several protein kinase inhibitors induce autophagy or modulate its induction depending on the type of autophagy-inducing stress that is present (35). To explore signaling events associated with dibucaine-induced autophagy, we screened several well-characterized protein kinase inhibitors (Supplementary Table S3) to identify compounds that were able to suppress this process. In this initial screen, LY364947, Janus kinase 3 (JAK3) inhibitor VI, AG1478, sorafenib, and Compound C were associated with smaller dibucaine-induced LC3-II responses (Figure 5A). Because this screen was performed once, these findings were treated as exploratory and were not subjected to inferential statistical analysis.

**Figure 5:**
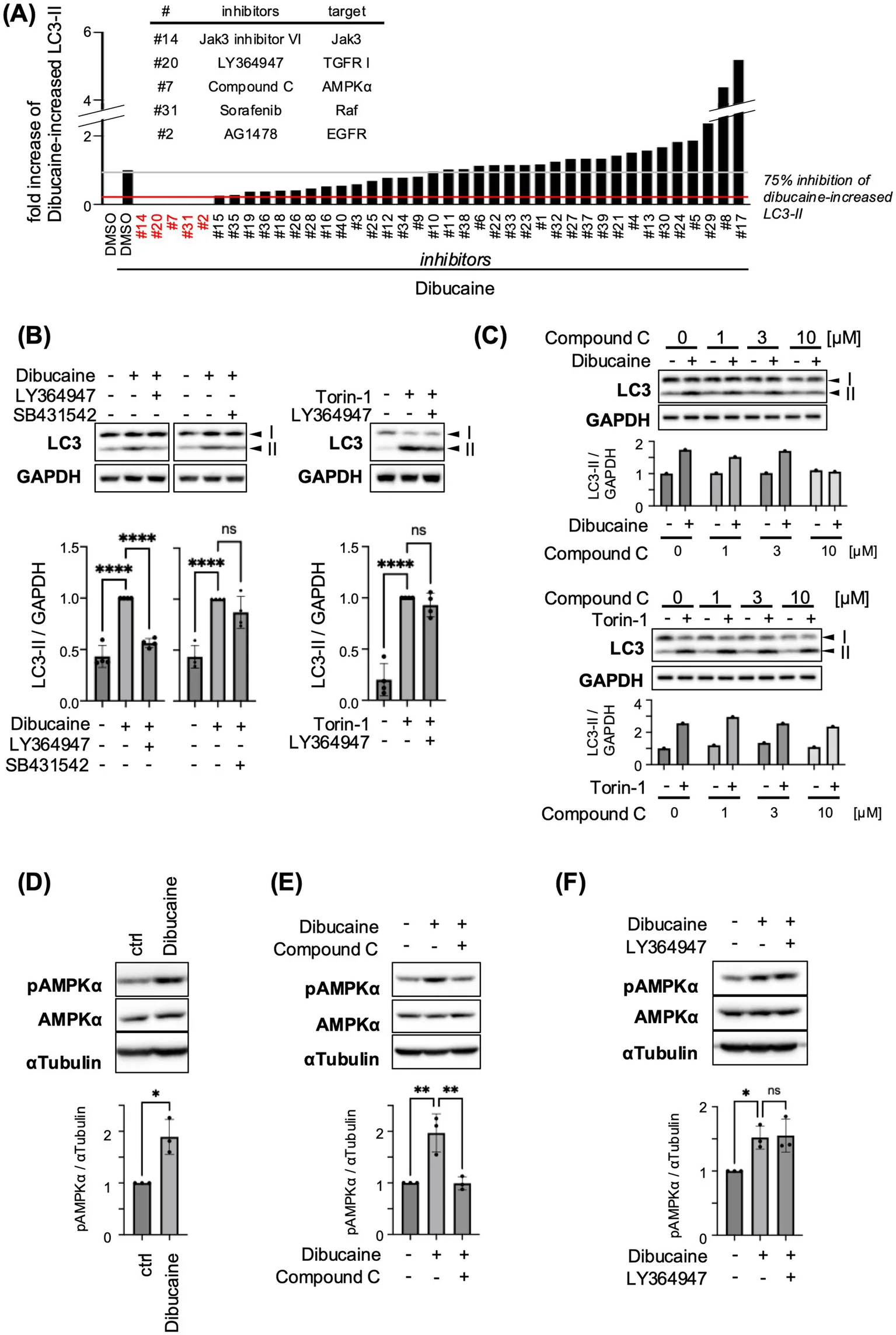
Effects of protein kinase inhibitors on dibucaine-induced autophagy in SH-SY5Y cells. **(A)** SH-SY5Y cells were pretreated with various protein kinase inhibitors at 10 μM (except for #18 LiCl, 2 mM) for 1 h, followed by incubation with dibucaine for an additional 4 h. Cell lysates were then subjected to immunoblotting using antibodies against LC3 and GAPDH. The graph shows the relative LC3-II/GAPDH levels for each inhibitor, normalized to the value of dibucaine-stimulated cells (set as 1.0). **(B,C)** SH-SY5Y cells were pretreated with 10 μM LY364947 or 10 μM SB431542 **(B)** or indicated concentrations of Compound C **(C)** for 1 h, followed by incubation with 30 μM dibucaine or 0.2 μM Torin-1 for an additional 4 h. Cell lysates were then subjected to immunoblotting using antibodies against LC3 and GAPDH. **(D)** SH-SY5Y cells were treated with 30 μM dibucaine for 2 h, and cell lysates were then immunoblotted with the indicated antibodies. **(E,F)** SH-SY5Y cells were pretreated with 10 μM Compound C **(E)** or 10 μM LY364947 **(F)** for 15 min followed by incubation with 30 μM dibucaine for 2 h. Representative western blot images are shown. Quantitative data in **(B,D–F)** represent the mean ± SD from three biological replicates (*n* = 3), whereas data in **(A,C)** show results from a single experiment (*n* = 1). Statistical analysis was performed using one-way ANOVA followed by Dunnett’s test for **(B,E,F)**, and Welch’s *t*-test for **(D)**. * *p* < 0.05, ** *p* < 0.01, *** *p* < 0.001, **** *p* < 0.0001 versus control.

Among these protein kinase inhibitors, LY364947 (Figure 5B) and Compound C (Figure 5C) did not inhibit Torin-1-associated LC3-II response, whereas JAK3 inhibitor VI, AG1478, and sorafenib reduced the LC3-II responses associated with both dibucaine and Torin-1 (Supplementary Figure S6). These pharmacological profiles suggested that LY364947- and Compound C-sensitive cellular processes may differ between dibucaine and Torin-1 treatment.

Compound C has been widely used as pharmacological inhibitor of AMPK (36–39), but is also known to affect multiple other kinases and cellular processes. Dibucaine treatment increased phosphorylation of the AMPK α-subunit at Thr172 in SH-SY5Y cells, an activating phosphorylation site (Figure 5D) (40, 41). Compound C reduced the dibucaine-associated the increase in AMPK Thr172 phosphorylation (Figure 5E). Because Compound C altered AMPK Thr172 phosphorylation itself, its effect cannot be interpreted as a selective test of whether AMPK activity is required for dibucaine-induced autophagy. The result may reflect an effect on an upstream or parallel signaling event, phosphatase activity, cellular energy status, or another off-target process. Compound C was therefore not used to draw a mechanistic conclusion regarding AMPK dependence. The present experiments show that dibucaine treatment is associated with increased AMPK Thr172 phosphorylation but do not establish that AMPK is required for dibucaine-induced autophagic flux.

By contrast, LY364947 exerted only a minimal inhibitory effect on dibucaine-induced AMPK phosphorylation at Thr172 (Figure 5F). Although LY364947 is a potent and well-characterized inhibitor of transforming growth factor-beta (TGF-beta) receptor type-1 (also known as ALK5), another ALK5 inhibitor (SB431542) failed to inhibit dibucaine-induced autophagy (Figure 5B). Consistent with these findings, dibucaine still induced autophagy in ALK5-knockdown SH-SY5Y cells (Supplementary Figure S7), suggesting that LY364947 inhibits dibucaine-induced autophagy through an ALK5-independent mechanism.

Given that LY364947 is also reported to inhibit vascular endothelial growth factor receptor (VEGFR), receptor-interacting serine/threonine-protein kinase 2 (RIPK2), and casein kinase I isoform delta (CK1δ) (39), we examined the potential involvement of these kinases in dibucaine-induced autophagy. Of the three kinases, VEGFR was excluded because the VEGFR inhibitor SU11652 did not inhibit dibucaine-induced autophagy (Figure 5A, compound #34). The knockdown of RIPK2 or CK1δ also failed to inhibit dibucaine-induced autophagy (Supplementary Figure S7). These negative findings did not support a major contribution from the tested kinases but do not definitively exclude their involvement. The LY364947-sensitive molecular target responsible for the observed effect therefore remains unidentified.

### Effects of LY364947 on dibucaine-associated LC3-II and pS129 α-synuclein responses in PARK4 iPSC-derived dopaminergic neurons

Next, we examined whether LY364947 can modulate dibucaine-induced autophagic-flux and the reduction of phosphorylated α-syn in human PARK4 (Dup A13) iPSC-derived dopaminergic neurons. Dibucaine increased LC3-II abundance in the presence and absence of LY364947. LY364947 partially attenuated the magnitude of this increase, whereas SB431542 did not show a comparable effect (Figure 6A). Within the LY364947-treated group, the relative LC3-II signal increased from 0.88 ± 0.11 without dibucaine to 1.28 ± 0.19 with dibucaine (mean ± SD, *n* = 6). Thus, LY364947 attenuated but did not abolish the dibucaine-associated LC3-II response. LY364947 was also associated with attenuation of the reduction in pS129 α-syn observed following treatment with 30 μM dibucaine for 5 days (Figure 6B). However, the LY364947 results demonstrate a pharmacological association between an unidentified LY364947-sensitive cellular process, the LC3-II response, and the change in pS129 α-syn. They do not establish that LY364947 specifically inhibits autophagic flux or that the reduction in pS129 α-syn is mediated by autophagic degradation.

**Figure 6:**
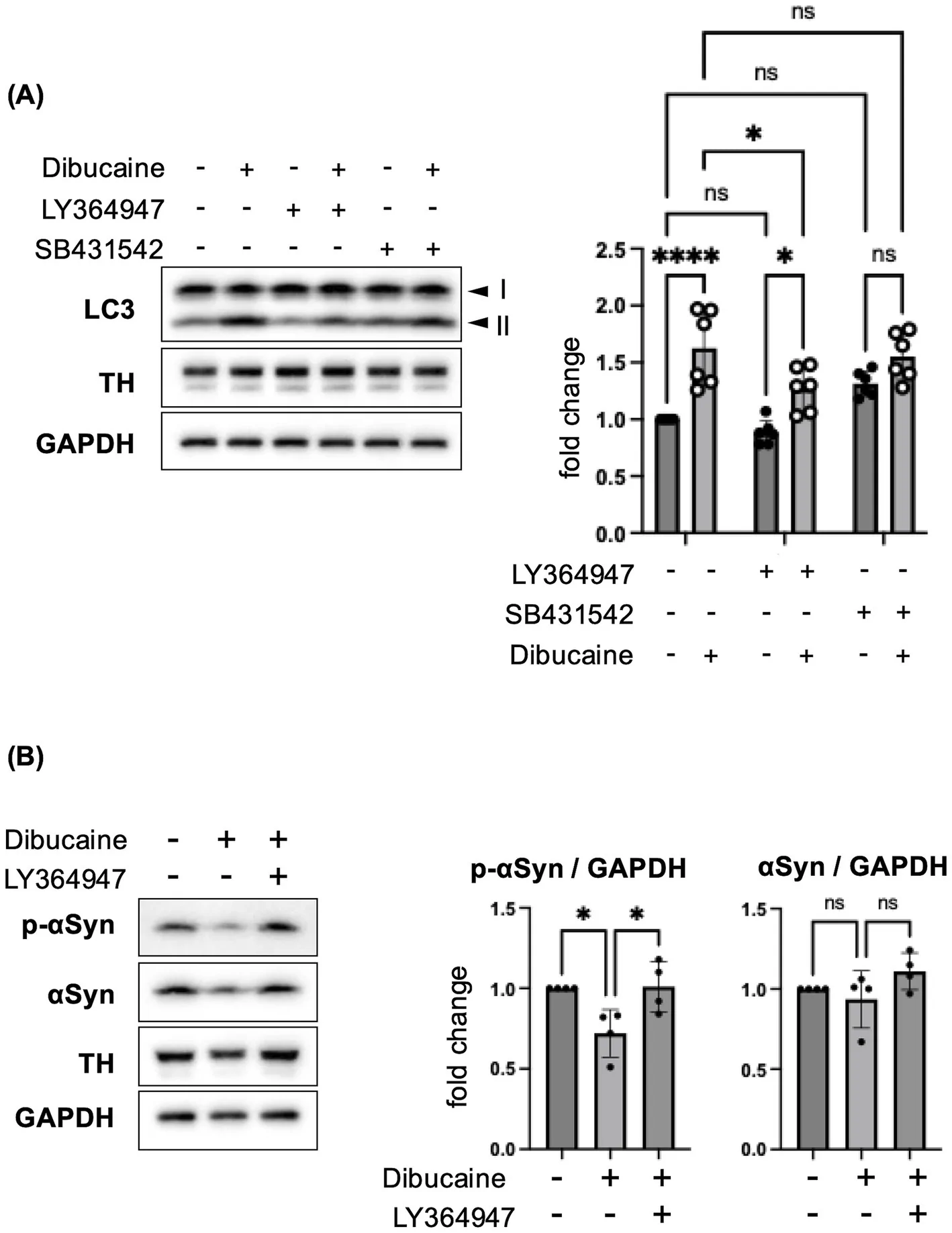
Effects of LY364947 on dibucaine-induced autophagy and reduction in phosphorylated α-syn levels in human PARK4 iPSC–derived dopaminergic neurons. DupA13 neurons were treated with 30 μM dibucaine in the presence or absence of 10 μM LY364947 or 10 μM SB431542 for 4 h in the presence of E64d plus Pepstatin A **(A)**, or for 5 days without E64d plus Pepstatin A **(B)**, and the cell lysates were then immunoblotted with the indicated antibodies. Representative western blot images are shown. Quantitative data show the relative levels of LC3-II/GAPDH for **(A)**, and p-αSyn/GAPDH as well as αSyn/GAPDH for **(B)**. Data are presented as mean ± SD (*n* = 6 for A; *n* = 4 for B, independent biological replicates). Statistical significance was assessed using two-way ANOVA followed by Tukey’s *post-hoc* test for multiple comparisons for **(A)**, and one-way ANOVA followed by Dunnett’s *post-hoc* test for multiple comparisons against the control for **(B)** (* *p* < 0.05, ** *p* < 0.01, *** *p* < 0.001, **** *p* < 0.0001).

## Discussion

Impairment of the autophagy-lysosome pathway is a central pathological feature of PD, prompting extensive efforts to identify small-molecule autophagy inducers. Over the past decades, several classes of compounds have been explored to enhance autophagic clearance of toxic α-synuclein species. These include mTOR inhibitors such as rapamycin, mTOR-independent autophagy enhancers such as the disaccharide trehalose, and repurposed clinical drugs such as metformin and the tyrosine kinase inhibitor nilotinib (16). Although these compounds have demonstrated neuroprotective potential in various preclinical models, many face translational hurdles, including limited blood–brain barrier permeability, low potency requiring high millimolar concentrations, or off-target toxicities. Consequently, identifying novel, brain-penetrable chemical scaffolds that potently stimulate autophagic flux in human neuronal models remains a critical priority. Reports of HTS studies with a focus on identifying autophagy-inducing compounds in neurons or neuron-like cells remain relatively scarce (42–46). Importantly, most previous studies have relied on a limited range of cellular models and have not systematically evaluated species-specific differences or translational relevance to human disease. Here, we established a novel HTS platform using rat embryonic primary neurons to identify autophagy-activating compounds and performed a screening campaign.

An important strength of the HTS platform developed in this study is the use of rat embryonic primary neurons, which preserve the intrinsic physiological characteristics, neuronal functions, and synaptic networks of the brain more faithfully than immortalized cell lines. This feature allows accurate assessment of drug-induced modulation of autophagy as well as marked cell loss. Nevertheless, plate-level *Z*′ factors varied considerably, ranging from 0.04 to 0.69 (Supplementary Figure S1F), indicating assay performance ranging from marginal to robust. This variability may reflect biological and technical factors, including differences among independently prepared primary neuronal cultures, cell dissociation, and cell plating. Only six of the 162 primary hits were confirmed on retesting (3.7%), underscoring that the primary screen served as a candidate-selection step requiring independent validation. Greater standardization of neuronal preparation and plating, together with stricter plate-level quality control, will be required to improve the robustness and reproducibility of the screening platform.

The identified compounds were subsequently validated in human iPSC-derived neurons and patient-derived neuronal cells. This strategy enabled the identification of autophagy-inducing compounds that are effective not only in healthy neuronal cells but also in patient-derived cells, exhibiting minimal species-dependent variability. Using this approach, we identified dibucaine as a compound that consistently induced autophagy in neurons derived from both rat and human iPSCs. Together, these findings indicate that dibucaine induces autophagic flux in both rat primary neurons and human iPSC-derived neurons, including PARK4 patient-derived dopaminergic neurons.

Dibucaine is a potent amide-type local anesthetic that has been widely used in clinical practice for topical and spinal anesthesia. Its primary mechanism of action is the reversible blockade of voltage-gated sodium channels, thereby inhibiting the initiation and conduction of nerve impulses (47).

In addition to its established role as a local anesthetic, dibucaine has been used experimentally as a calpain activator at a relatively high concentration (500 μM), indicating pharmacological activity beyond sodium channel modulation (48), expanding its potential utility as a functional tool to modulate cellular stress responses. In our comparative analysis, dibucaine produced a greater LC3-II response than other widely used local anesthetics, including proparacaine, lidocaine, and tetracaine. These findings suggest that the response to dibucaine may not be a general class effect shared by the tested local anesthetics and might not be solely attributable to sodium-channel blockade. Rather, dibucaine-specific structural features, including its lipophilic quinoline core and substituted side chain, may contribute to this activity, although a structure–activity relationship remains to be established.

Of the two major pathways that control autophagy, the mTOR-dependent pathway has traditionally been considered the most promising therapeutic target because its inhibition can strongly modulate autophagy. However, mTORC1 has autophagy-independent functions, meaning that targeting mTORC1 may lead to adverse effects in patients with immunosuppression and impaired wound healing processes. Consequently, research focus has increasingly shifted toward mTOR-independent autophagy-inducing compounds. These agents offer targeted therapeutic opportunities for neurodegenerative disorders such as PD without compromising global metabolic signaling. Within this framework, we revealed that dibucaine induces autophagy in an mTOR-independent manner, although a contribution from partial attenuation of mTORC1 signaling cannot be excluded.

Dibucaine treatment was also associated with reduced levels of phosphorylated α-syn in dopaminergic neurons derived from PARK4 patient iPSCs. The induction of autophagic flux and the reduction in pS129 α-syn were experimentally observed in the same cellular model; however, a causal relationship between these findings was not demonstrated.

We acknowledge that in current iPSC-derived neuronal models, the formation of mature, insoluble α-syn aggregates typically requires prolonged maturation over many weeks. Under our culture conditions, robust insoluble α-syn aggregates were not readily observed in the *SNCA* duplication iPSC-derived neurons. Therefore, this study evaluated changes in the cellular levels of pS129 α-synuclein and did not directly assess the clearance of mature α-synuclein aggregates.

Dibucaine induced autophagic flux and increased AMPK Thr172 phosphorylation in SH-SY5Y cells. Recent studies have increasingly highlighted the therapeutic potential of AMPK activators in the treatment of neurodegenerative disorders, particularly PD. AMPK acts as a master regulator of cellular energy homeostasis and plays a critical role in the clearance of protein aggregates via the autophagy–lysosome pathway (49–51). Its activation requires the phosphorylation of Thr172 within the activation loop of its catalytic α-subunit by upstream kinases. In PD models, the pharmacological activation of AMPK by small molecules, such as metformin or 5-aminoimidazole-4-carboxamide ribonucleoside, reduces α-syn accumulation and mitigates dopaminergic neurodegeneration (52). Furthermore, direct AMPK activators reportedly enhance mitophagy and improve mitochondrial biogenesis, thereby addressing the bioenergetic deficits that are central to PD pathogenesis (53). At present, the relationship between AMPK activation and dibucaine-induced autophagic flux remains associative.

Several upstream kinases responsible for AMPK phosphorylation/activation have previously been identified. Serine/threonine-protein kinase STK11 (also known as LKB1) (54, 55), Ca^2+^-activated Ca^2+^/calmodulin-dependent kinase kinase β (56–58), and transforming growth factor β-activated kinase-1 (59) are well-studied upstream kinases that activate AMPK through the phosphorylation of Thr172. The present experiments did not identify the upstream event responsible for the dibucaine-associated increase in AMPK phosphorylation. Moreover, because the functional requirement for AMPK has not yet been established, identification of the relevant upstream kinase will require additional studies.

Notably, AMPK is not required for Torin-1-induced autophagy (60). Consistent with the idea that α-syn overexpression inhibits AMPK activity through LKB1 inhibition (61, 62). However, the differential responses to dibucaine and Torin-1 observed in the present study do not demonstrate that dibucaine promotes pS129 α-syn turnover through AMPK. Accordingly, the comparison with Torin-1 does not establish an AMPK-specific mechanism for the reduction in pS129 α-syn.

Although LY364947 attenuated autophagic flux induced by dibucaine but not Torin-1, it had little effect on dibucaine-induced AMPK activation. These results suggest that the inhibitory effects of LY364947 are likely exerted downstream of AMPK activation and are independent of—or divergent from—the Torin-1-targeted pathway. However, silencing ALK5—the primary target of LY364947—as well as other kinases known to be inhibited by this compound, such as RIPK2 and CK1δ, failed to suppress dibucaine-induced autophagy. Broad kinase profiling studies have shown that LY364947 can inhibit several additional kinases, including TGFBR2 and p38 MAPK, particularly at higher concentrations or in specific cellular contexts. It is therefore possible that the concurrent inhibition of multiple redundant kinases, or an interaction with a yet-to-be-identified protein, is required for this suppressive effect. To identify the precise molecular target(s), unbiased approaches such as photo-affinity chemical proteomics, thermal proteome profiling (TPP), or genome-wide CRISPR screens will be essential to clarify the downstream signaling components of dibucaine-induced autophagy.

Although its mTOR-independent induction of autophagy represents an attractive chemical property *in vitro*, dibucaine itself has clear limitations as a direct therapeutic candidate for PD due to its local anesthetic toxicity. Therefore, rather than pursuing its direct clinical application, medicinal chemistry optimization aimed at dissociating its autophagy-modulating effects from its local anesthetic activity and toxicity will be necessary to explore the therapeutic potential of this pathway.

Several limitations of this study warrant consideration. Most notably, our findings rely entirely on *in vitro* cellular models, and *in vivo* validation in animal models of PD has not yet been conducted. In addition, the effects of prolonged dibucaine treatment on neuronal viability and integrity were not comprehensively evaluated. Pharmacokinetic properties—such as blood–brain barrier permeability, metabolic stability, and systemic toxicity—were not evaluated in this study. Further *in vivo* studies combined with comprehensive pharmacokinetic profiling will be necessary to determine whether this strategy can be translated into a clinically viable therapy.

## Data Availability

The original contributions presented in the study are included in the article/Supplementary material, further inquiries can be directed to the corresponding authors.

## Footnotes

1
http://www.jove.com/video/3965/
