# Supplementary material for: Dibucaine induces autophagic flux and reduces Ser129-phosphorylated α-synuclein in induced pluripotent stem cell–derived neurons with SNCA duplication
Source: Front Neurol. 2026 Oct 8;17:1891393. doi: 10.3389/fneur.2026.1891393 (PMC13686833; doi:10.3389/fneur.2026.1891393)

Supplementary Methods

siRNA Transfection

Transfection of SH-SY5Y cells with siRNAs was performed using Lipofectamine RNAiMax (Thermo Fisher Scientific) in accordance with the manufacturer's instructions. The siRNAs targeted the following genes: ALK5 (TGF-beta receptor I; SASI_Hs01_00181142) and a negative control were purchased from Sigma-Aldrich; RIPK2 construct #6 (SI02655254, Hs_RIPK2_6) and construct #8 (SI02758819, Hs_RIPK2_8), as well as Casein kinase 1 delta (CK1δ) construct #3 (SI00029603, Hs_CSNK1D_3) and construct #5 (SI00287406, Hs_CSNK1D_5), were obtained from Qiagen.

Antibodies

Protein knockdown was verified by western blotting using the following primary antibodies: anti-ALK5 (TGF-beta receptor I; abcam, Cambridge, UK; ab235578, clone EPR209234-13), anti-RIPK2 (RIP2; Cell Signaling Technology, Danvers, MA, USA; #4142S, clone D10B11), and anti-Casein kinase 1 delta (Santa Cruz Biotechnology, Dallas, TX, USA; sc-55553, clone C-8).

Immunofluorescence Analysis

Differentiated human iPSC-derived dopaminergic neurons (DupA13) were fixed and stained for TH (green) and Hoechst 33342 (blue). Fluorescence images were captured using a BZ-X800 microscope (Keyence, Osaka, Japan). To evaluate differentiation efficiency, the TH-positive area within the Hoechst-positive total cell area (ROI) was quantified from four microscopic fields ($n = 4$) using ImageJ software.

Supplementary Table S1. Summary of the focused compound libraries and the number of compounds included in each library

Commercial Library	number
Autophagy focused Lib. (MedChemExpress)	421
FDA approved Lib. (Prestwick)	754
Kinase Lib. (Selleck)	390
CNS select Lib. (FDA approved or clinical fail)	72

Supplementary Table S2. Hit compounds identified from HTS in primary rat brain neurons with corresponding plate *Z*' factors

Compound	Plate number	*Z*' factor
Bromocriptine	#7	0.31
Dibucaine	#2	0.48
Memantine	#9	0.39
Haloperidol hydrochloride	#2	0.48
RG-7090	#21	0.51
MaxiPost	#21	0.51

Supplementary Table S3. List of protein kinase inhibitors used in the experiments shown in Figure 5.

#	inhibitors	target	test conc.
#1	A-83-01	ALK4,5	10μM
#2	AG1478	EGFR	10μM
#3	AGL2263	IGF-IR	10μM
#4	AZD1480	JAK1/2	10μM
#5	BI2536	PLK1	10μM
#6	CGI1746	Btk	10μM
#7	CompoundC	AMPK	10μM
#8	Crizotinib	c-Met/Alk	10μM
#9	Ex527	Sirt1	10μM
#10	Goe6976	PKC	10μM
#11	GSK180936	GRK5	10μM
#12	GSK2656157	PERK	10μM
#13	IWP-2	WNT	10μM
#14	JAK3iVI	JAK3	10μM
#15	KN92	nega	10μM
#16	KN93	CaMK	10μM
#17	LDN193189	ALK(BMPsignal)	10μM
#18	LiCl	GSK3b	20mM
#19	LY294002	PI3K	10μM
#20	LY364947	TGFR etc..	10μM
#21	MK6-83	TRPML1	10μM
#22	ML385	NRF2	10μM
#23	MLi-2	LRRK2	10μM
#24	PDGFRinV	multiTK	10μM
#25	Ruxolitinib	JAK1/2	10μM
#26	SB203580	p38	10μM
#27	SB415286	GSK3b	10μM
#28	SB431542	TGFR	10μM
#29	SB590885	Raf	10μM
#30	Silmitasertib	CK	10μM
#31	Sorafenib	Raf	10μM
#32	SP600125	JNK	10μM
#33	STF083010	IRE1	10μM
#34	SU11652	RTK(FGFR,VEGFR...)	10μM
#35	SU9516	CDK	10μM
#36	Tofacitinib	JAKs	10μM
#37	TSA	HDAC	10μM
#38	U0126	MEK	10μM
#39	XAV939	PARP	10μM
#40	Y27632	Rock	10μM

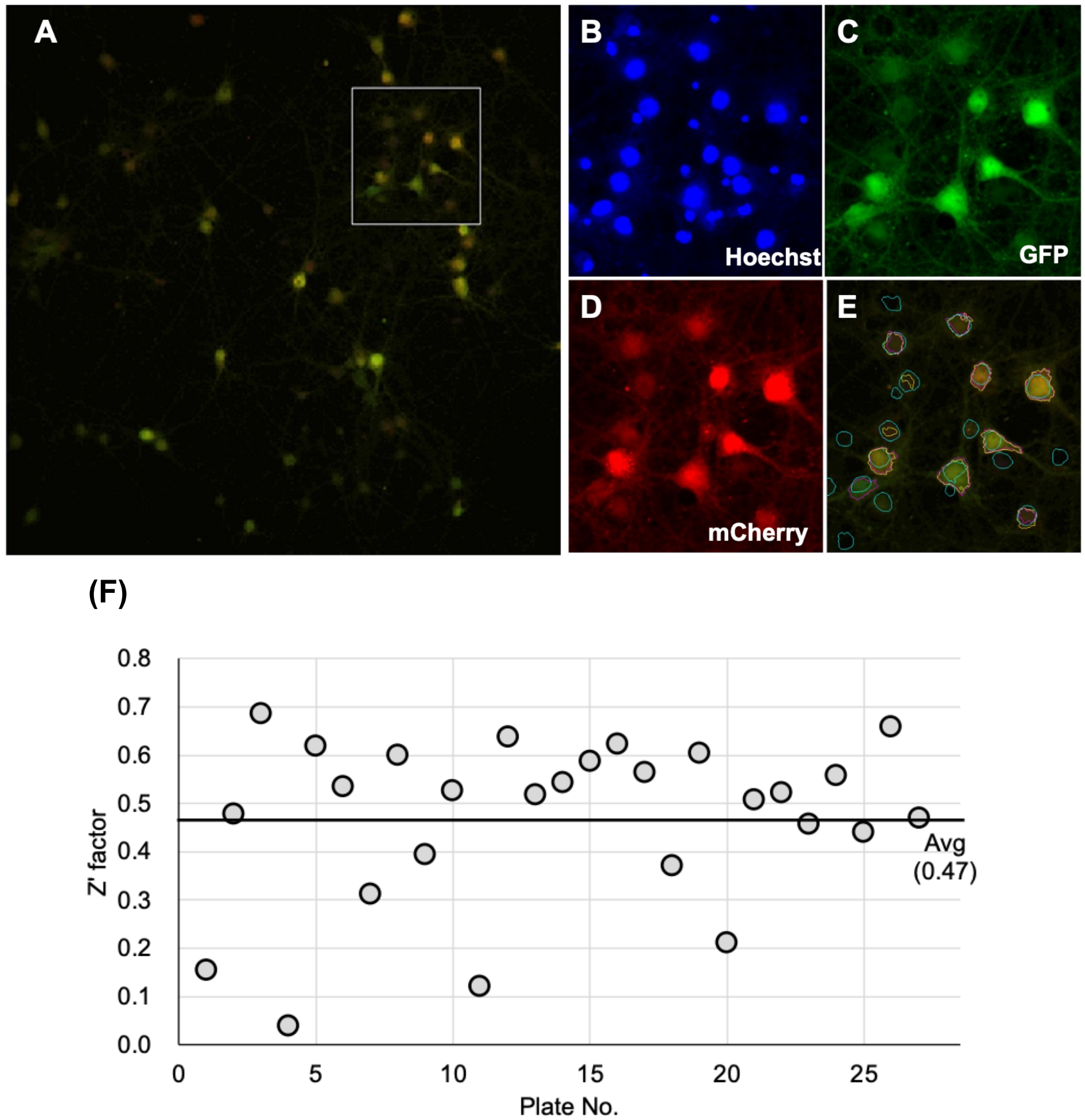

Supplementary Figure S1. High-throughput screening assay performance and image analysis for autophagy flux quantification.

(A-E) Representative fluorescence images and automated cell detection using the IN Cell Investigator Developer software. Primary rat cortical neurons expressing the tandem EGFP-LC3-mCherry flux probe were stained with Hoechst 33342. Shown are merged (A) and enlarged views of nuclei (B; blue), EGFP (C; green), and mCherry (D; red) signals, along with the corresponding segmentation outlines (E) used for object identification and intensity quantification (20× objective). (F) *Z*' factors of individual plates from HTS of autophagy-inducing compounds in primary rat cortical neurons using an autophagic flux probe. The x-axis represents the plate number, and the y-axis represents the *Z*' factor.

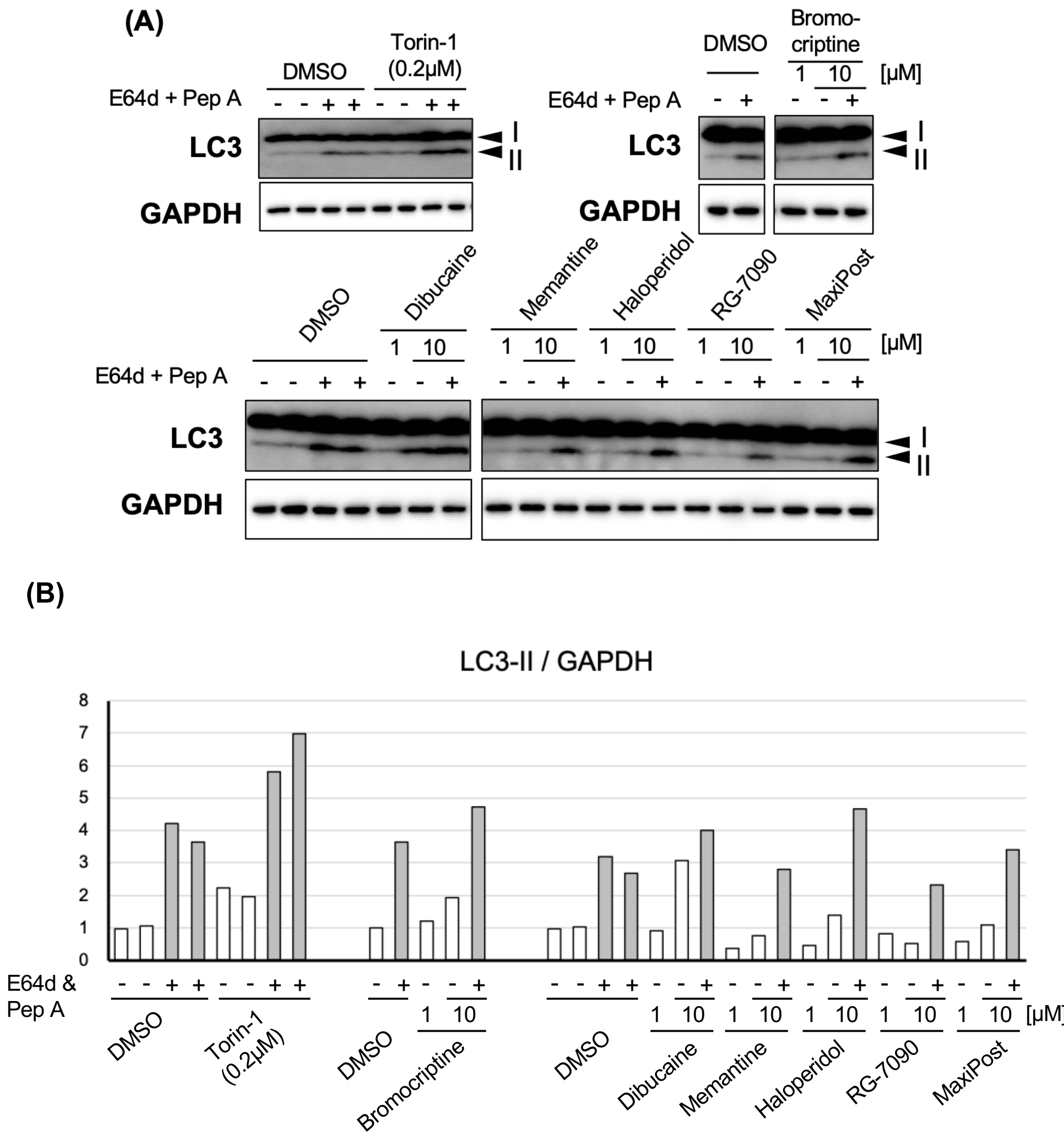

Supplementary Figure S2. Reproducible autophagy-inducing activity of the identified hit compounds in primary rat cortical neurons

Primary rat cortical neurons were treated with hit compounds at 1 or 10 µM, with or without lysosomal protease inhibitors (E64d and Pepstatin A), for 24 hours. Torin-1 (0.2 µM) was included as a positive control for autophagy induction. (A) Western blot analysis showing LC3 and GAPDH levels. (B) Quantification of the LC3-II/GAPDH ratio obtained from (A). Data are presented as a representative result from two independent experiments (*n* = 1).

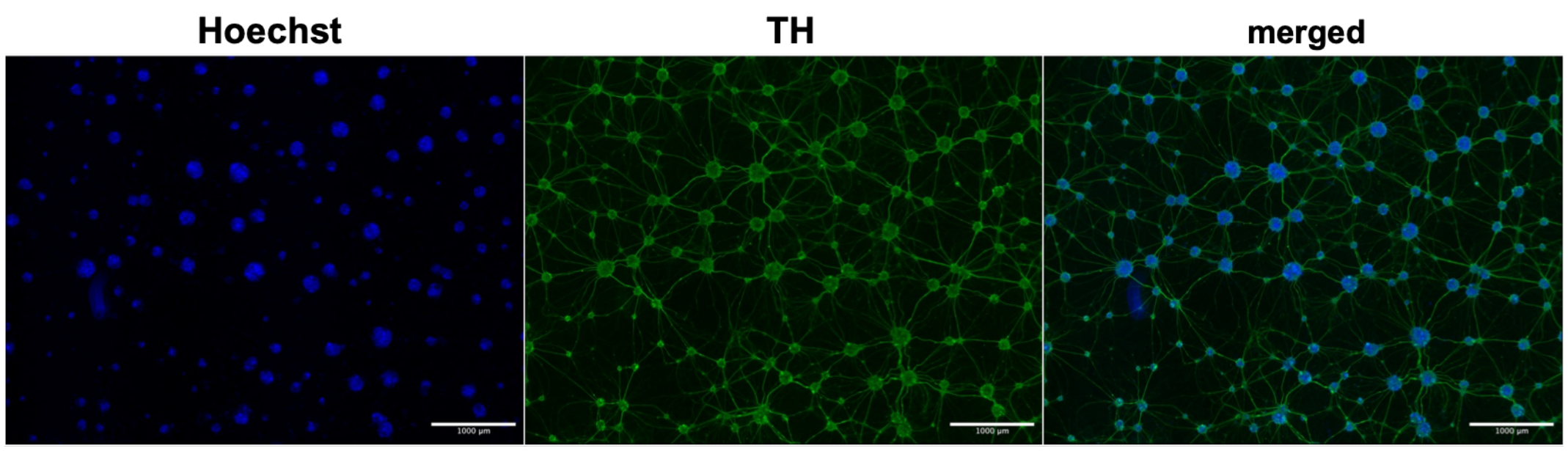

Supplementary Figure S3. Characterization of DupA13-derived dopaminergic neurons

Representative immunofluorescence images of differentiated human iPSC-derived dopaminergic neurons (DupA13) stained for Hoechst 33342 (blue; nuclei) and tyrosine hydroxylase (TH; green; dopaminergic neuronal marker), along with the merged image. Scale bar, 1000 µm. Quantitative analysis revealed that TH-positive areas accounted for 87% ± 2% of the total cell area (n = 4 microscopic fields from a representative experiment; similar results were obtained in an independent experiment).

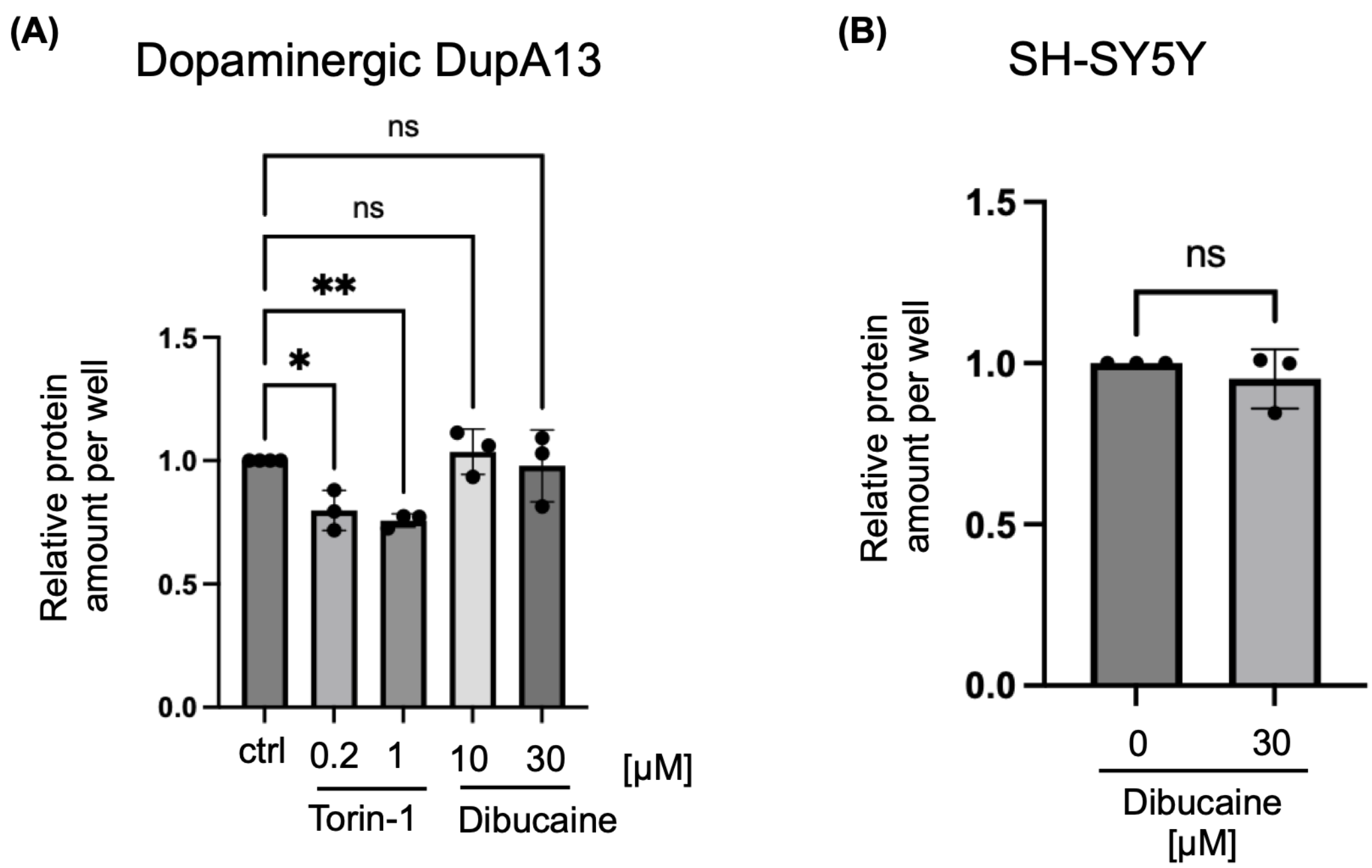

Supplementary Figure S4. Assessment of total protein content per well following dibucaine treatment in DupA13-derived dopaminergic neurons and SH-SY5Y cells

(A) Relative total protein amount per well in DupA13 dopaminergic neurons treated with control (ctrl; DMSO), Torin-1 (0.2 or 1 µM), or dibucaine (10 or 30 µM) for 5 days. Data represent the mean ± SD (n = 3-4 independent experiments). Statistical analysis was performed using one-way ANOVA followed by Dunnett's multiple comparisons test against the control. *$p < 0.05$, **$p < 0.01$; ns, not significant versus control.(B) Relative total protein amount per well in SH-SY5Y cells treated with 0 (control) or 30 µM dibucaine for 4 h. Data represent the mean ± SD (n = 3 independent experiments). Statistical significance was analyzed using Welch's t-test; ns, not significant ($p > 0.05$).

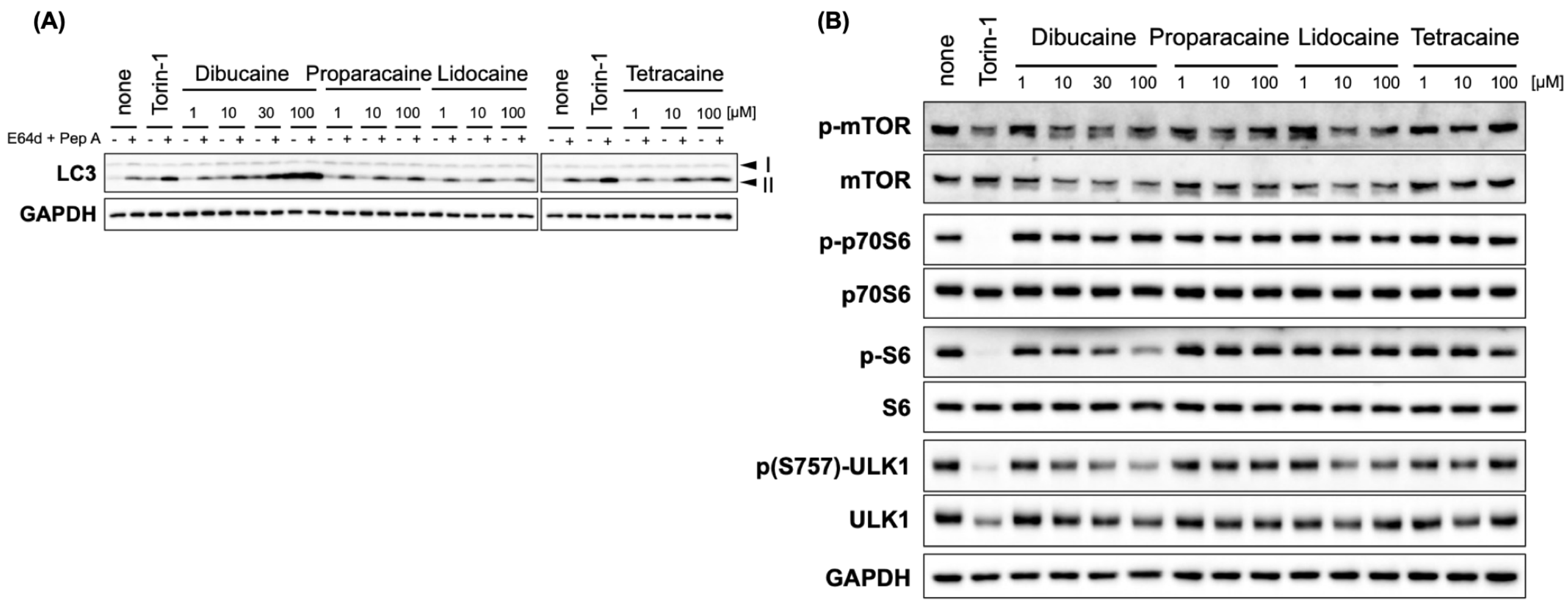

Supplementary Figure S5. Effects of dibucaine-related local anesthetics on autophagy induction in SH-SY5Y cells

SH-SY5Y cells were treated with local anesthetics structurally related to dibucaine for 4 hours (A) or 24 hours (B), and cell lysates were immunoblotted with the indicated antibodies. Western blot images corresponding to the quantitative data in Figure 4 are shown. Data are representative of two independent experiments (*n* = 1).

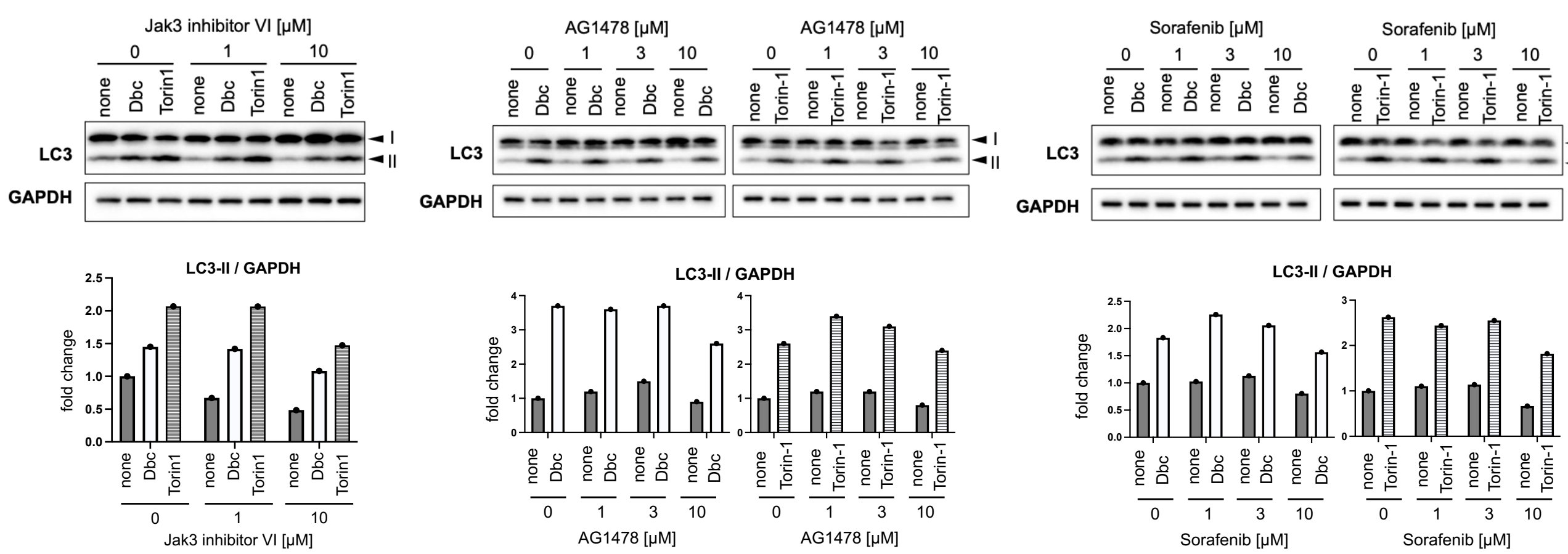

Supplementary Figure S6. Effects of protein kinase inhibitors on dibucaine-induced autophagy in SH-SY5Y cells

SH-SY5Y cells were pretreated with Janus kinase 3 (JAK3) inhibitor VI, AG1478, or sorafenib for 1 hour, followed by incubation with 30 µM dibucaine (Dbc) or 0.2 µM Torin-1 for an additional 4 hours. Cell lysates were then subjected to immunoblotting using antibodies against LC3 and GAPDH. Representative western blot images are shown. Quantitative data show relative LC3-II / GAPDH levels as a representative result from independent experiments (*n* = 1).

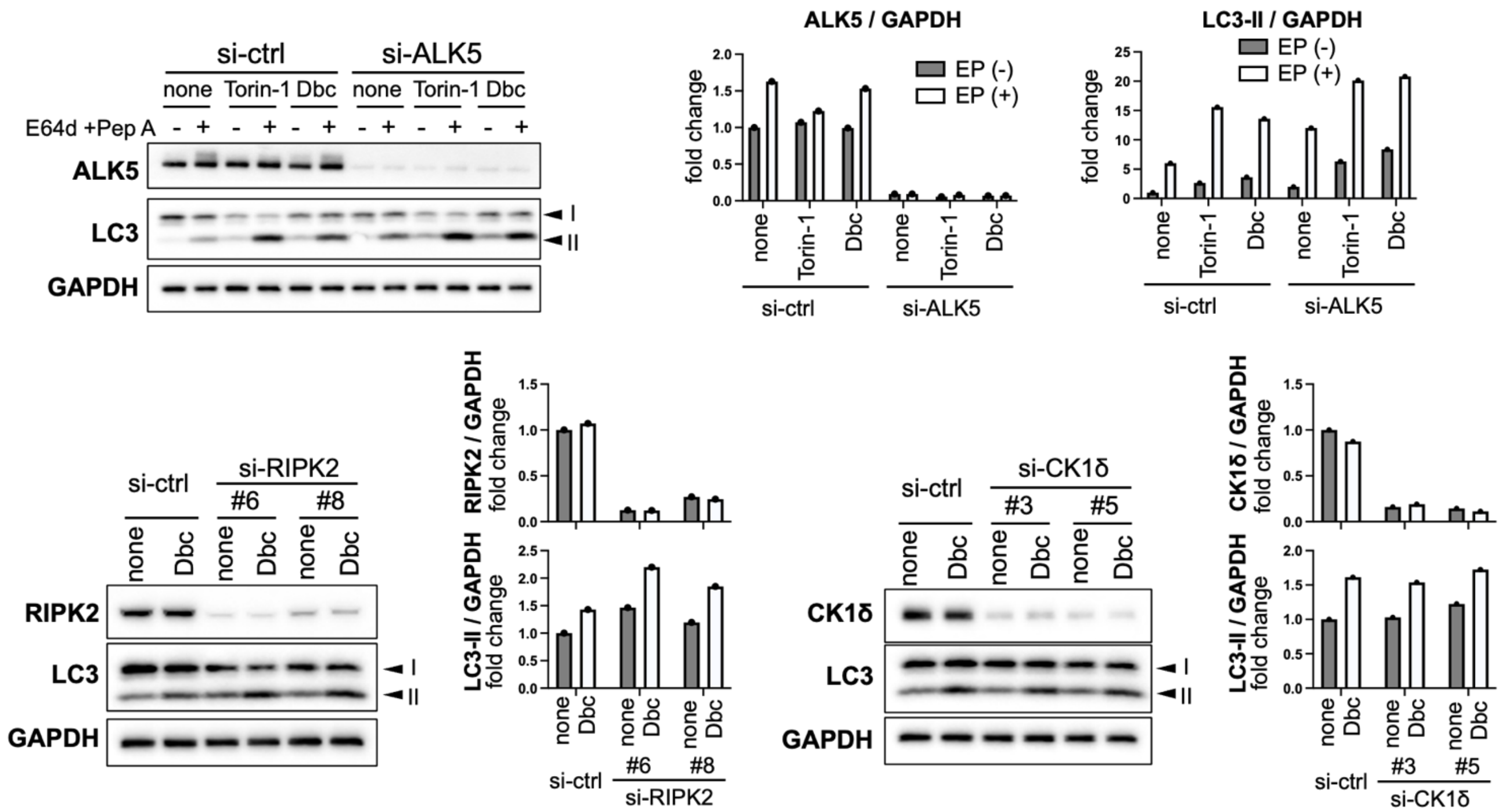

Supplementary Figure S7. Effects of protein kinase silencing on dibucaine-induced autophagy in SH-SY5Y cells

SH-SY5Y cells were transfected with siRNA targeting ALK5 (si-ALK5), RIPK2 (si-RIPK2 #6 and #8), CK1δ (si-CK1δ #3 and #5), or a non-targeting negative control (si-Control). The numbers (#6, #8, #3, and #5) denote distinct, independent siRNA constructs targeting different sequences of each gene. At 48 hours post-transfection, cells were treated with 30 μM dibucaine (Dbc) or 0.2 μM Torin-1 for an additional 4 hours, in the presence or absence of E64d plus Pepstatin A [EP(+/-)]. Cell lysates were then subjected to immunoblotting using indicated antibodies. Representative western blot images are shown. Quantitative data show relative protein levels from a representative experiment (*n* = 1; similar results were obtained in an independent experiment).

Supplementary

Blot Raw Data (uncropped)

Western Blot Source Data

for Figure 2A

LC3

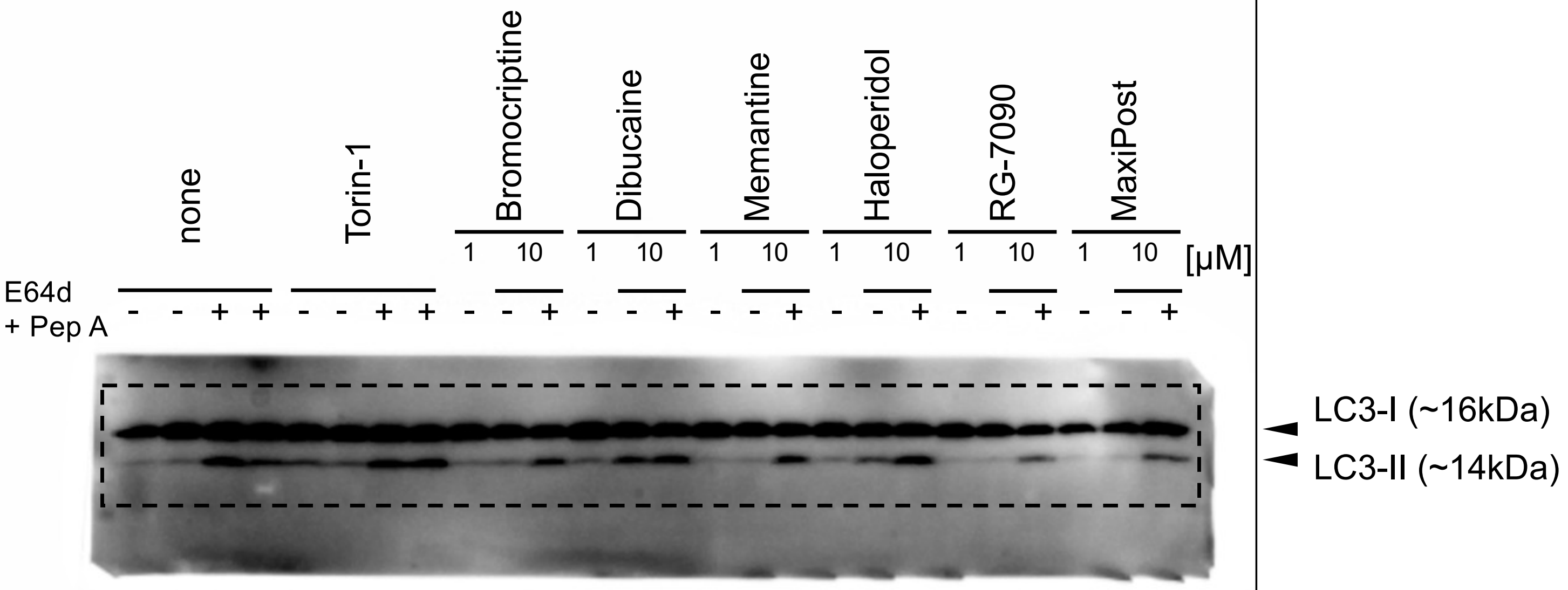

Uncropped original membrane image for Figure 2A. A molecular-weight marker lane was not captured or retained in the available image file. The expected molecular weights of LC3-I and LC3-II are approximately 16 kDa and 14 kDa, respectively. The expected molecular weights are provided for reference and do not represent molecular-weight markers measured in this image.

Western Blot Source Data for Figure 2A

GAPDH

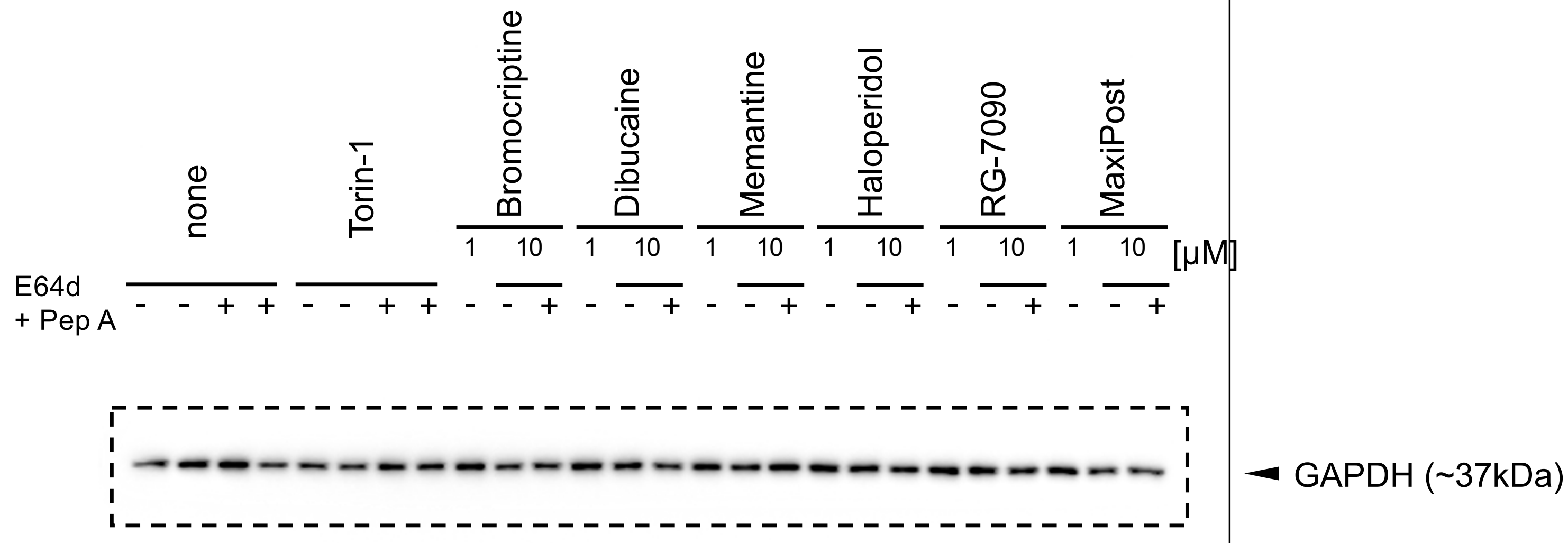

The original uncropped blot is shown. A molecular-weight marker lane was not captured or retained in the available image file. The expected molecular weight of GAPDH is approximately 37 kDa. The expected molecular weight is provided for reference and does not represent a molecular-weight marker measured in this image.

Western Blot Source Data for Figure 3B

LC3

Uncropped source data for Figure 3B (LC3). Left: Brightfield image of the membrane with molecular markers. Right: Corresponding chemiluminescence image. Dotted box indicates the region displayed in Figure 3B.

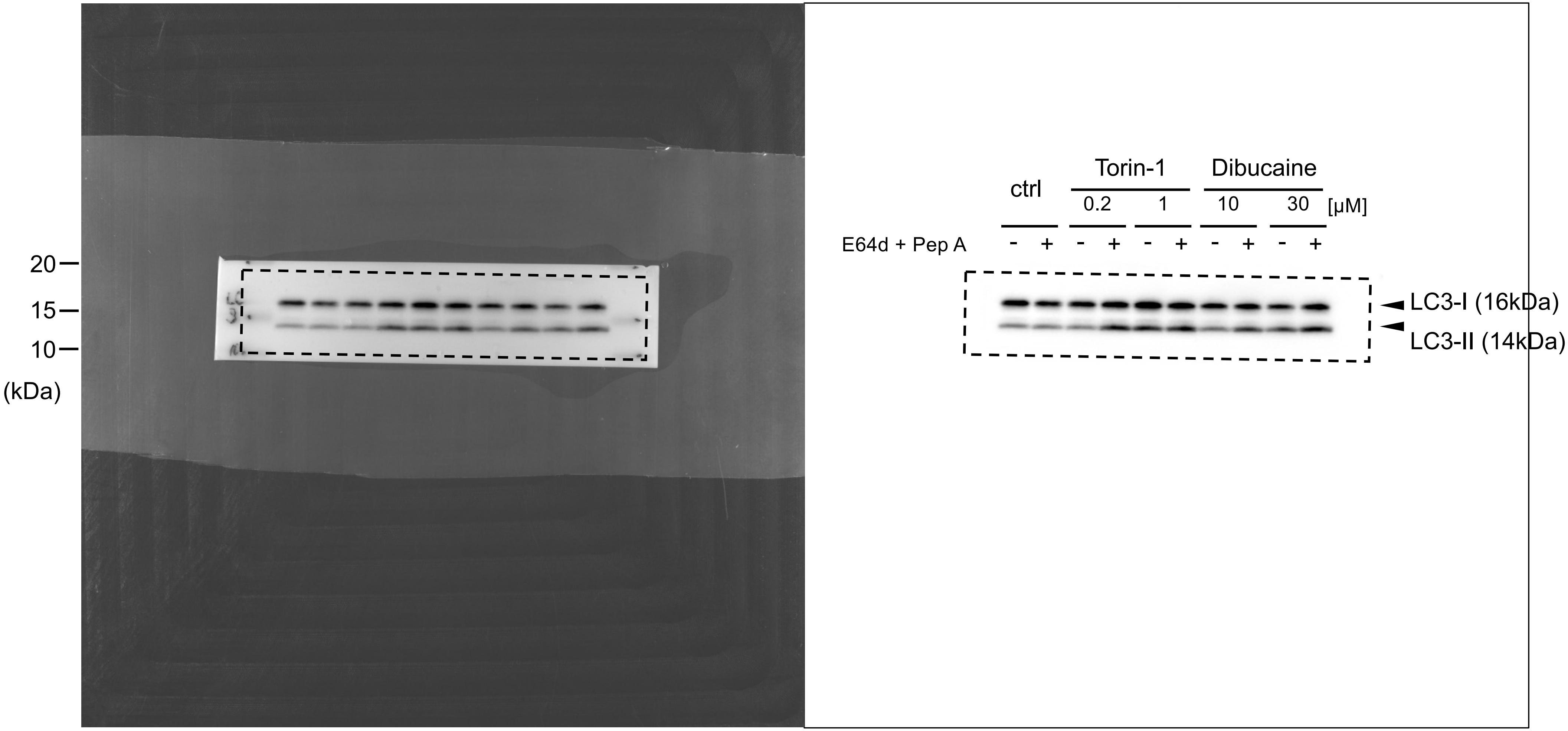

Western Blot Source Data for Figure 3B

TH

Uncropped source data for Figure 3B (TH). Left: Brightfield image of the membrane with molecular markers. Right: Corresponding chemiluminescence image. Dotted box indicates the region displayed in Figure 3B.

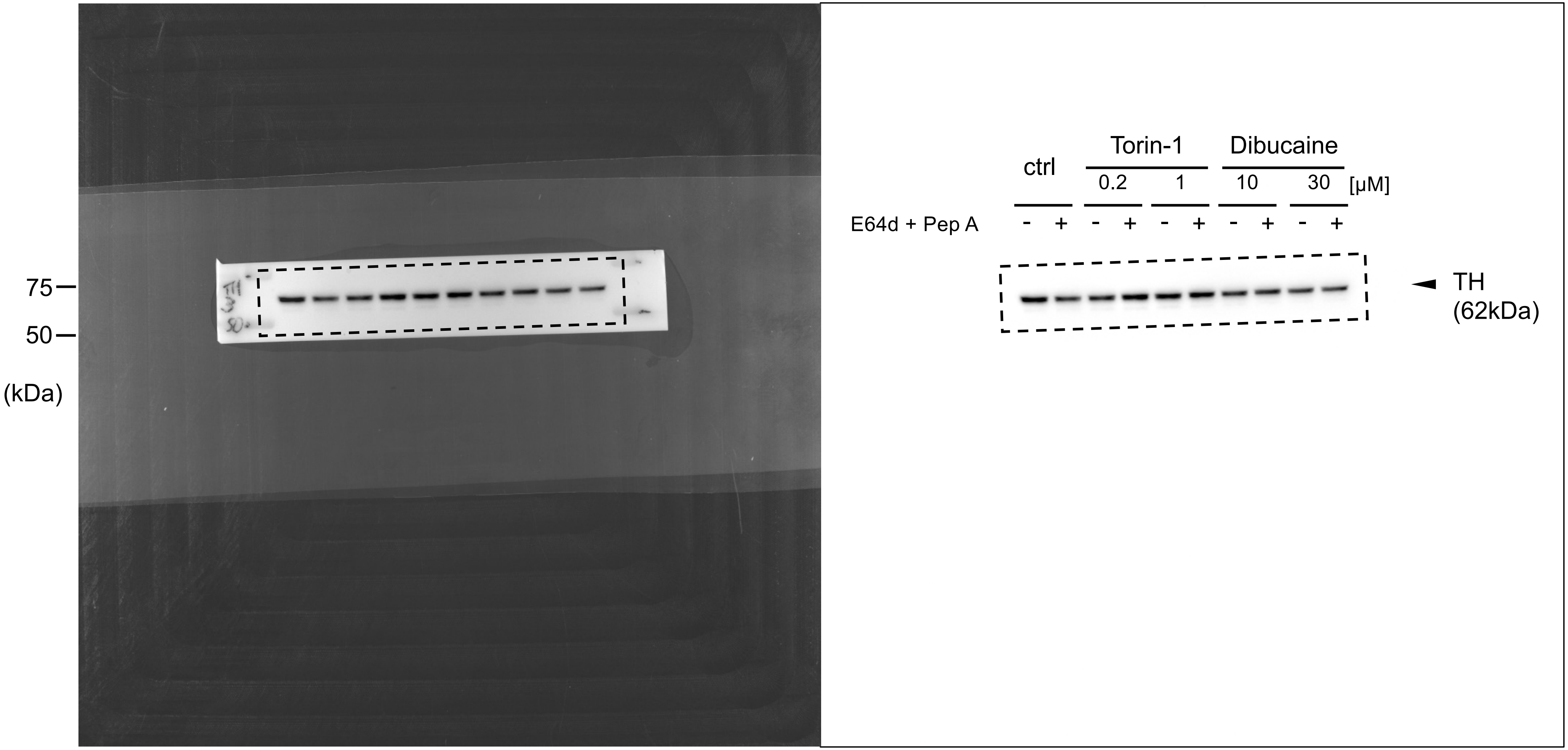

Western Blot Source Data for Figure 3B
GAPDH

Uncropped source data for Figure 3B (GAPDH). Left: Brightfield image of the membrane with molecular markers. Right: Corresponding chemiluminescence image. Dotted box indicates the region displayed in Figure 3B.

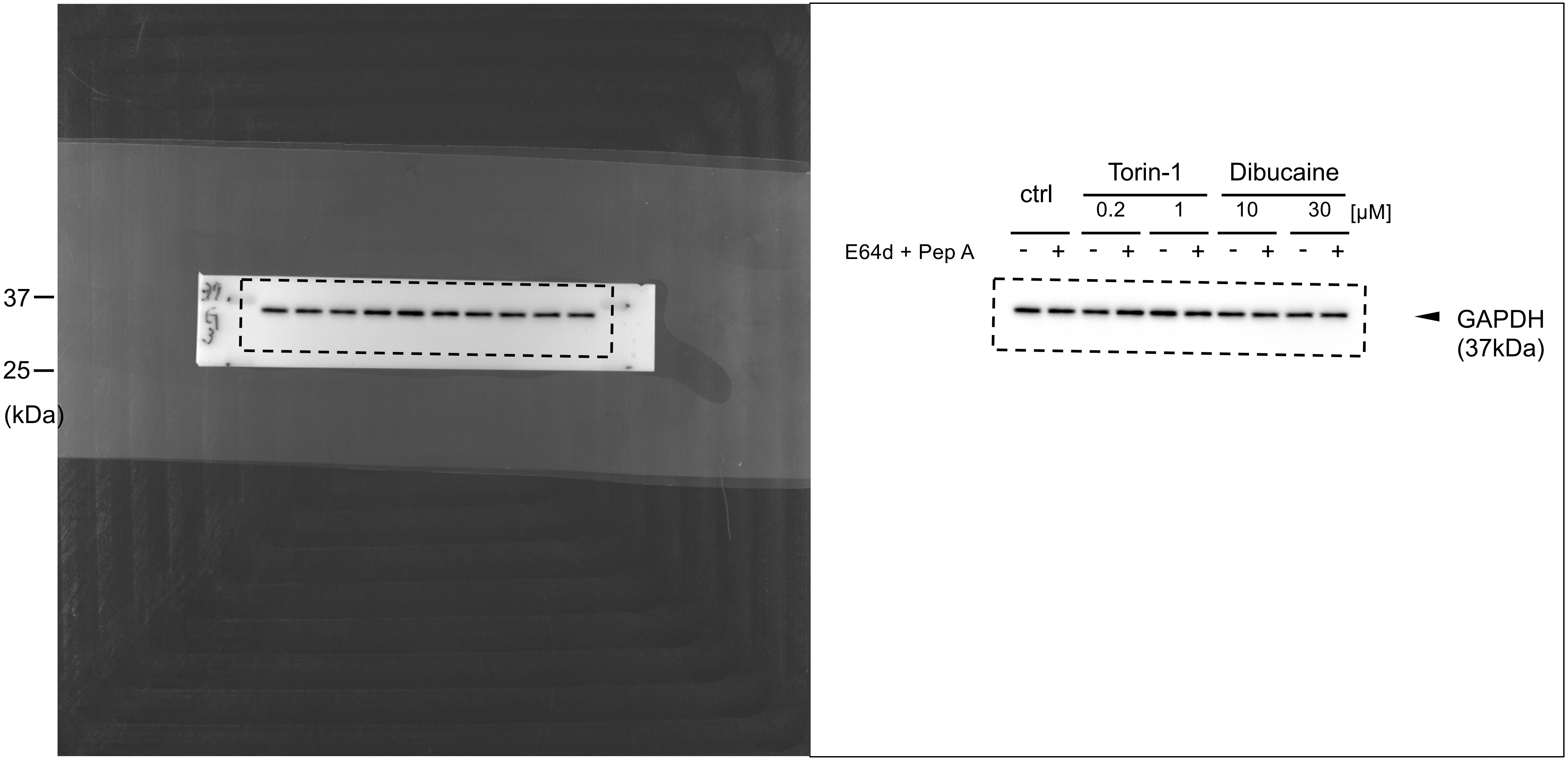

Western Blot Source Data for Figure 3C
p-αSyn

Uncropped source data for Figure 3C (p-αSyn). Left: Brightfield image of the membrane with molecular markers. Right: Corresponding chemiluminescence image. Lanes outside the dotted box contain unrelated samples. Dotted box indicates the region displayed in Figure 3C.

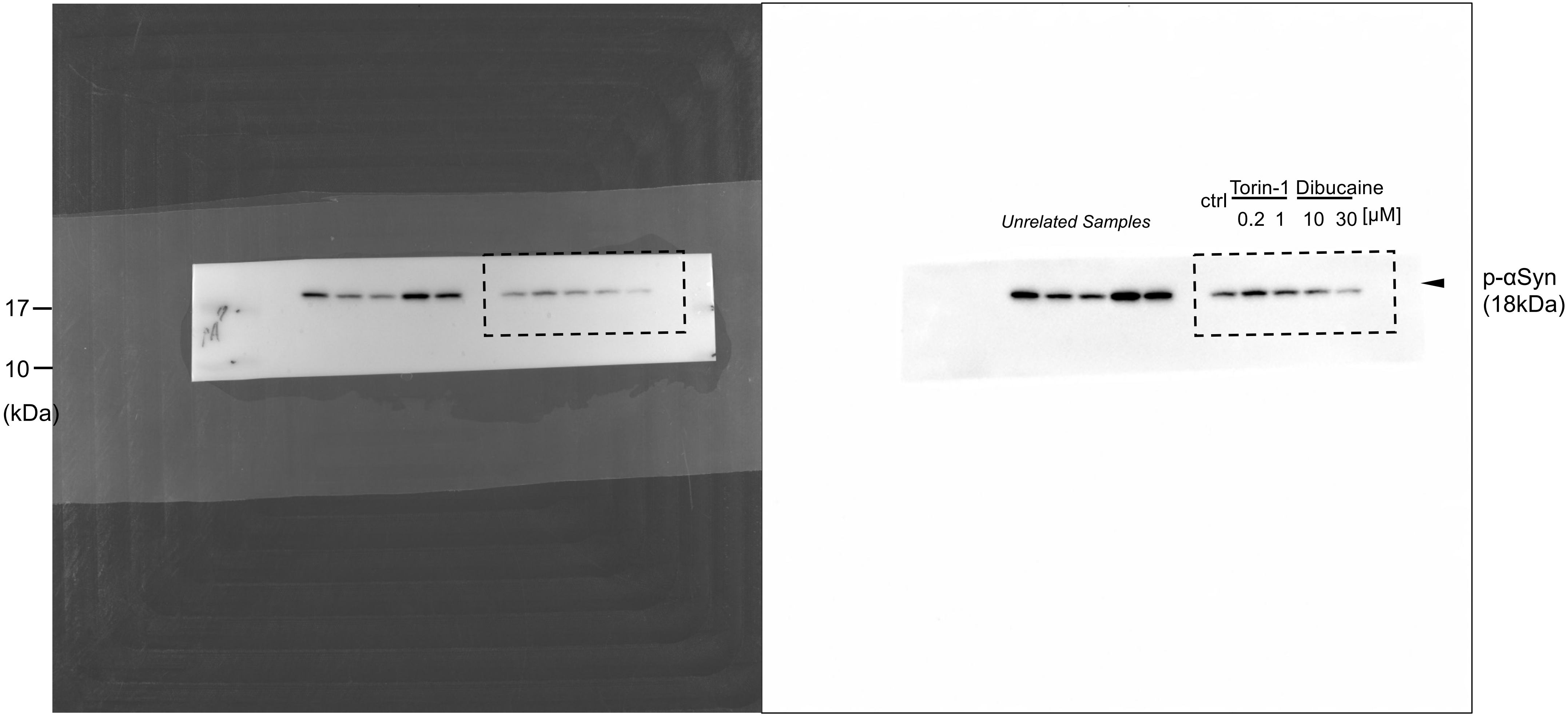

Western Blot Source Data for Figure 3C

αSyn

Uncropped source data for Figure 3C (αSyn). Left: Brightfield image of the membrane with molecular markers. Right: Corresponding chemiluminescence image. Lanes outside the dotted box contain unrelated samples. Dotted box indicates the region displayed in Figure 3C.

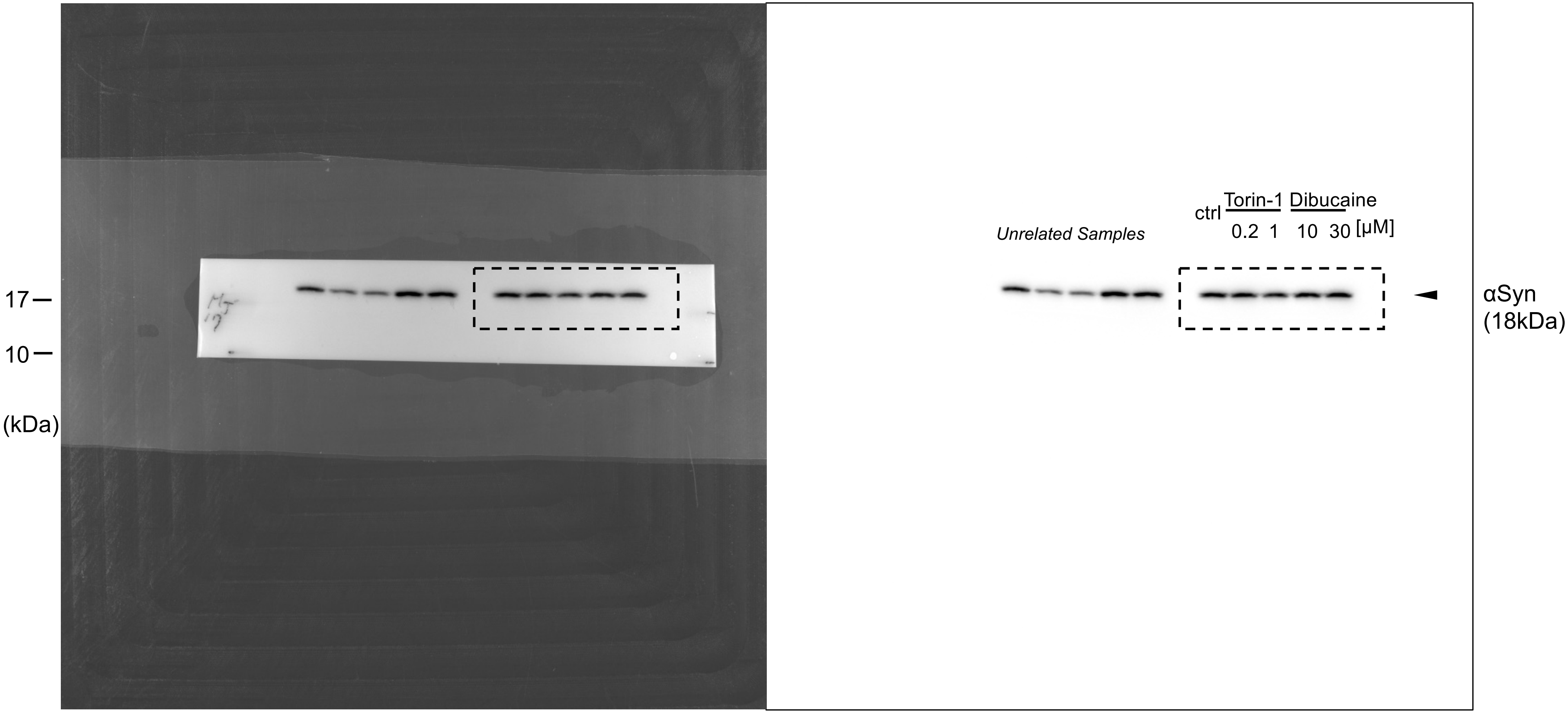

Western Blot Source Data for Figure 3C
TH

Uncropped source data for Figure 3C (TH). Left: Brightfield image of the membrane with molecular markers. Right: Corresponding chemiluminescence image. Lanes outside the dotted box contain unrelated samples. Dotted box indicates the region displayed in Figure 3C.

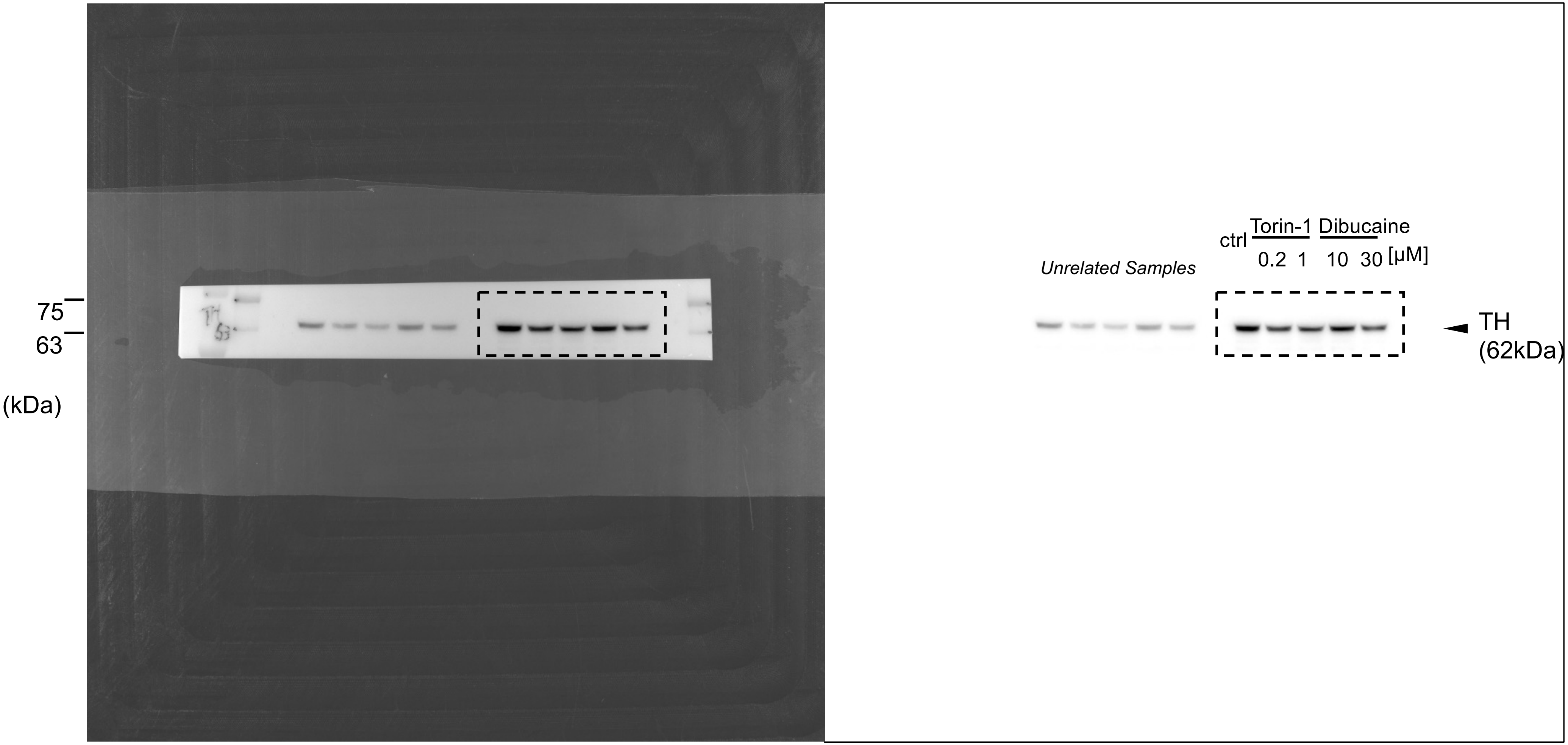

Western Blot Source Data for Figure 3C

GAPDH

Uncropped source data for Figure 3C (GAPDH). Left: Brightfield image of the membrane with molecular markers. Right: Corresponding chemiluminescence image. Lanes outside the dotted box contain unrelated samples. Dotted box indicates the region displayed in Figure 3C.

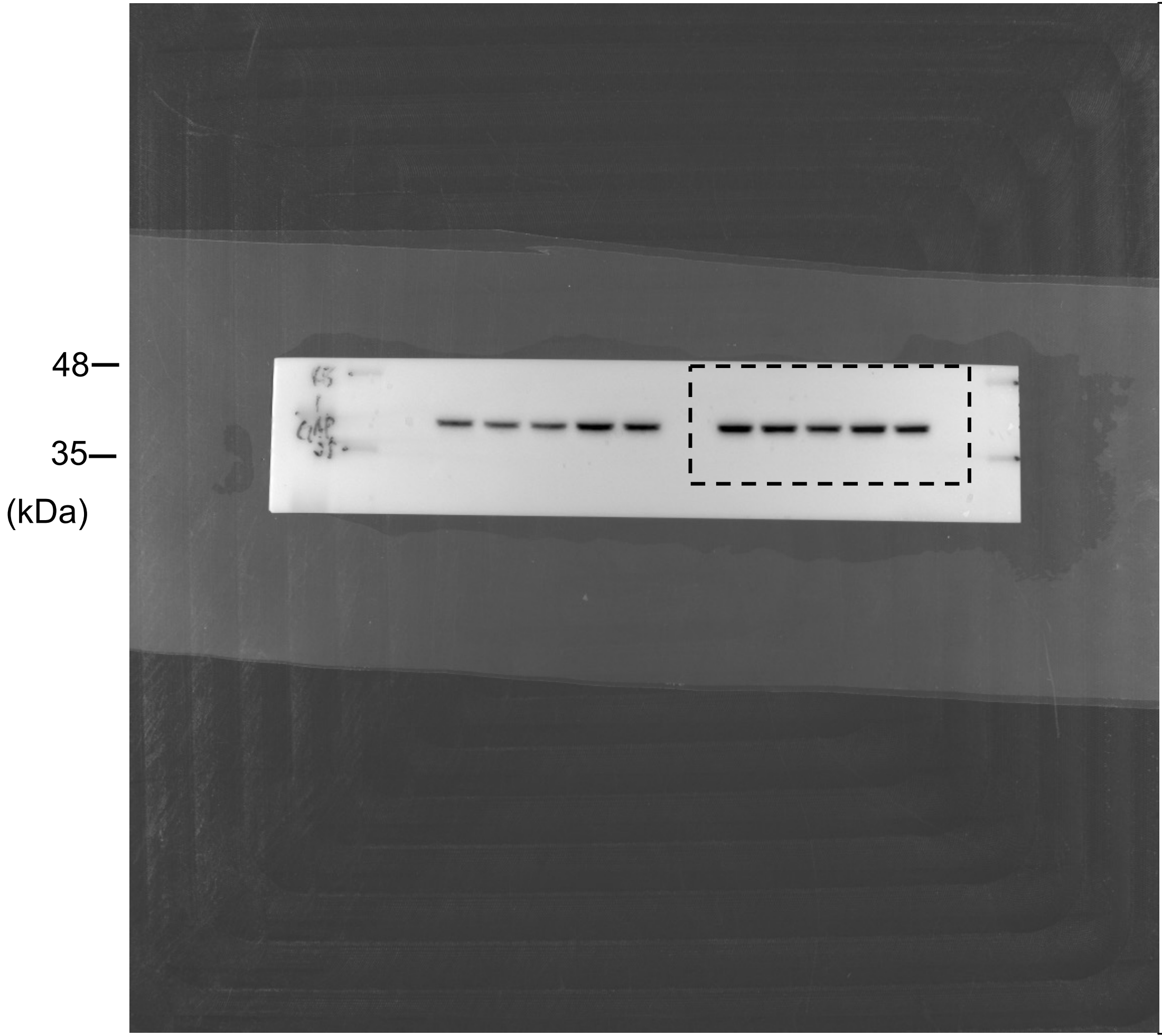

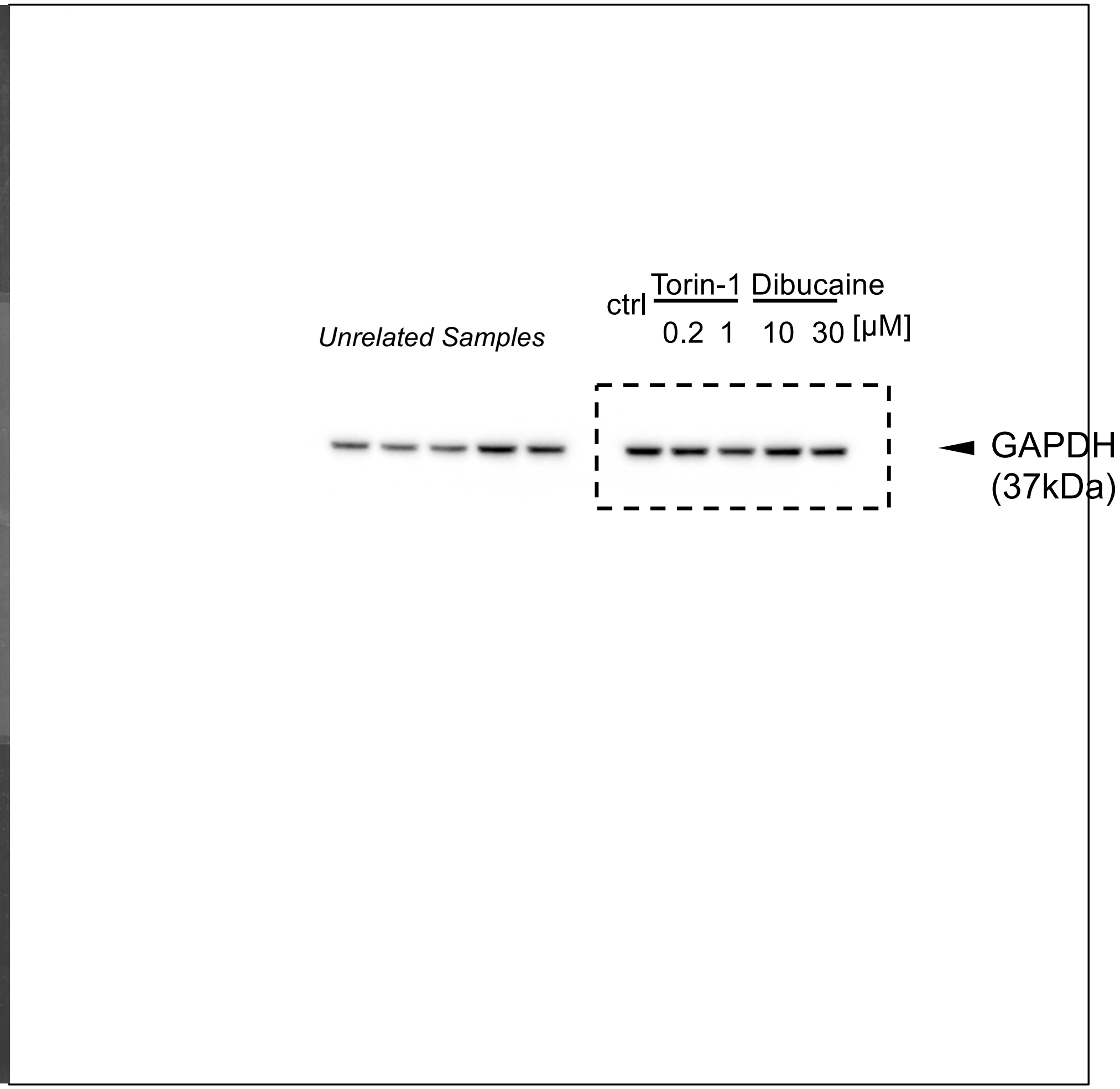

Western Blot Source Data for Figure 5B

LC3

Uncropped source data for Figure 5B (LC3). Left: Brightfield image of the membrane with molecular markers. Right: Corresponding chemiluminescence image. Upper membrane shows biological replicates. Lanes outside the dotted boxes represent extra or unrelated samples. Dotted boxes indicate the region displayed in Figure 5B.

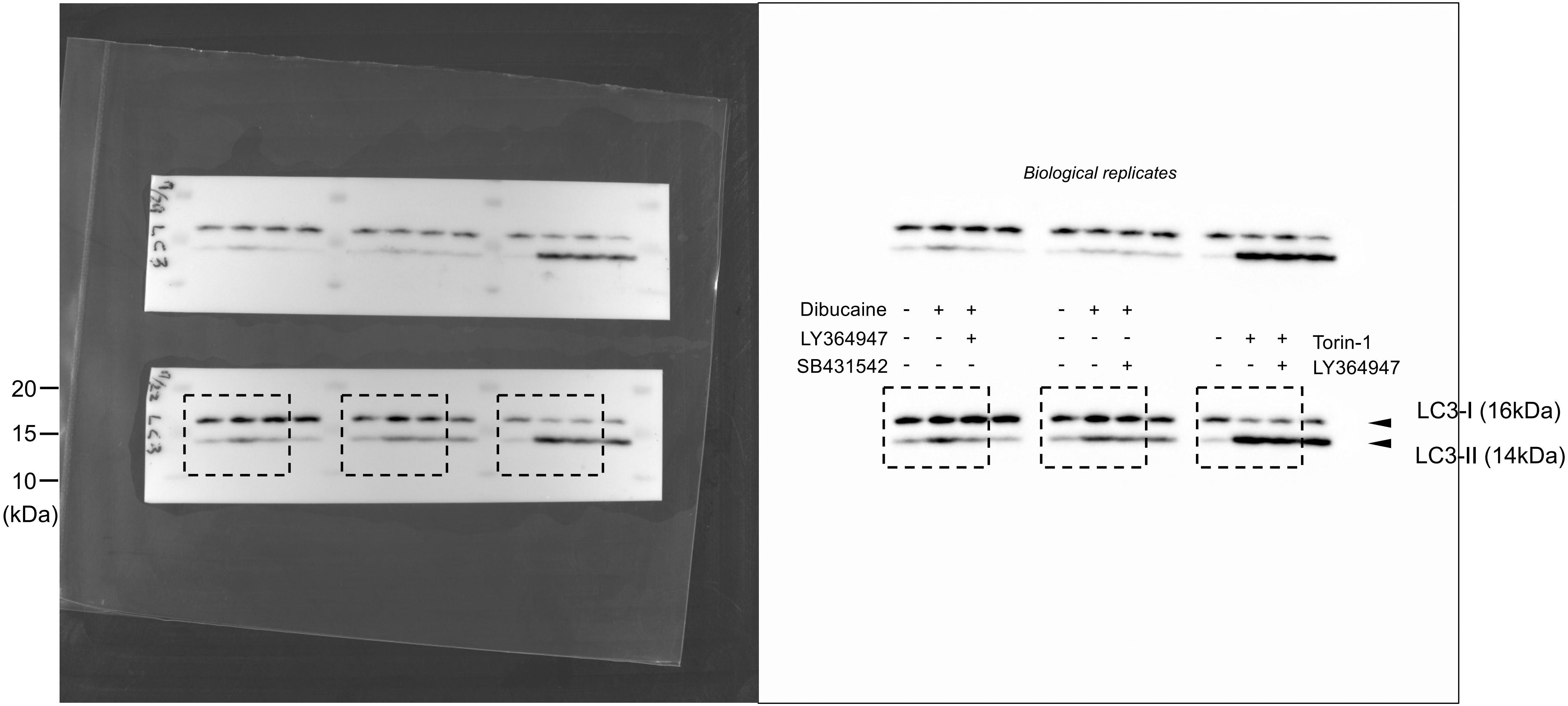

Western Blot Source Data for Figure 5B
GAPDH

Uncropped source data for Figure 5B (GAPDH). Left: Brightfield image of the membrane with molecular markers. Right: Corresponding chemiluminescence image (original exposure). Upper membrane shows biological replicates. Lanes outside the dotted boxes represent extra or unrelated samples. Dotted boxes indicate the region displayed in Figure 5B. Contrast-adjusted image of Torin-1/LY364947 samples for clearer visualization is shown next.

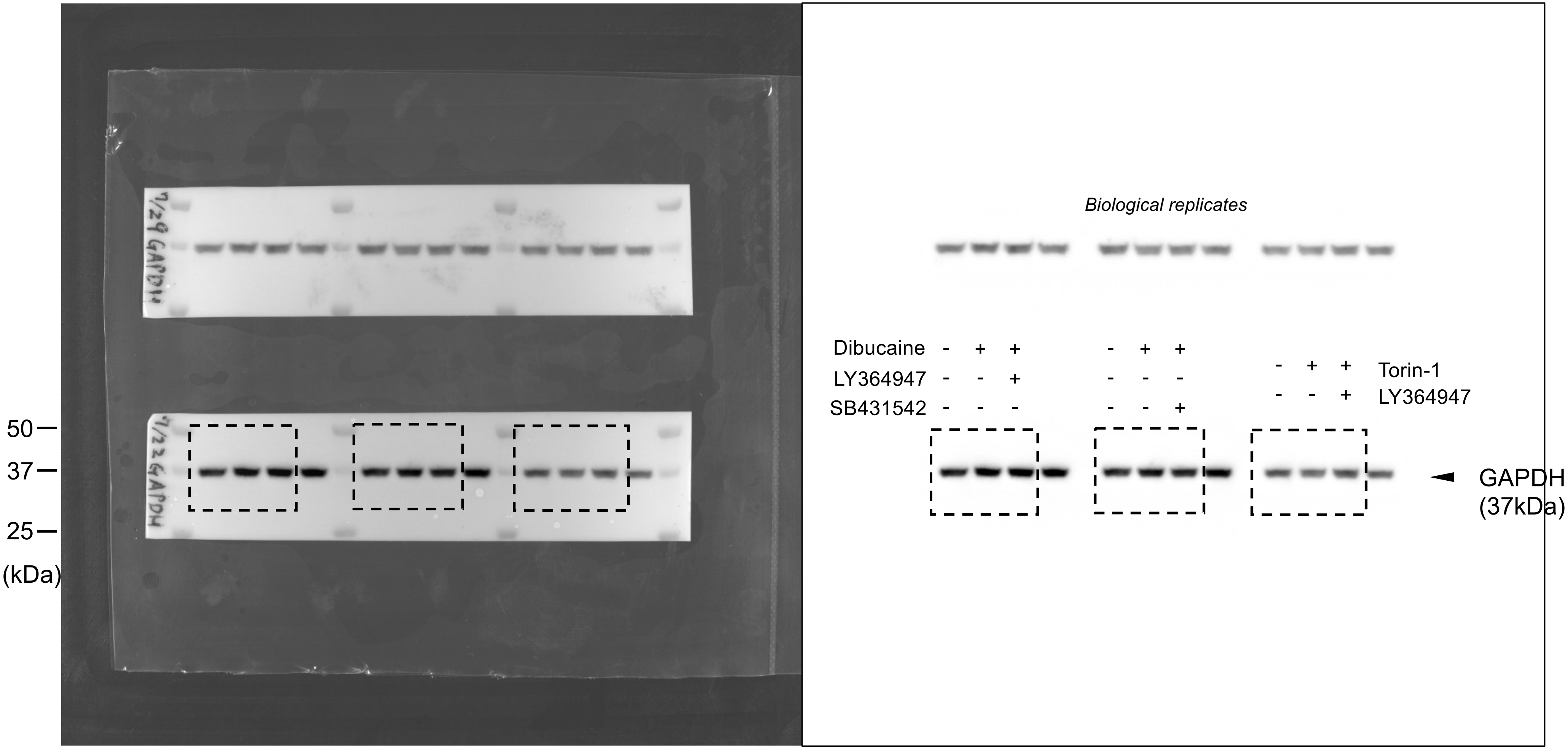

Western Blot Source Data for Figure 5B

GAPDH

Uncropped source data for Figure 5B (GAPDH; high-contrast/brightness-adjusted image for better visualization of the right lanes). Left: Brightfield image of the membrane. Right: Corresponding chemiluminescence image. Upper membrane shows biological replicates. Lanes outside the dotted box represent extra or unrelated samples. Dotted boxes indicate the region displayed in Figure 5B.

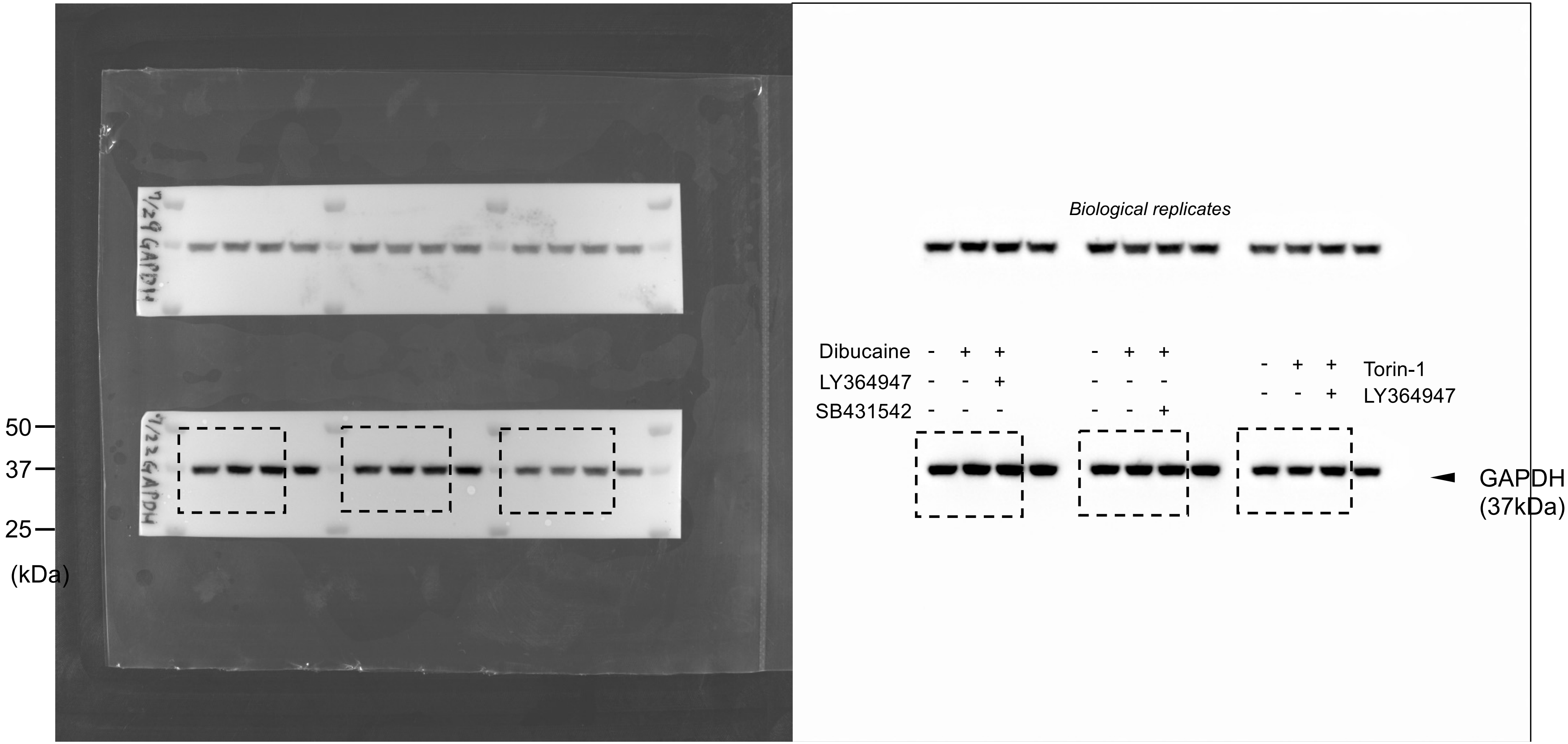

Western Blot Source Data for Figure 5C

LC3

Uncropped source data for Figure 5C (LC3). Left: Brightfield image of the membrane with molecular markers. Right: Corresponding chemiluminescence image. Dotted boxes indicate the region displayed in Figure 5C.

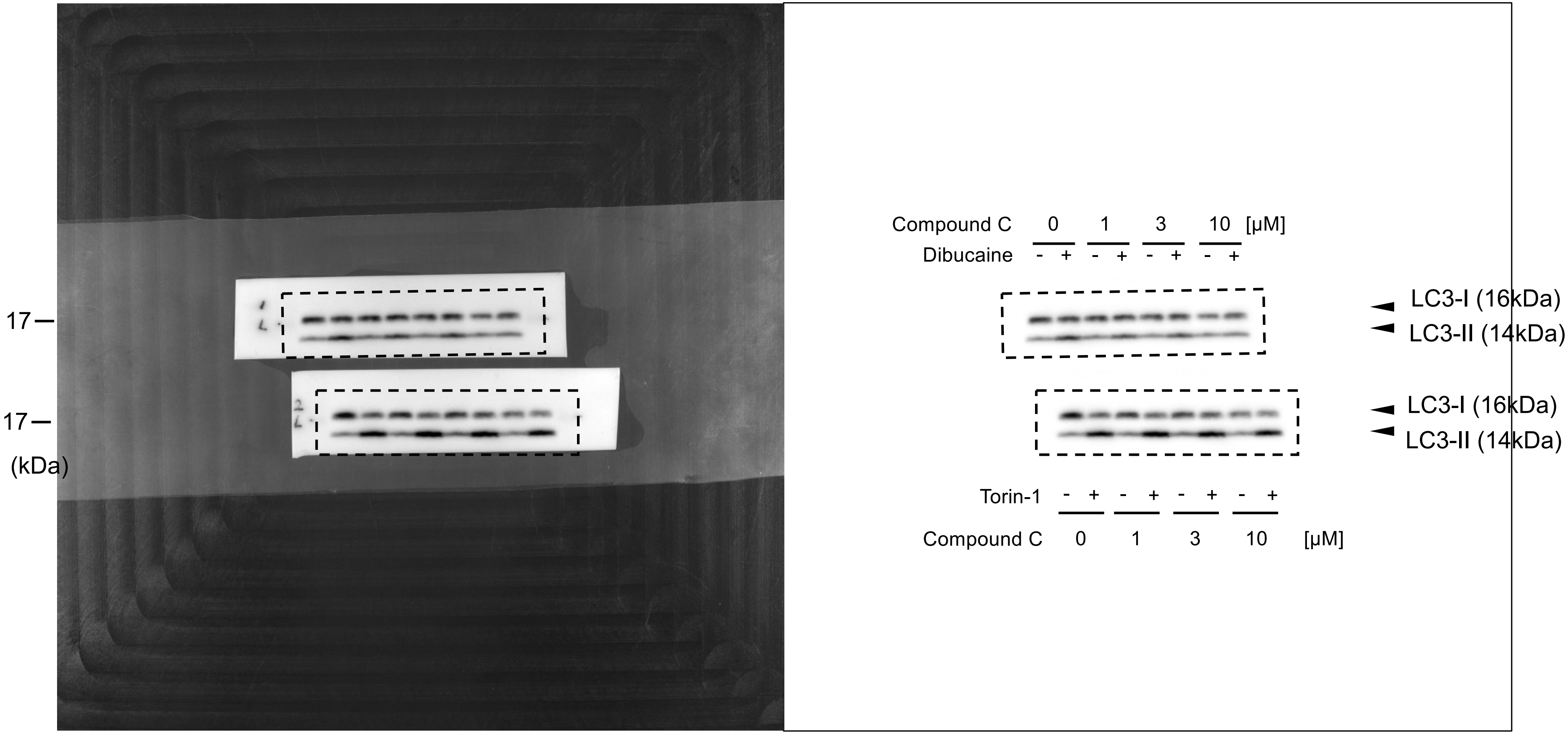

Western Blot Source Data for Figure 5C

GAPDH

Uncropped source data for Figure 5C (GAPDH). Left: Brightfield image of the membrane with molecular markers. Right: Corresponding chemiluminescence image. Dotted boxes indicate the region displayed in Figure 5C.

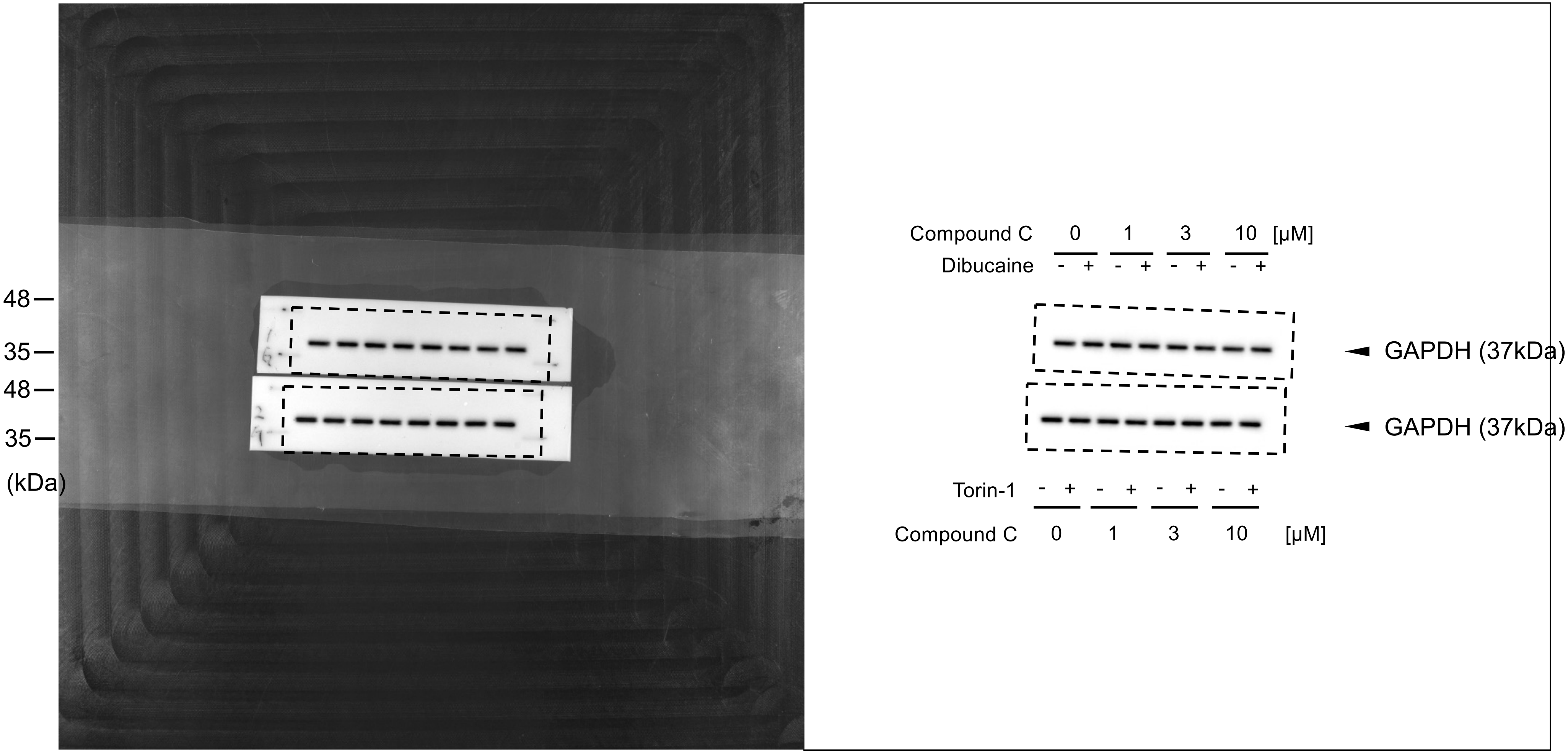

Western Blot Source Data for Figure 5D

pAMPKα

Uncropped source data for Figure 5D (pAMPKα). Left: Brightfield image of the membrane with molecular markers. Right: Corresponding chemiluminescence image. Lanes outside the dotted box contain unrelated samples. Dotted box indicates the region displayed in Figure 5D.

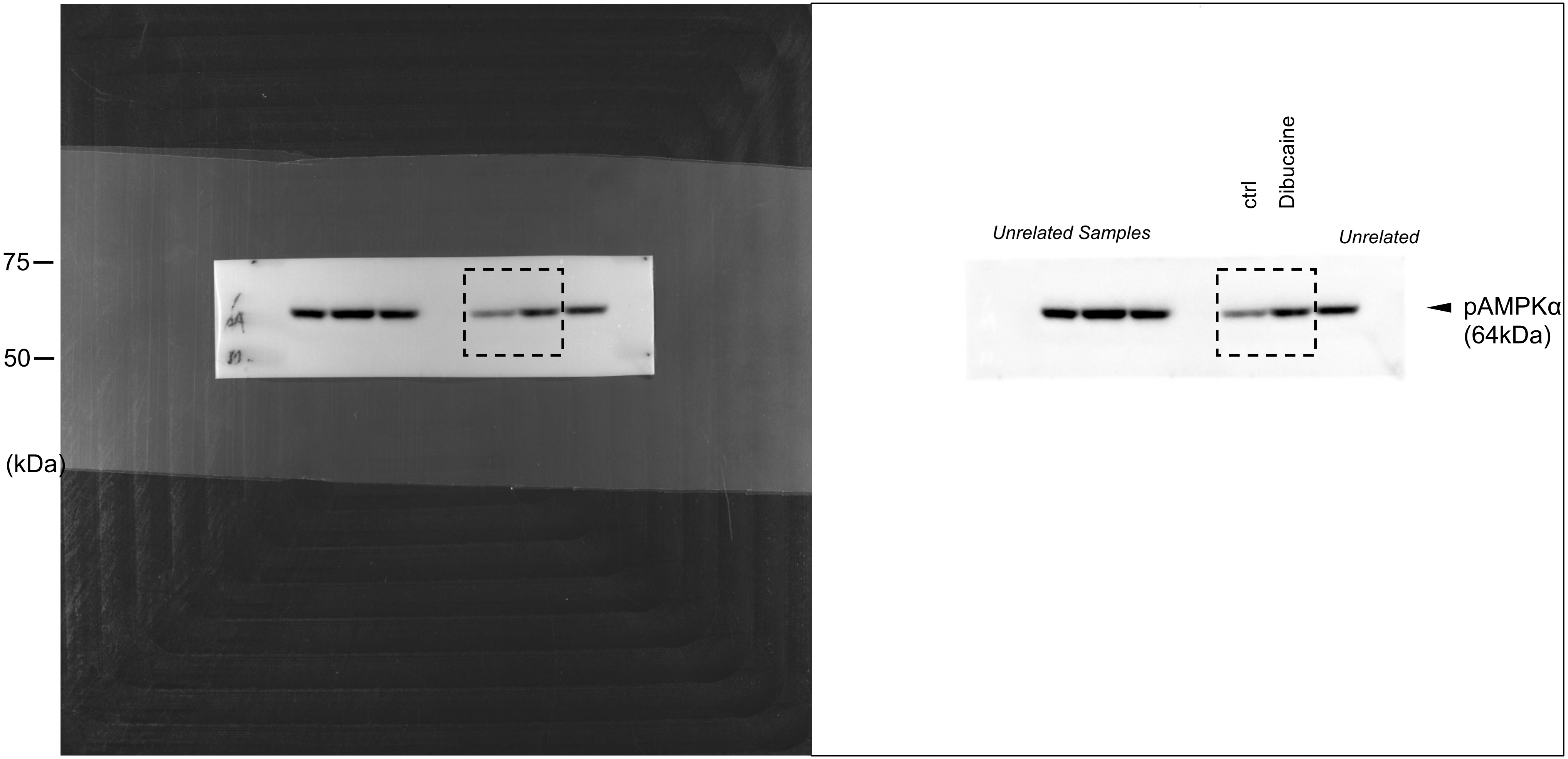

Western Blot Source Data for Figure 5D

AMPKα

Uncropped source data for Figure 5D (AMPKα). Left: Brightfield image of the membrane with molecular markers. Right: Corresponding chemiluminescence image. Lanes outside the dotted box contain unrelated samples. Dotted box indicates the region displayed in Figure 5D.

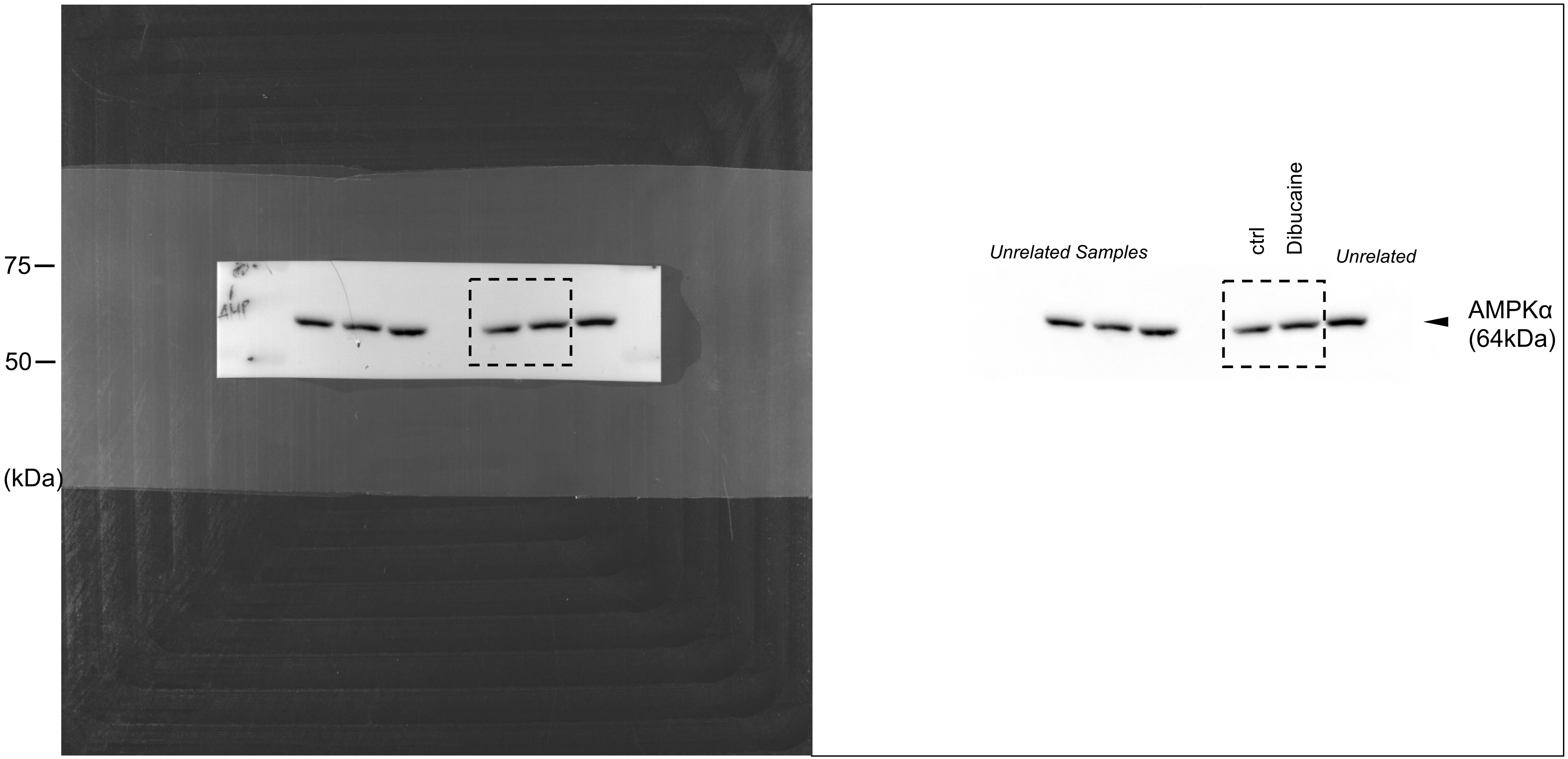

Western Blot Source Data for Figure 5D

αTubulin

Uncropped source data for Figure 5D (αTubulin). Left: Brightfield image of the membrane with molecular markers. Right: Corresponding chemiluminescence image. Lanes outside the dotted box contain unrelated samples. Dotted box indicates the region displayed in Figure 5D.

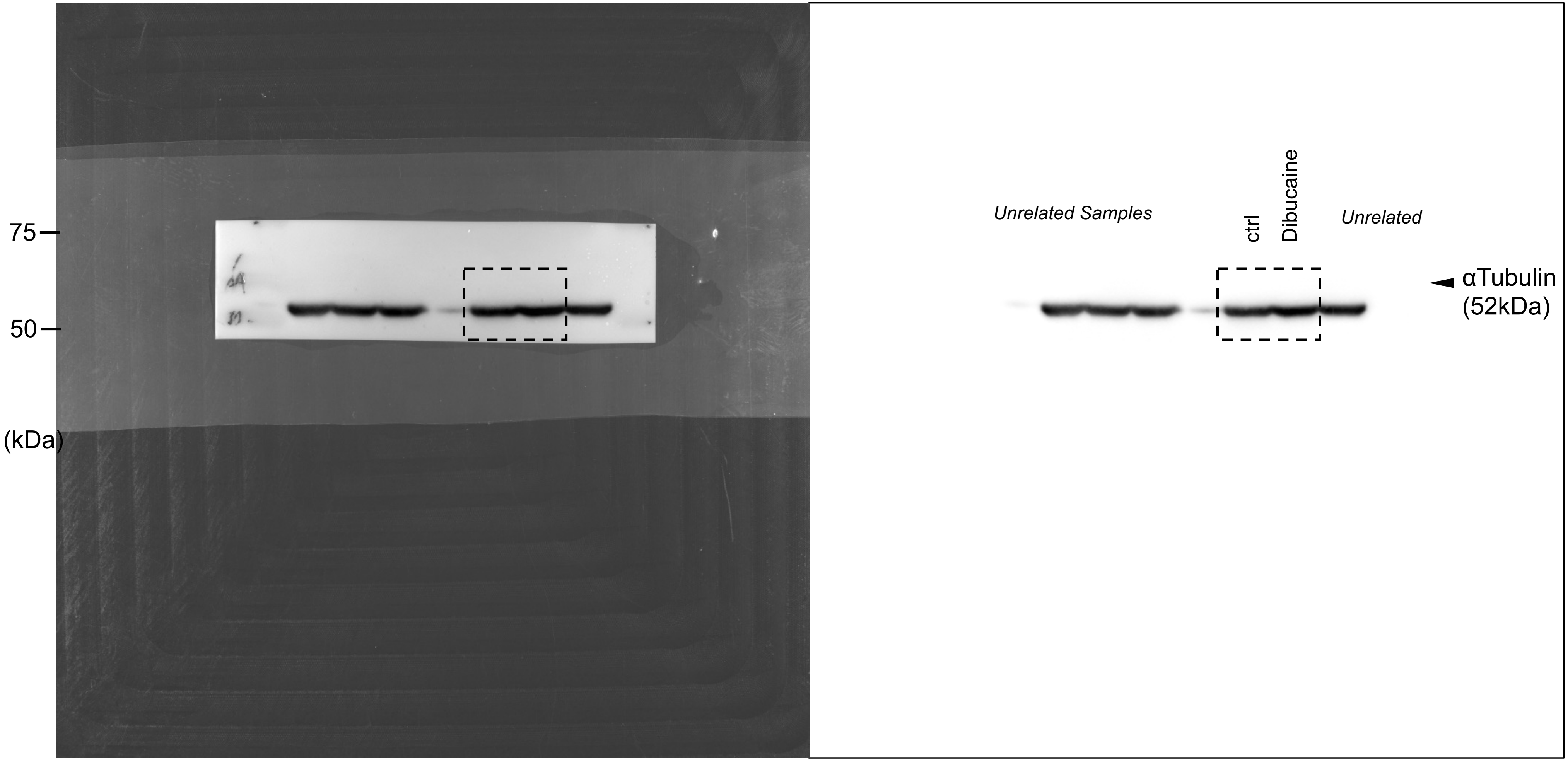

Western Blot Source Data for Figure 5E

pAMPKα

Uncropped source data for Figure 5E (pAMPKα). Left: Brightfield image of the membrane with molecular markers. Right: Corresponding chemiluminescence image. Lanes outside the dotted box contain biological replicates. Dotted box indicates the region displayed in Figure 5E.

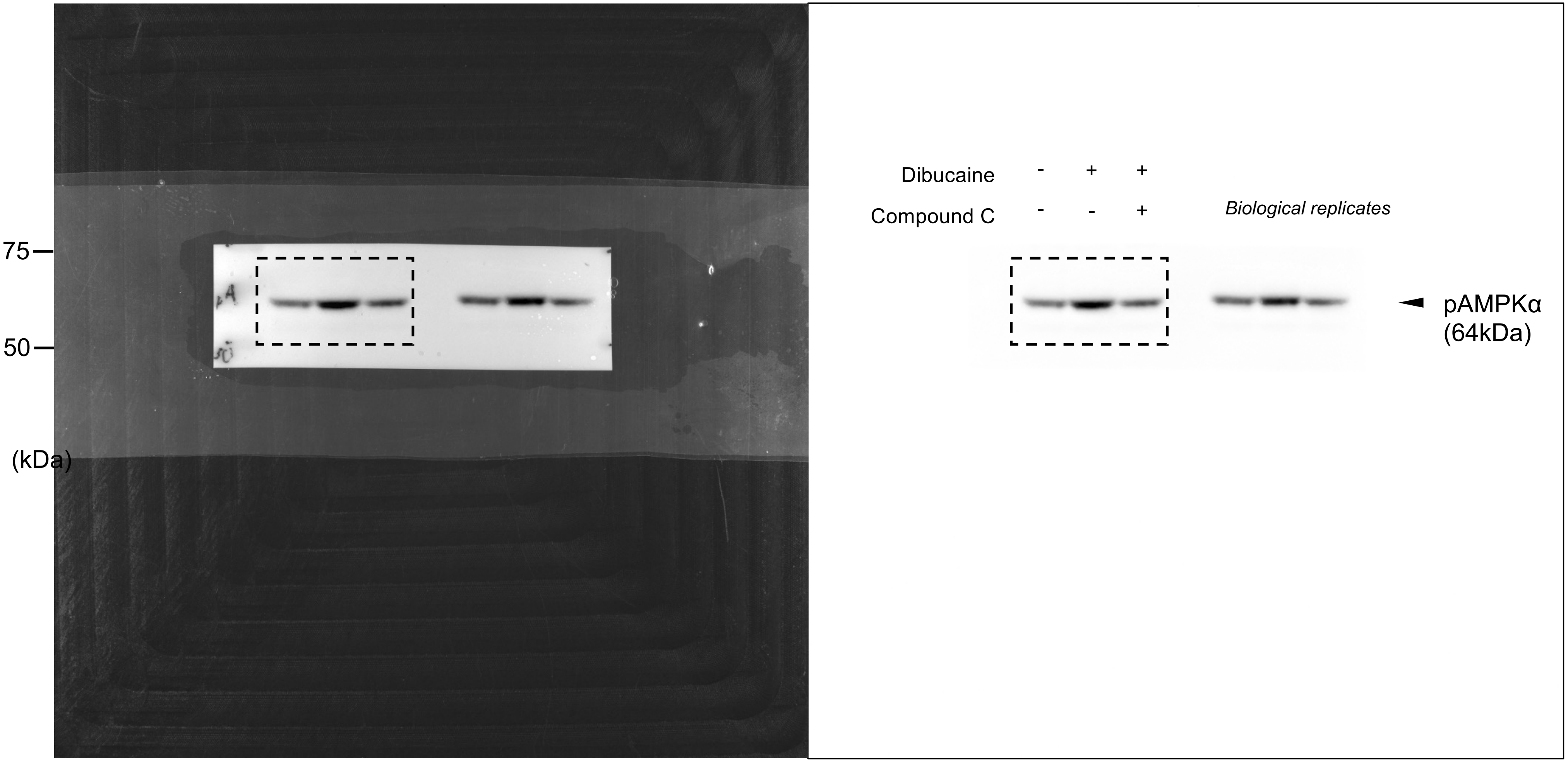

Western Blot Source Data for Figure 5E

AMPKα

Uncropped source data for Figure 5E (AMPKα). Left: Brightfield image of the membrane with molecular markers. Right: Corresponding chemiluminescence image. Lanes outside the dotted box contain biological replicates. Dotted box indicates the region displayed in Figure 5E.

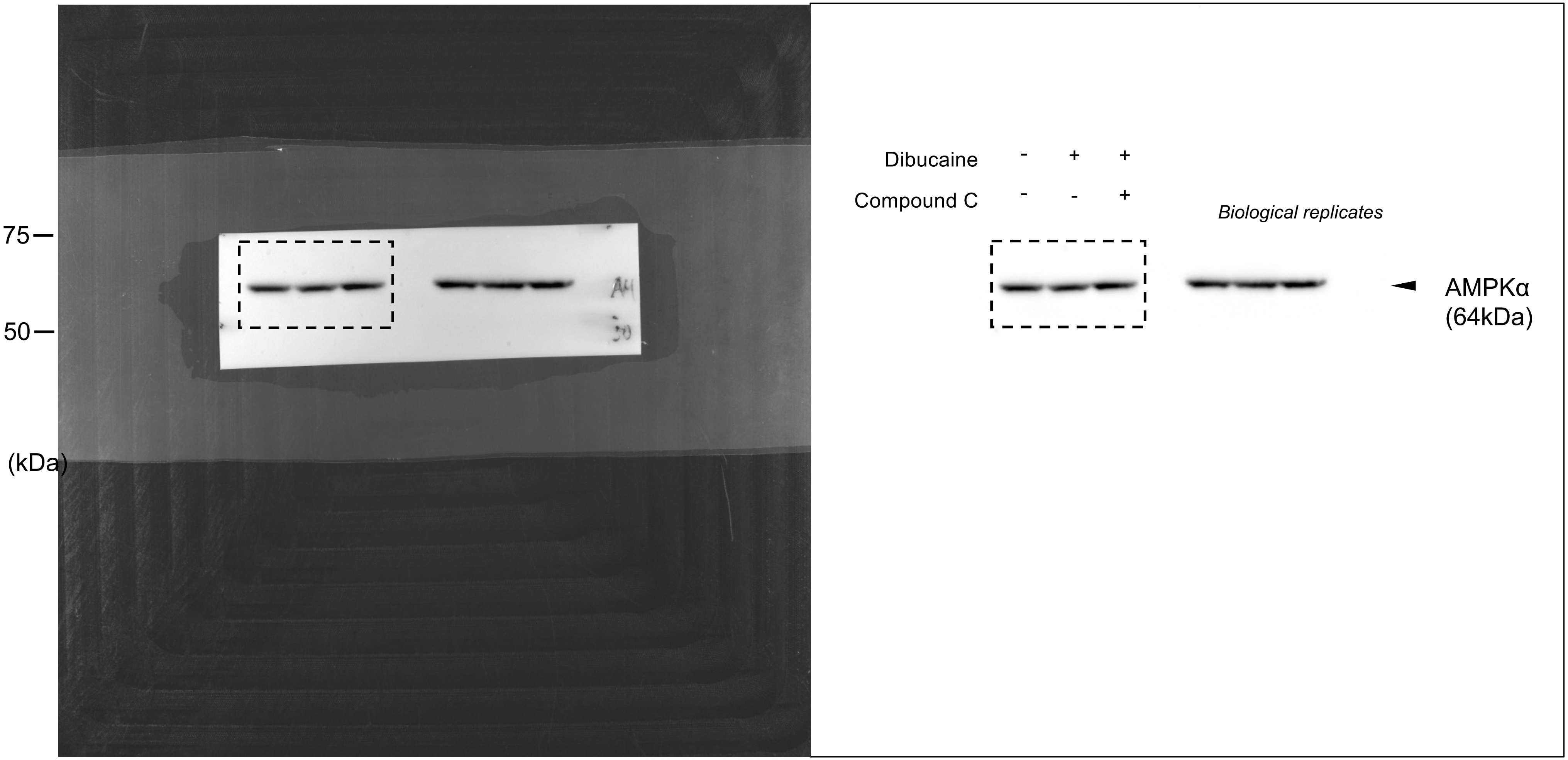

Western Blot Source Data for Figure 5E

αTubulin

Uncropped source data for Figure 5E (αTubulin). Left: Brightfield image of the membrane with molecular markers. Right: Corresponding chemiluminescence image. Lanes outside the dotted box contain biological replicates. Dotted box indicates the region displayed in Figure 5E.

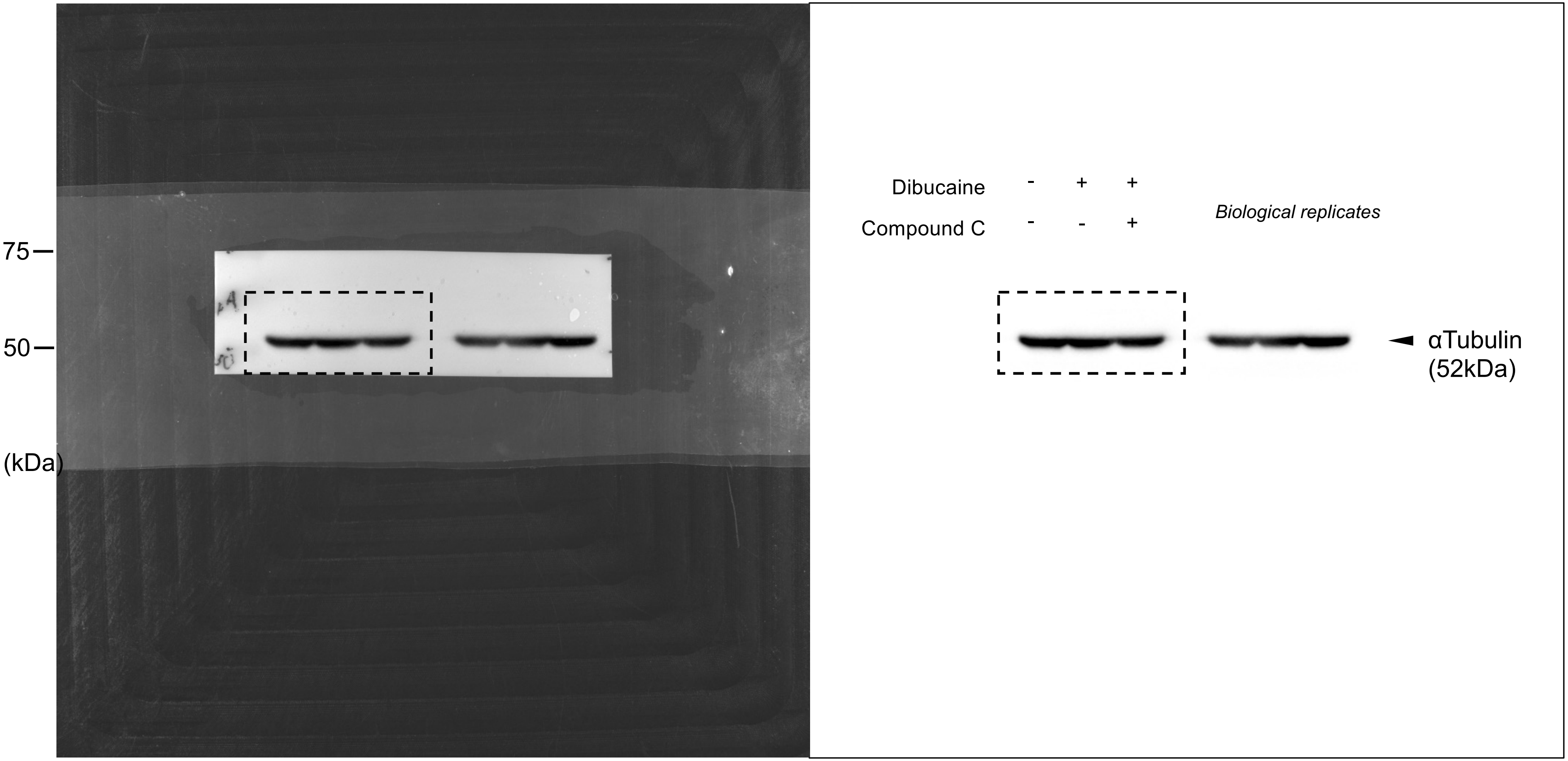

Western Blot Source Data for Figure 5F

pAMPKα

Uncropped source data for Figure 5F (pAMPKα). Left: Brightfield image of the membrane with molecular markers. Right: Corresponding chemiluminescence image. Lanes outside the dotted box contain biological replicates. Dotted box indicates the region displayed in Figure 5F.

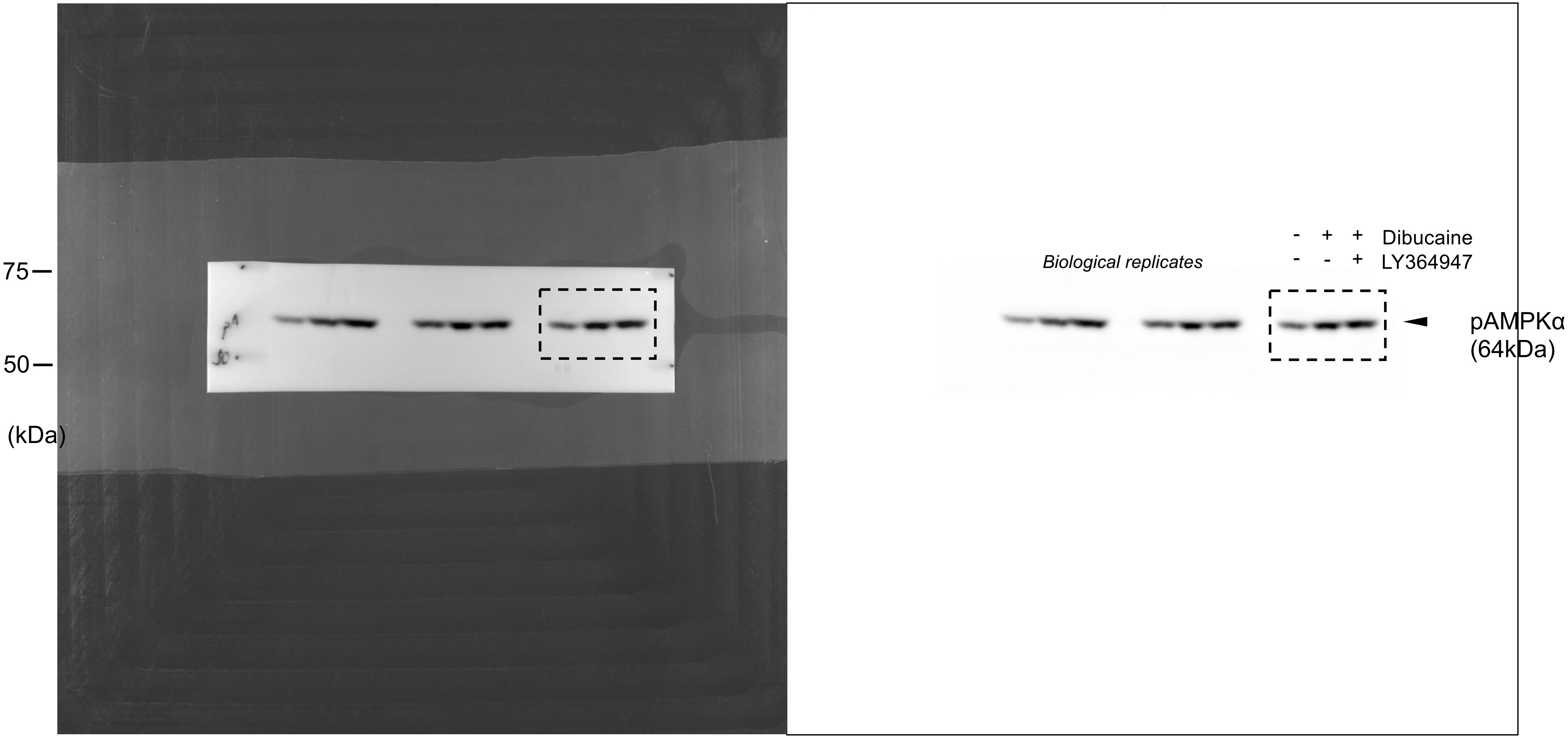

Western Blot Source Data for Figure 5F

AMPKα

Uncropped source data for Figure 5F (AMPKα). Left: Brightfield image of the membrane with molecular markers. Right: Corresponding chemiluminescence image. Lanes outside the dotted box contain biological replicates. Dotted box indicates the region displayed in Figure 5F.

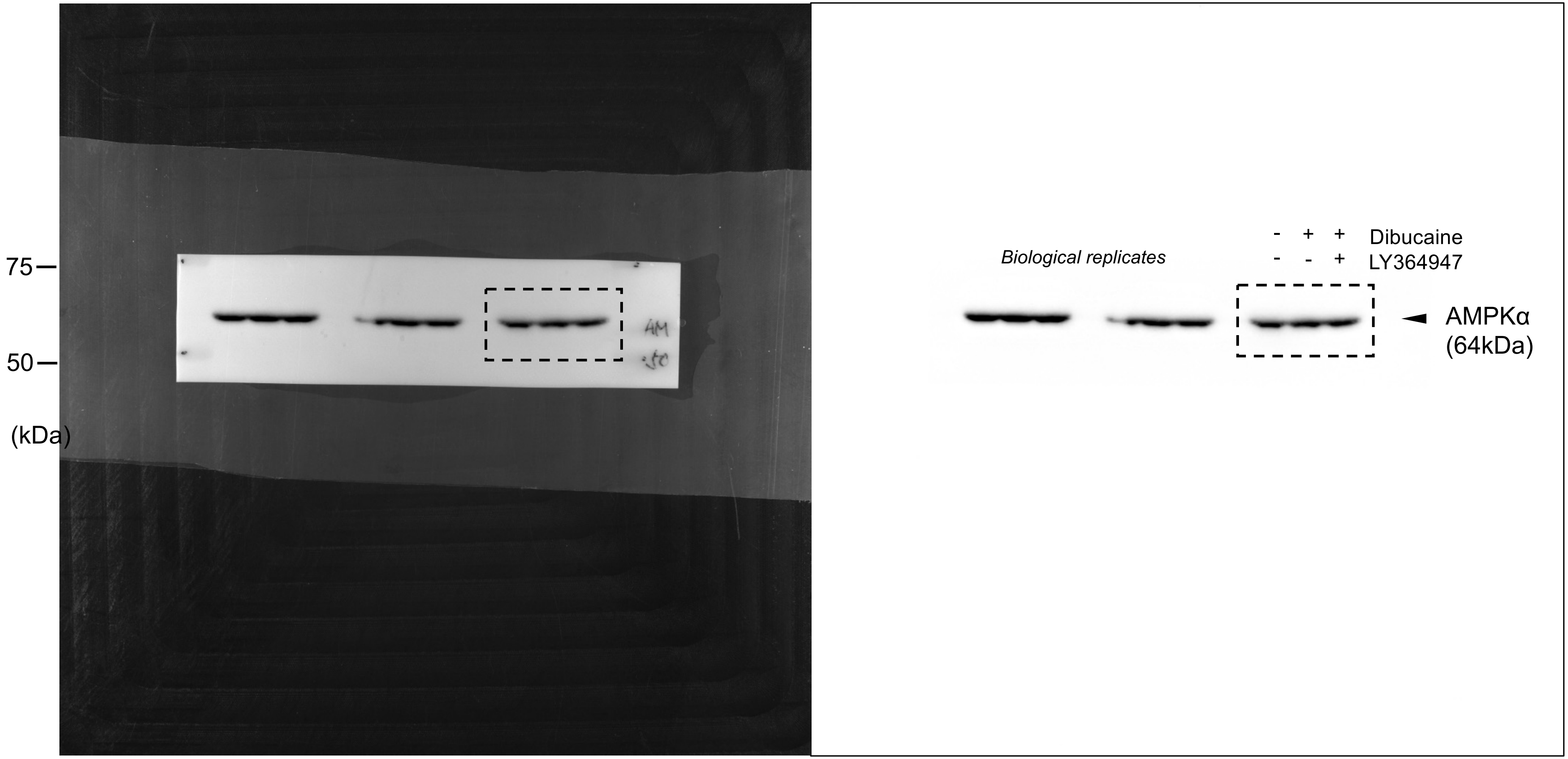

Western Blot Source Data for Figure 5F

αTubulin

Uncropped source data for Figure 5F (αTubulin). Left: Brightfield image of the membrane with molecular markers. Right: Corresponding chemiluminescence image. Lanes outside the dotted box contain biological replicates. Dotted box indicates the region displayed in Figure 5F.

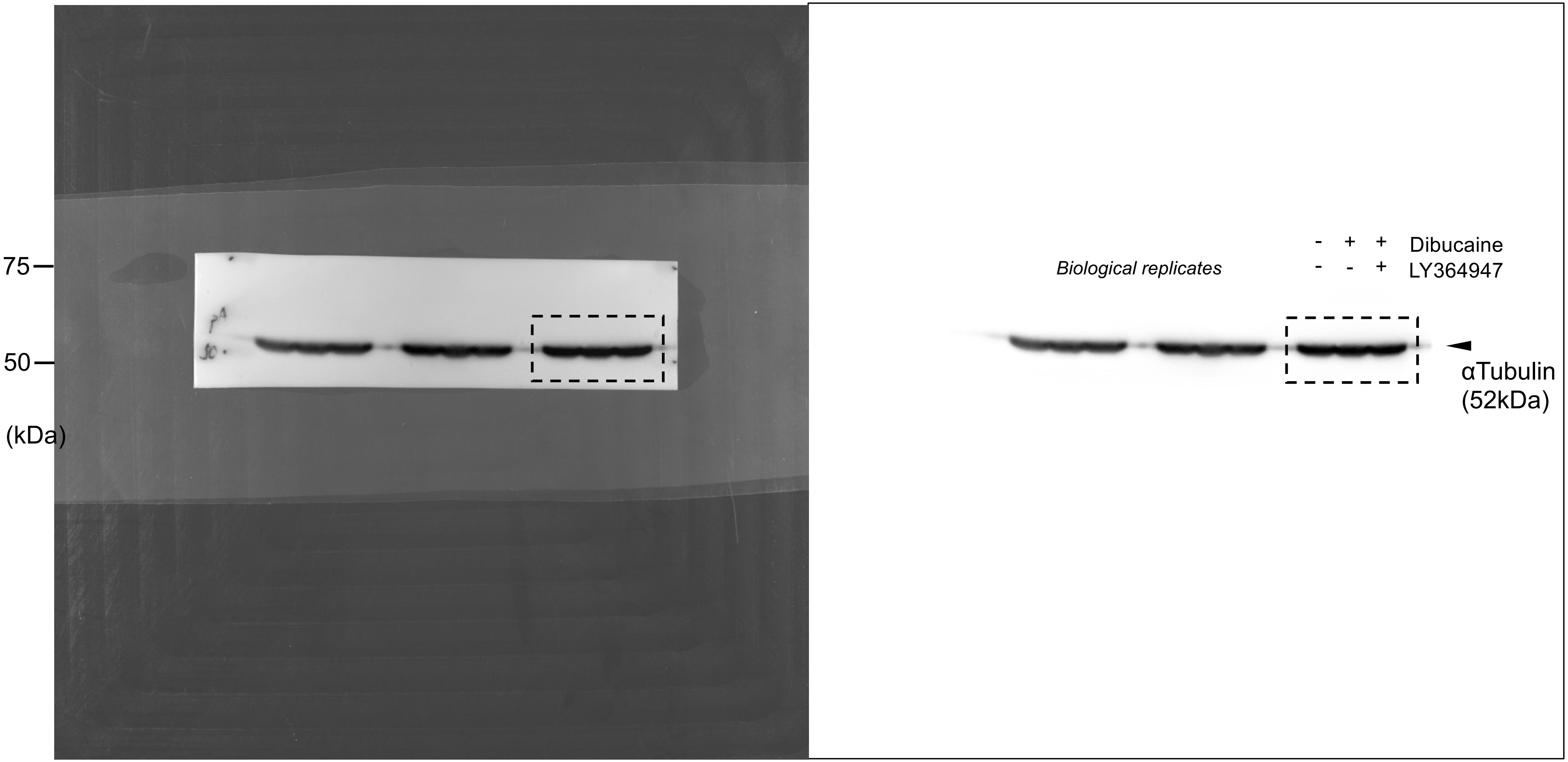

Western Blot Source Data for Figure 6A

LC3

Uncropped source data for Figure 6A (LC3). Left: Brightfield image of the membrane with molecular markers. Right: Corresponding chemiluminescence image. Lanes outside the dotted box contain unrelated samples. Dotted box indicates the region displayed in Figure 6A.

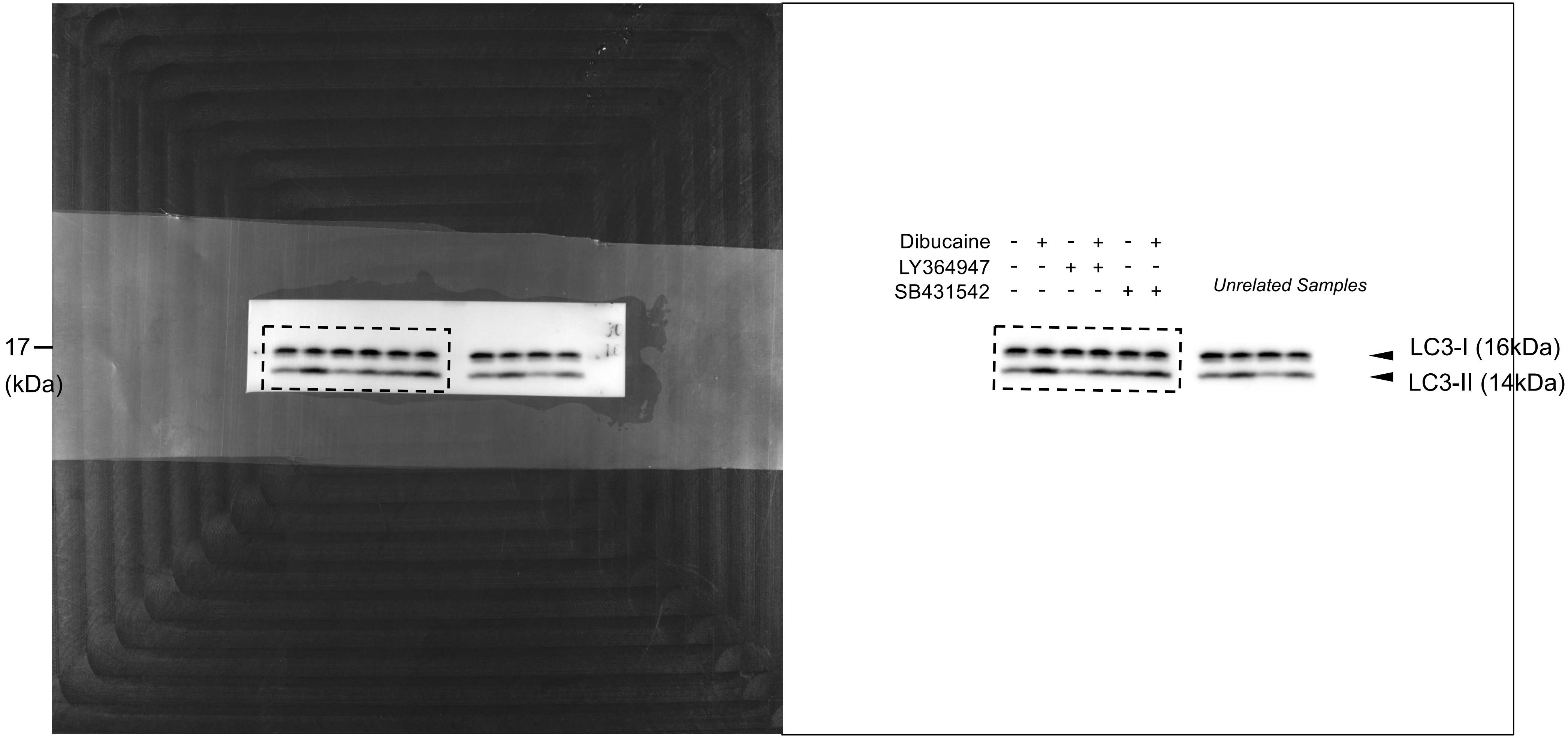

Western Blot Source Data for Figure 6A

Uncropped source data for Figure 6A (TH). Left: Brightfield image of the membrane with molecular markers. Right: Corresponding chemiluminescence image. Lanes outside the dotted box contain unrelated samples. Dotted box indicates the region displayed in Figure 6A.

TH

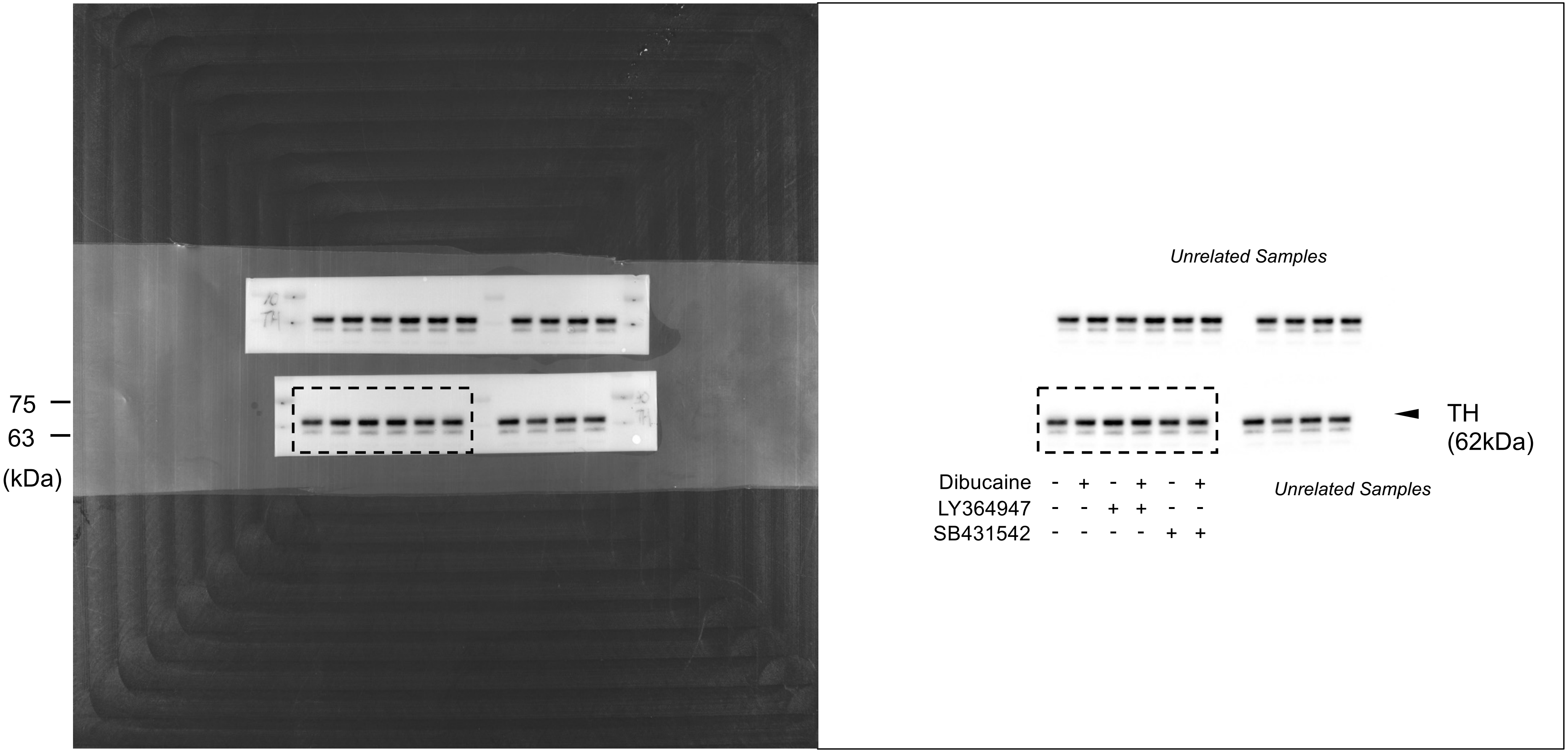

Western Blot Source Data for Figure 6A

GAPDH

Uncropped source data for Figure 6A (GAPDH). Left: Brightfield image of the membrane with molecular markers. Right: Corresponding chemiluminescence image. Lanes outside the dotted box contain unrelated samples. Dotted box indicates the region displayed in Figure 6A.

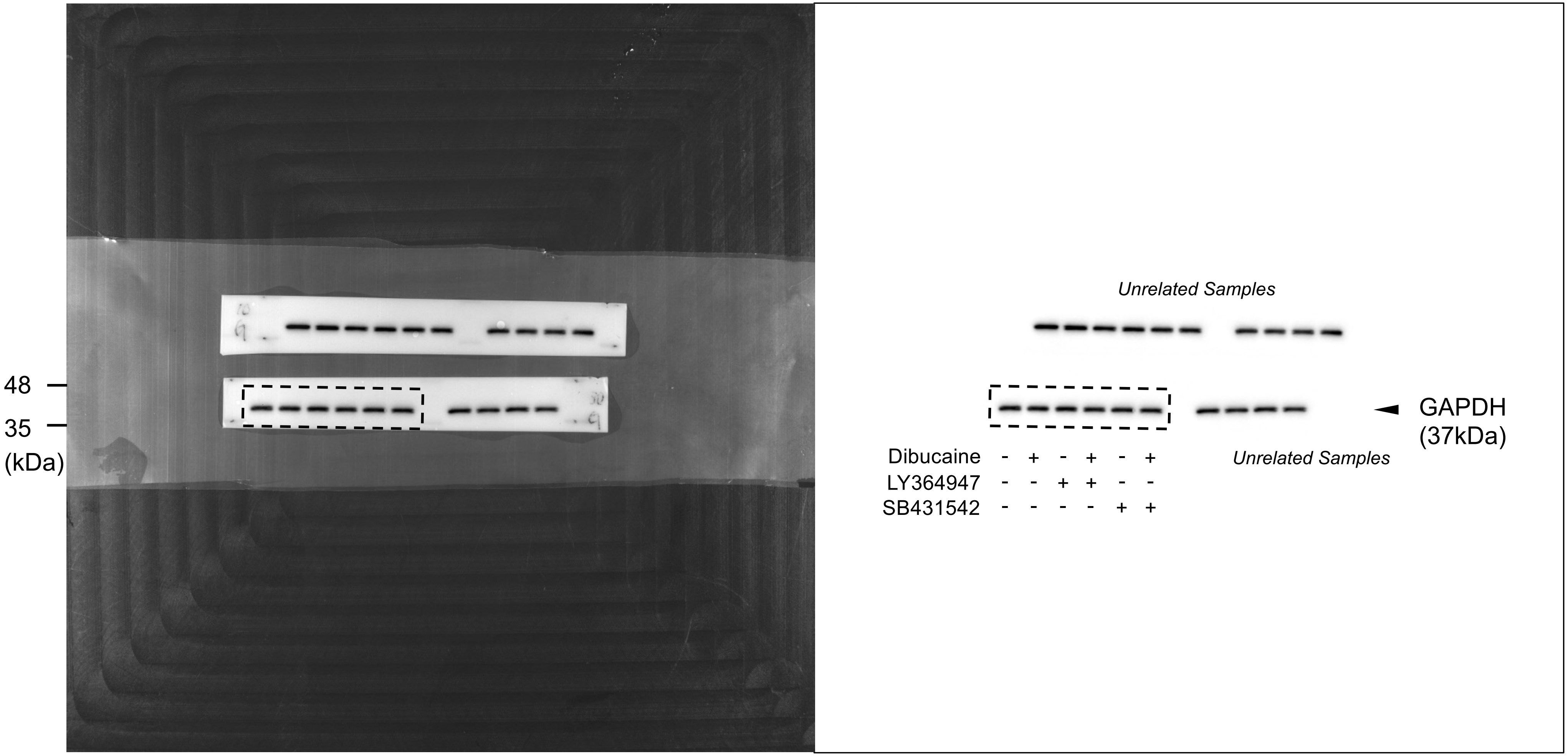

Western Blot Source Data for Figure 6B

p-αSyn

Uncropped source data for Figure 6B (p-αSyn). Left: Brightfield image of the membrane with molecular markers. Right: Corresponding chemiluminescence image. Lanes outside the dotted box contain unrelated samples. Dotted box indicates the region displayed in Figure 6B.

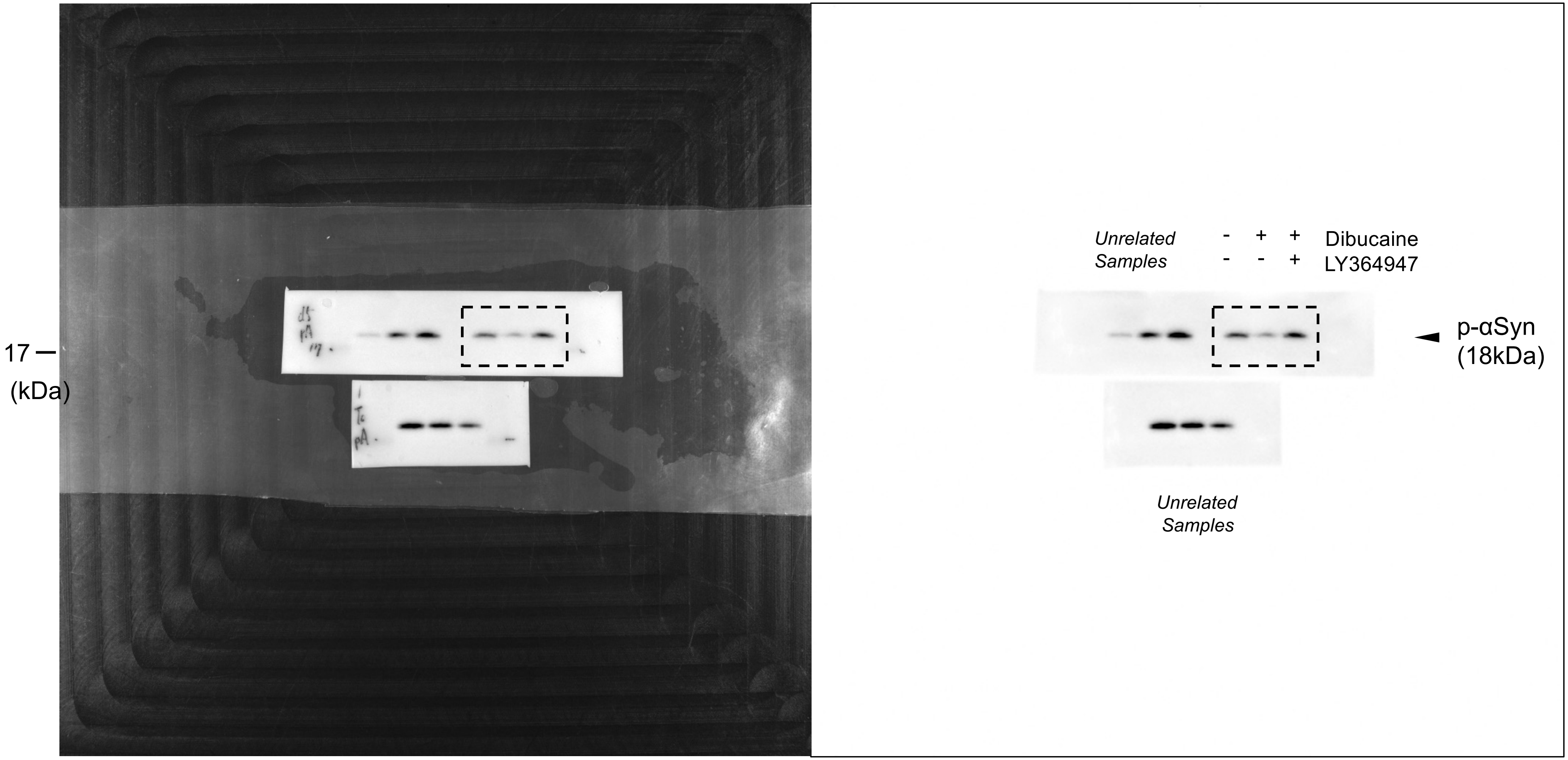

Western Blot Source Data for Figure 6B

Uncropped source data for Figure 6B (αSyn). Left: Brightfield image of the membrane with molecular markers. Right: Corresponding chemiluminescence image. Lanes outside the dotted box contain unrelated samples. Dotted box indicates the region displayed in Figure 6B.

αSyn

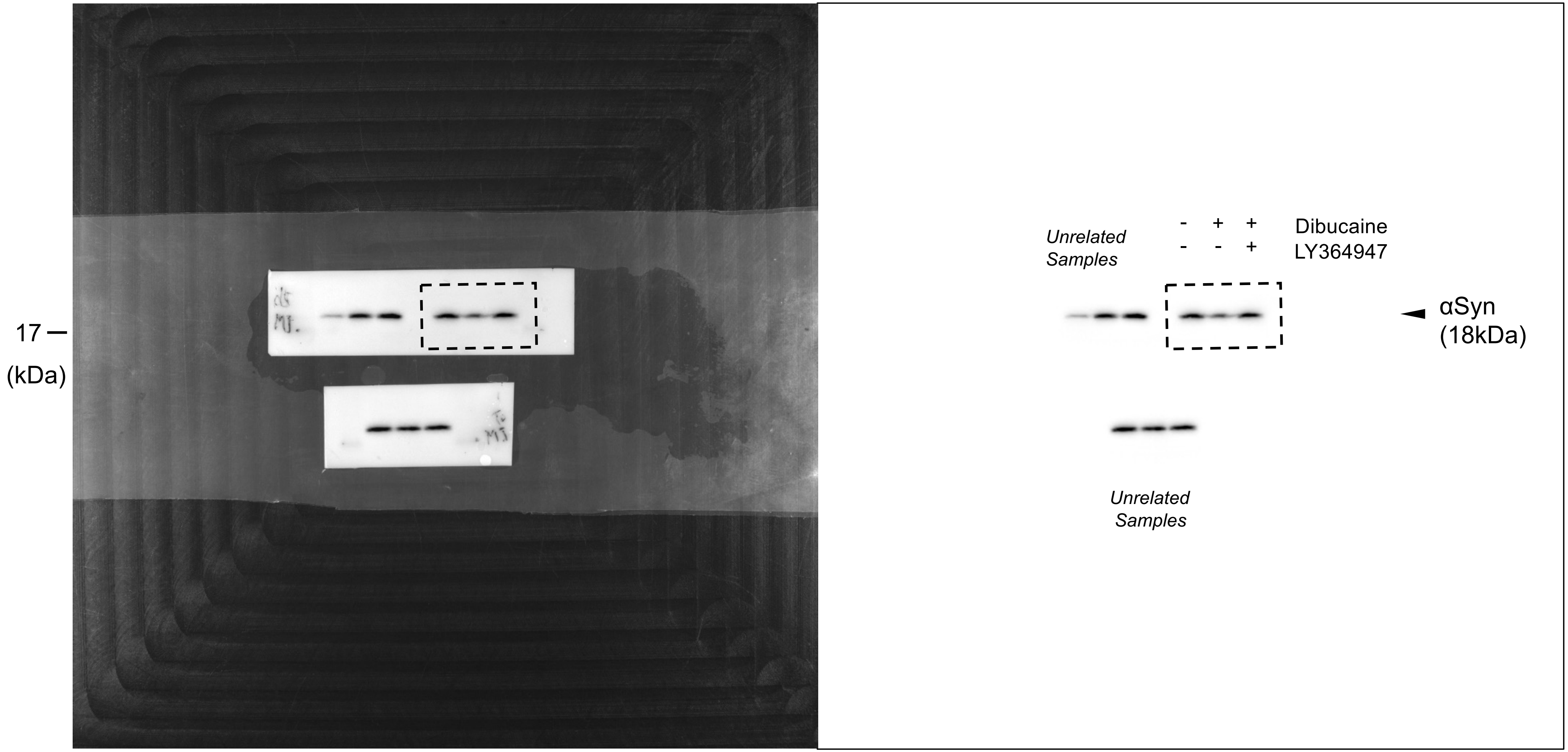

Western Blot Source Data for Figure 6B

Uncropped source data for Figure 6B (TH). Left: Brightfield image of the membrane with molecular markers. Right: Corresponding chemiluminescence image. Lanes outside the dotted box contain unrelated samples. Dotted box indicates the region displayed in Figure 6B.

TH

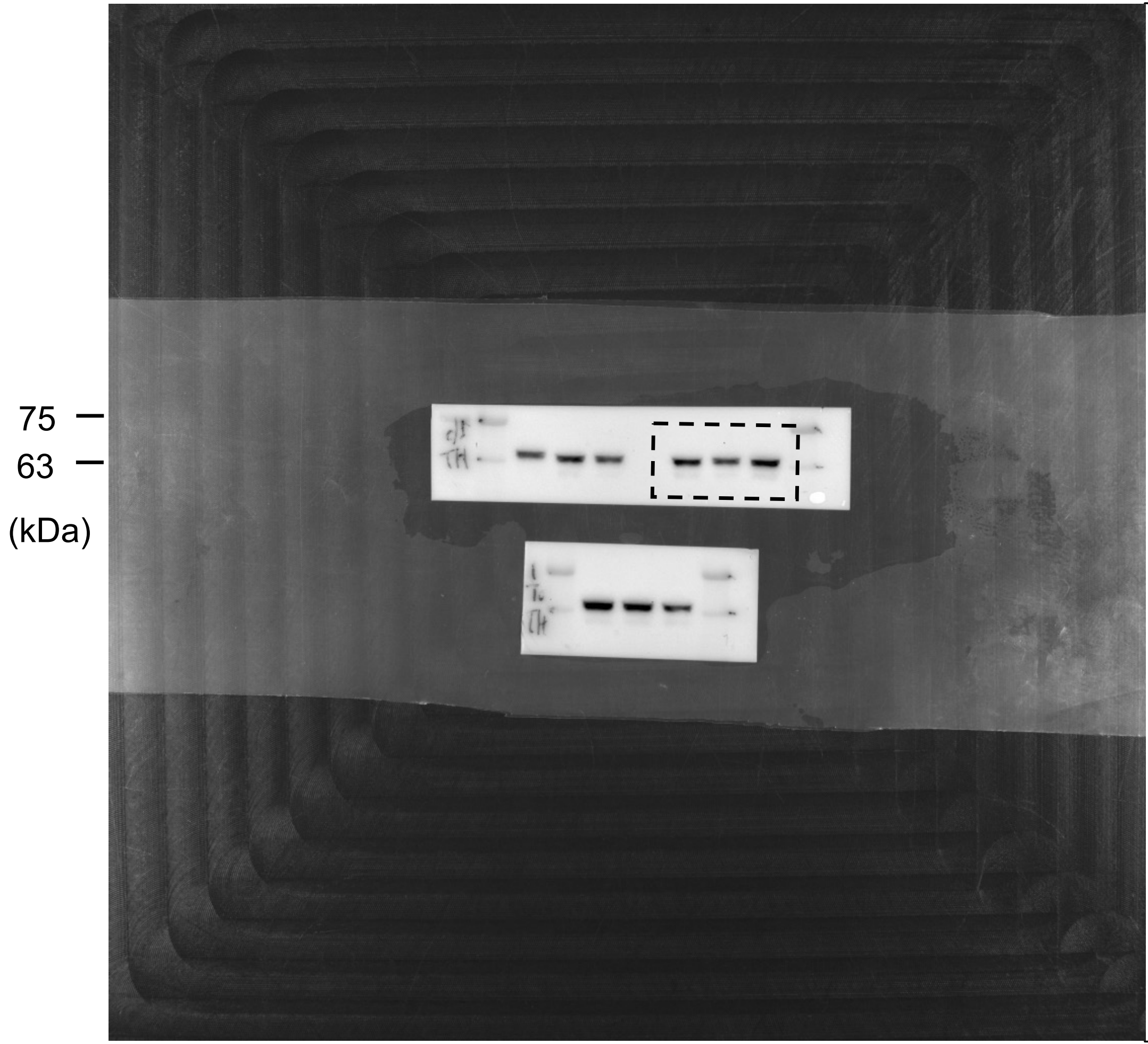

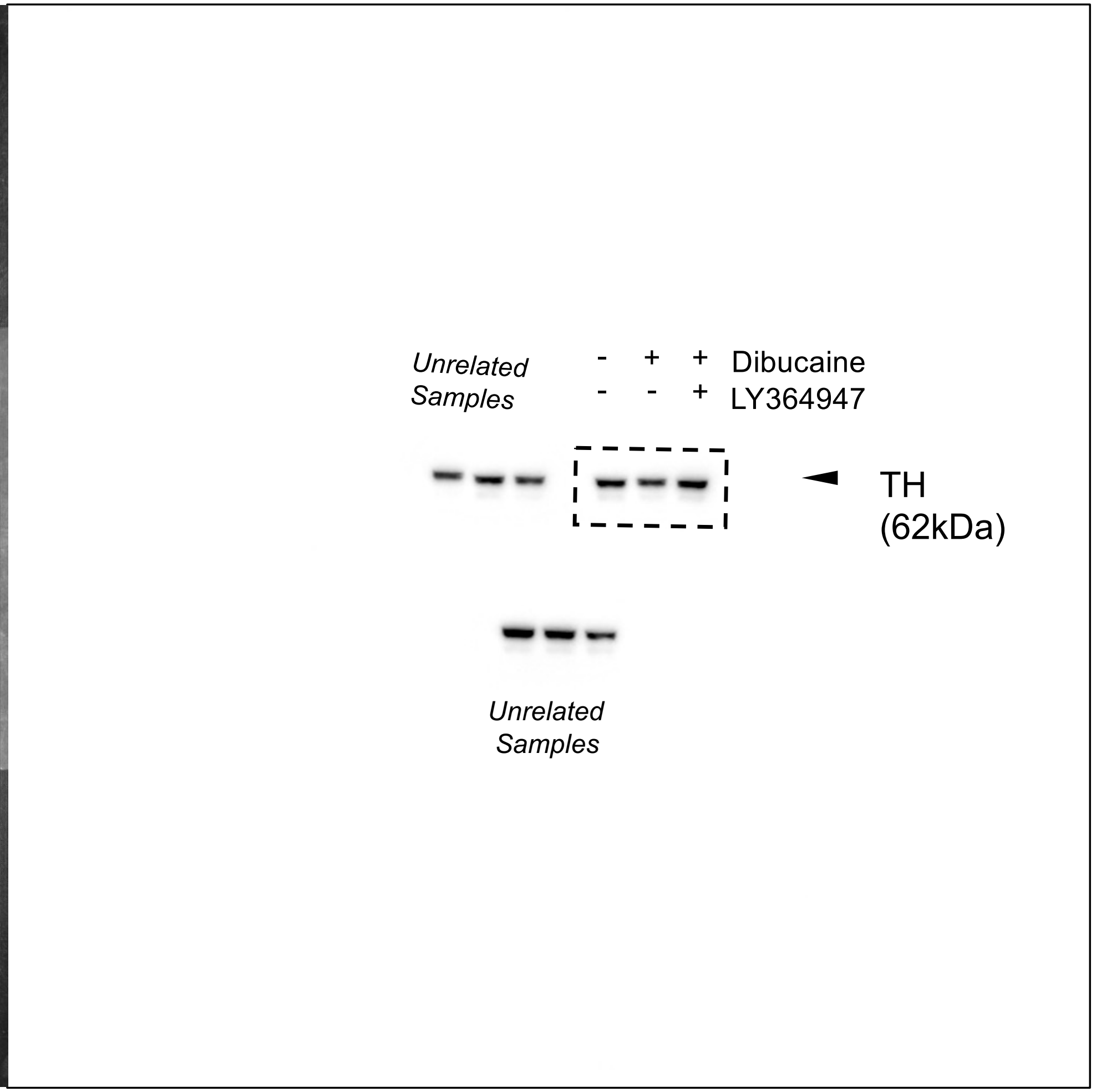

Western Blot Source Data for Figure 6B

GAPDH

Uncropped source data for Figure 6B (GAPDH). Left: Brightfield image of the membrane with molecular markers. Right: Corresponding chemiluminescence image. Lanes outside the dotted box contain unrelated samples. Dotted box indicates the region displayed in Figure 6B.

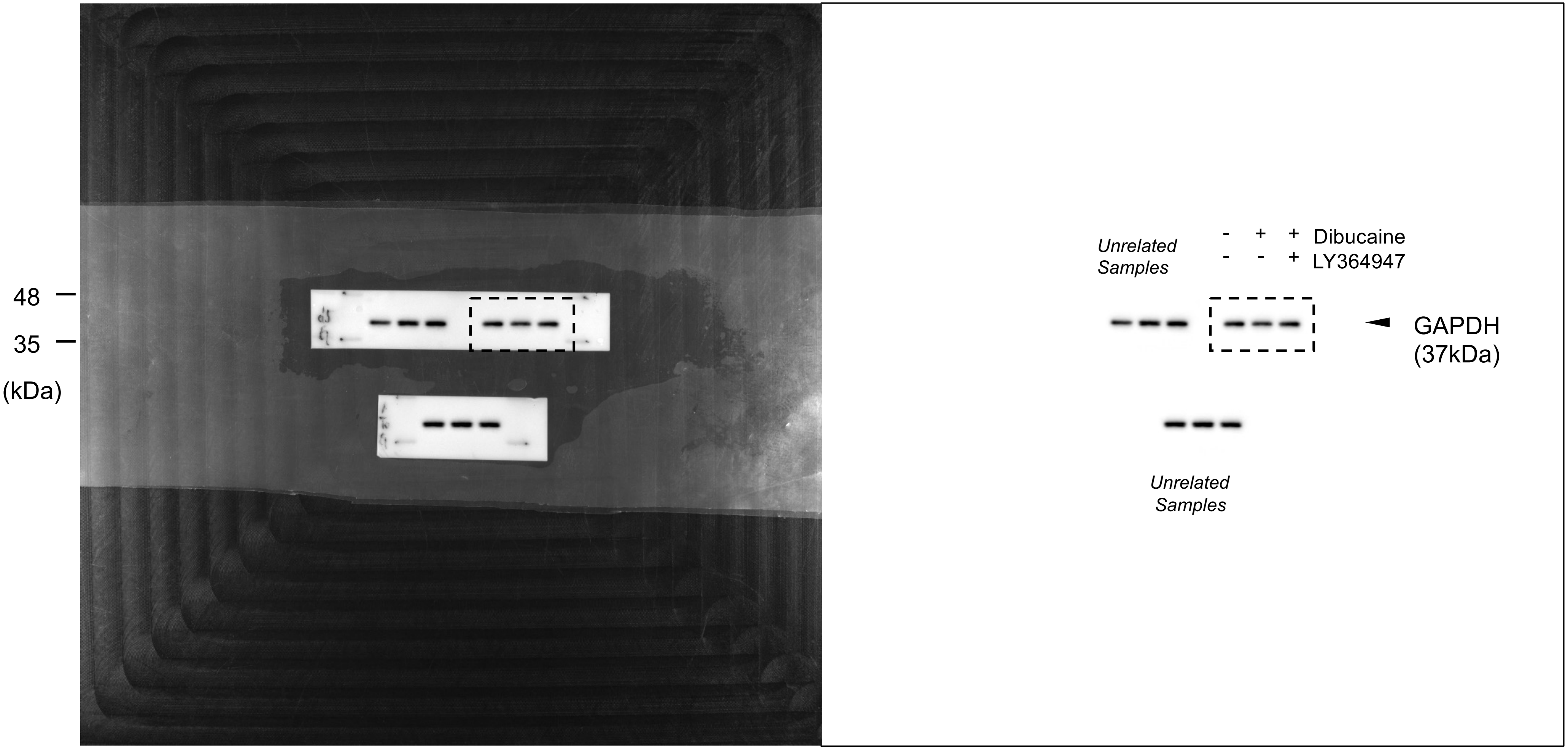

Western Blot Source Data for Supplementary Figure S2A

LC3
DMSO, Torin-1

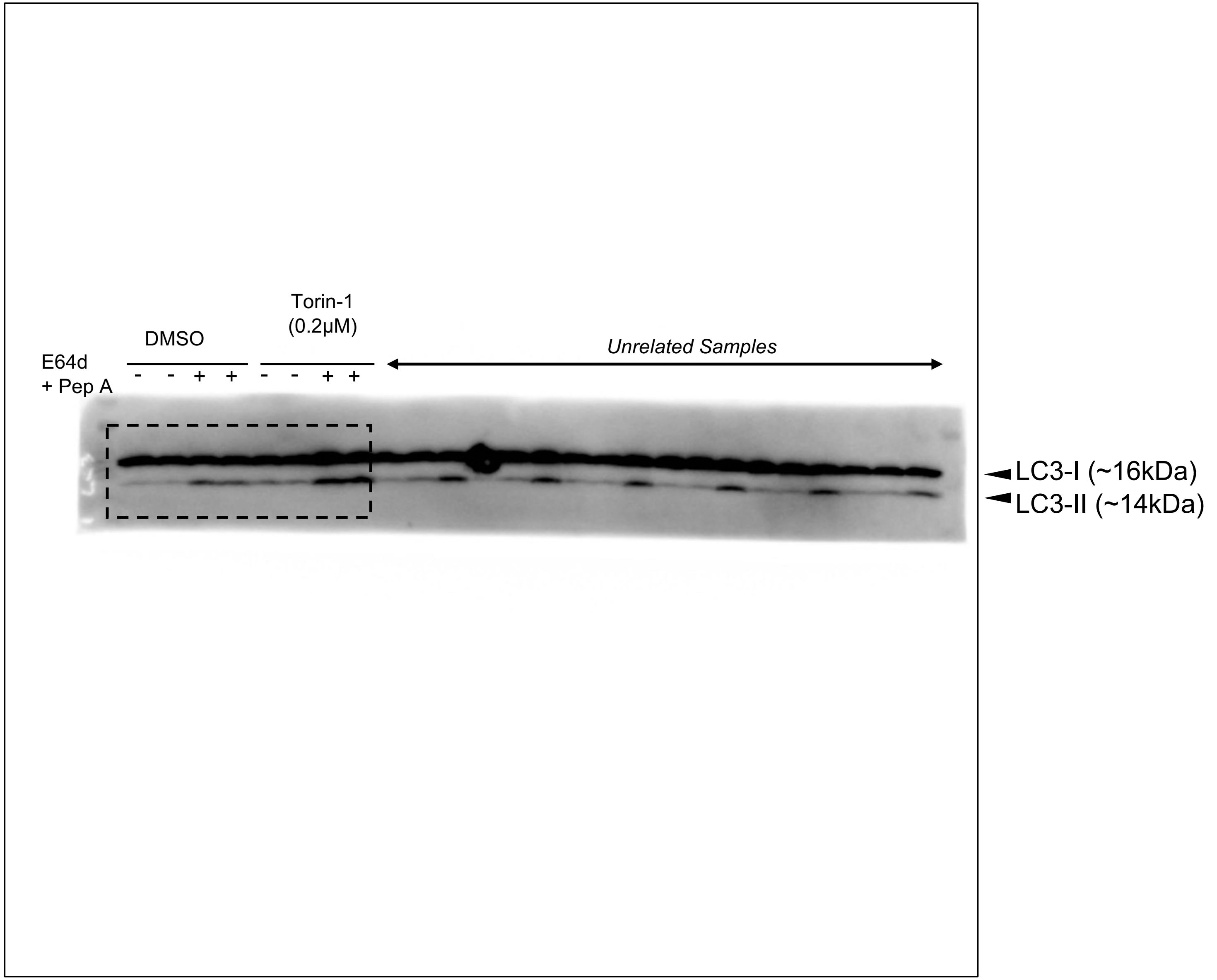

Uncropped original membrane image for Supplementary Figure S2A. A molecular-weight marker lane was not captured or retained in the available image file. The expected molecular weights of LC3-I and LC3-II are approximately 16 kDa and 14 kDa, respectively. The expected molecular weights are provided for reference and do not represent molecular-weight markers measured in this image. Lanes outside the dotted box contain unrelated samples.

Western Blot Source Data for Supplementary Figure S2A

GAPDH
DMSO, Torin-1

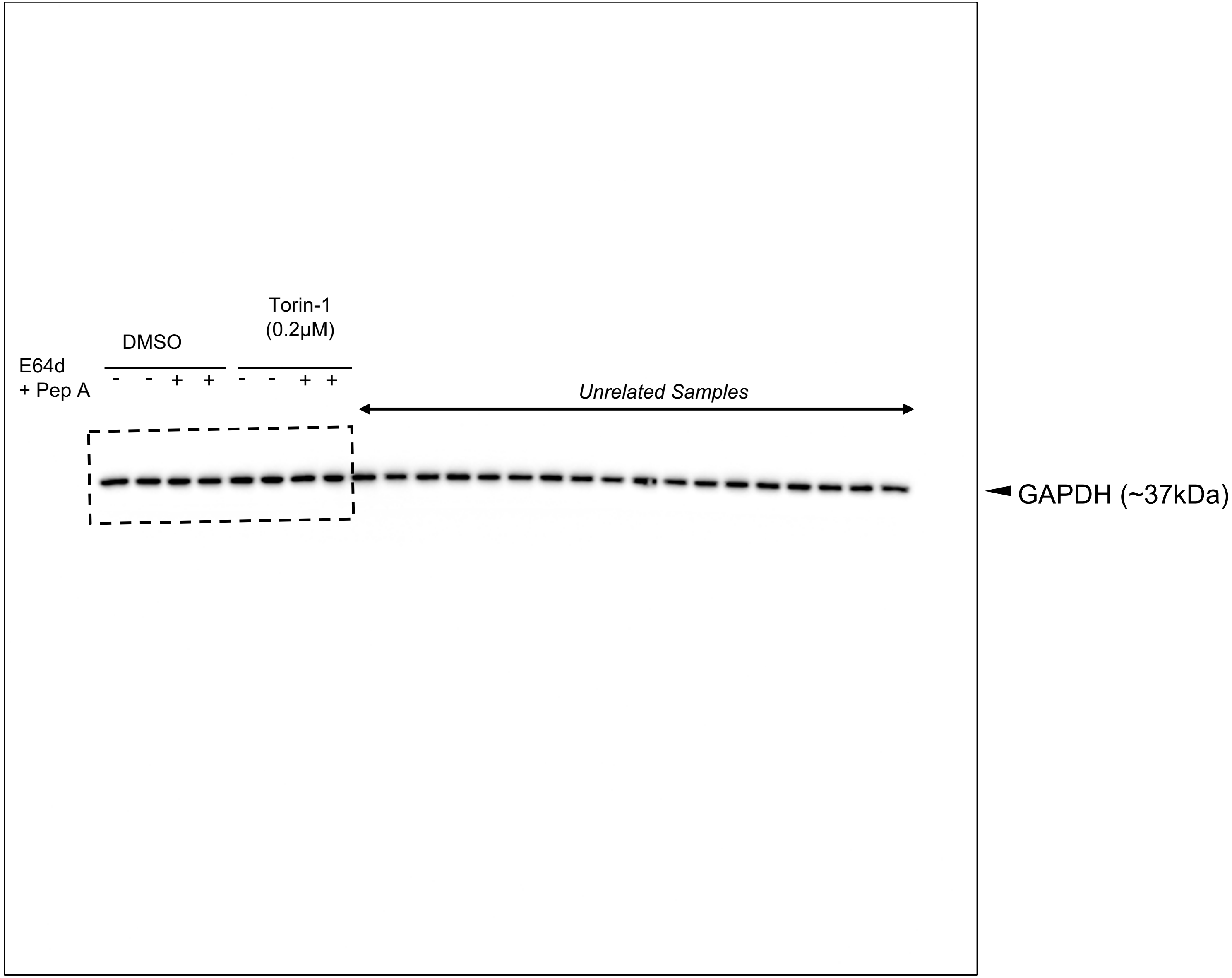

The original uncropped blot is shown. A molecular-weight marker lane was not captured or retained in the available image file. The expected molecular weight of GAPDH is approximately 37 kDa. The expected molecular weight is provided for reference and does not represent a molecular-weight marker measured in this image. Lanes outside the dotted box contain unrelated samples.

Western Blot Source Data for Supplementary Figure S2A

LC3,
DMSO / Bromocriptine

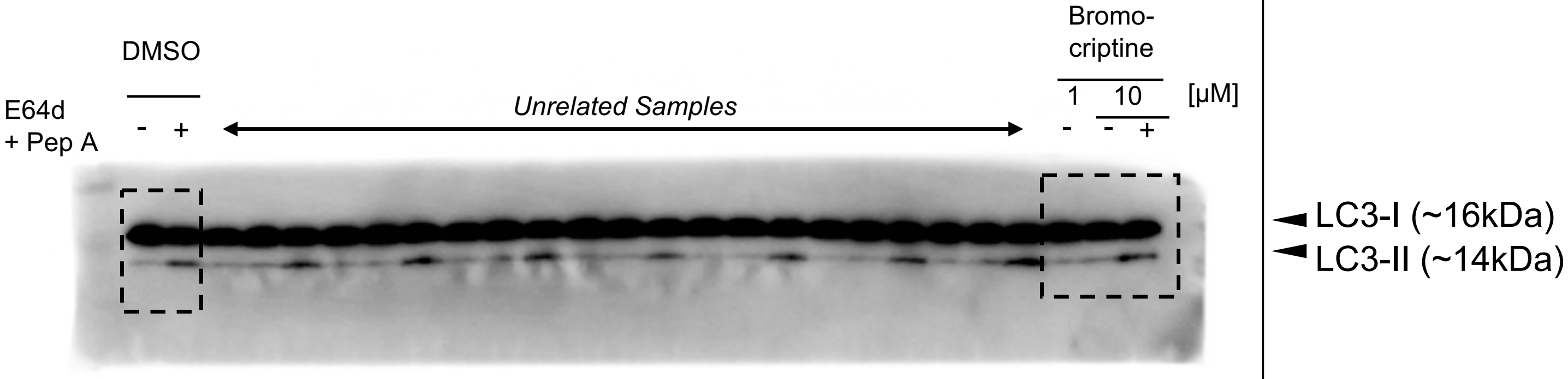

Uncropped original membrane image for Supplementary Figure S2A. A molecular-weight marker lane was not captured or retained in the available image file. The expected molecular weights of LC3-I and LC3-II are approximately 16 kDa and 14 kDa, respectively. The expected molecular weights are provided for reference and do not represent molecular-weight markers measured in this image. Lanes outside the dotted box contain unrelated samples.

Western Blot Source Data for Supplementary Figure S2A

GAPDH,
DMSO / Bromocriptine

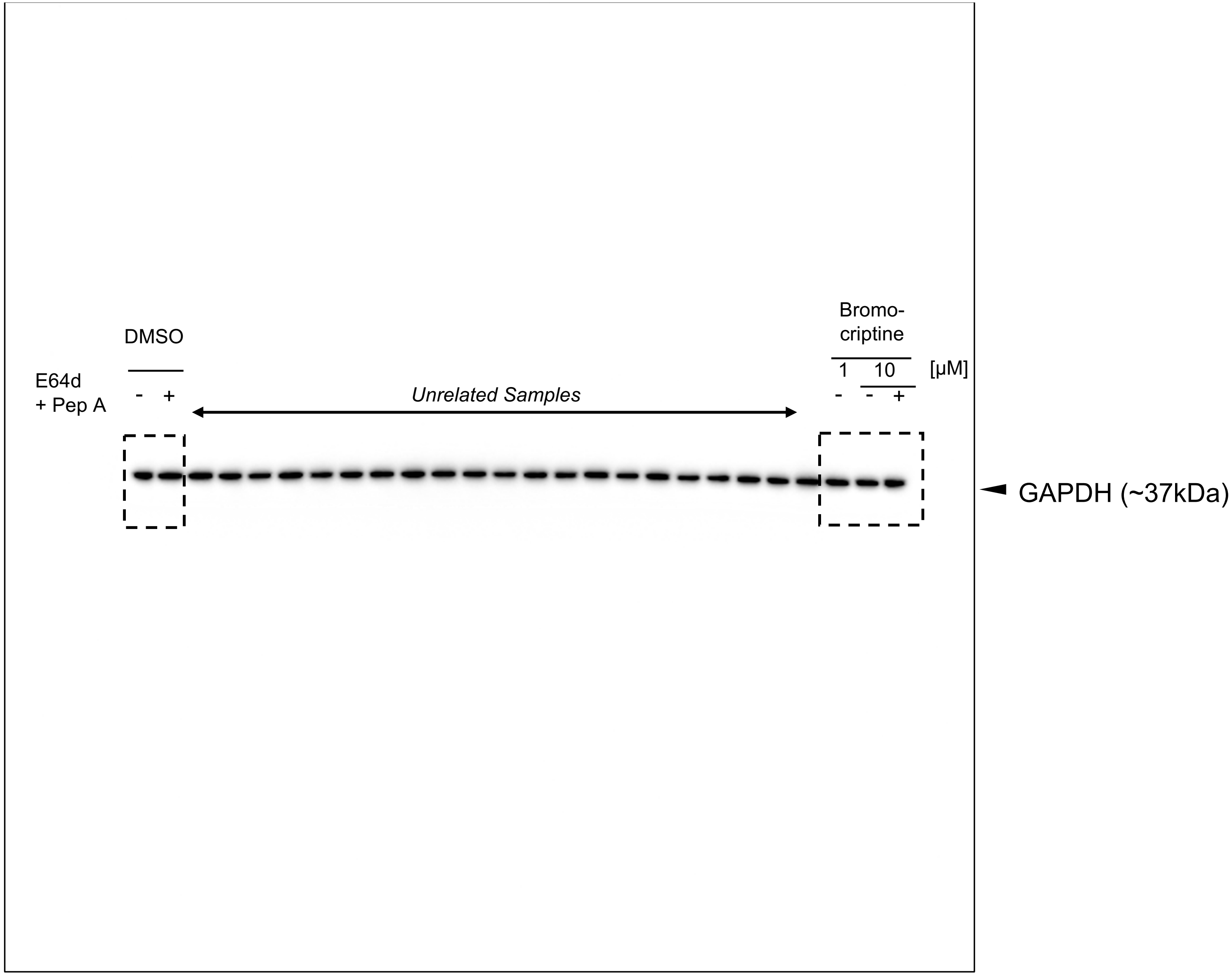

The original uncropped blot is shown. A molecular-weight marker lane was not captured or retained in the available image file. The expected molecular weight of GAPDH is approximately 37 kDa. The expected molecular weight is provided for reference and does not represent a molecular-weight marker measured in this image. Lanes outside the dotted box contain unrelated samples.

Western Blot Source Data for Supplementary Figure S2A

LC3,
DMSO, Dibucaine / Memantine-MaxiPost

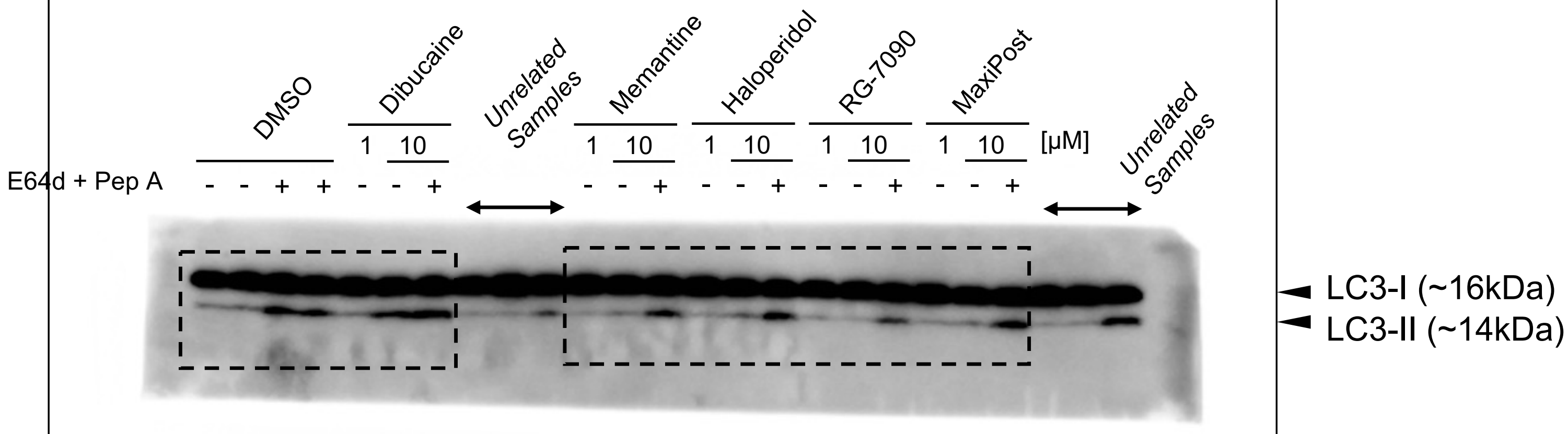

Uncropped original membrane image for Supplementary Figure S2A. A molecular-weight marker lane was not captured or retained in the available image file. The expected molecular weights of LC3-I and LC3-II are approximately 16 kDa and 14 kDa, respectively. The expected molecular weights are provided for reference and do not represent molecular-weight markers measured in this image. Lanes outside the dotted box contain unrelated samples.

Western Blot Source Data for Supplementary Figure S2A

GAPDH,
DMSO, Dibucaine / Memantine-MaxiPost

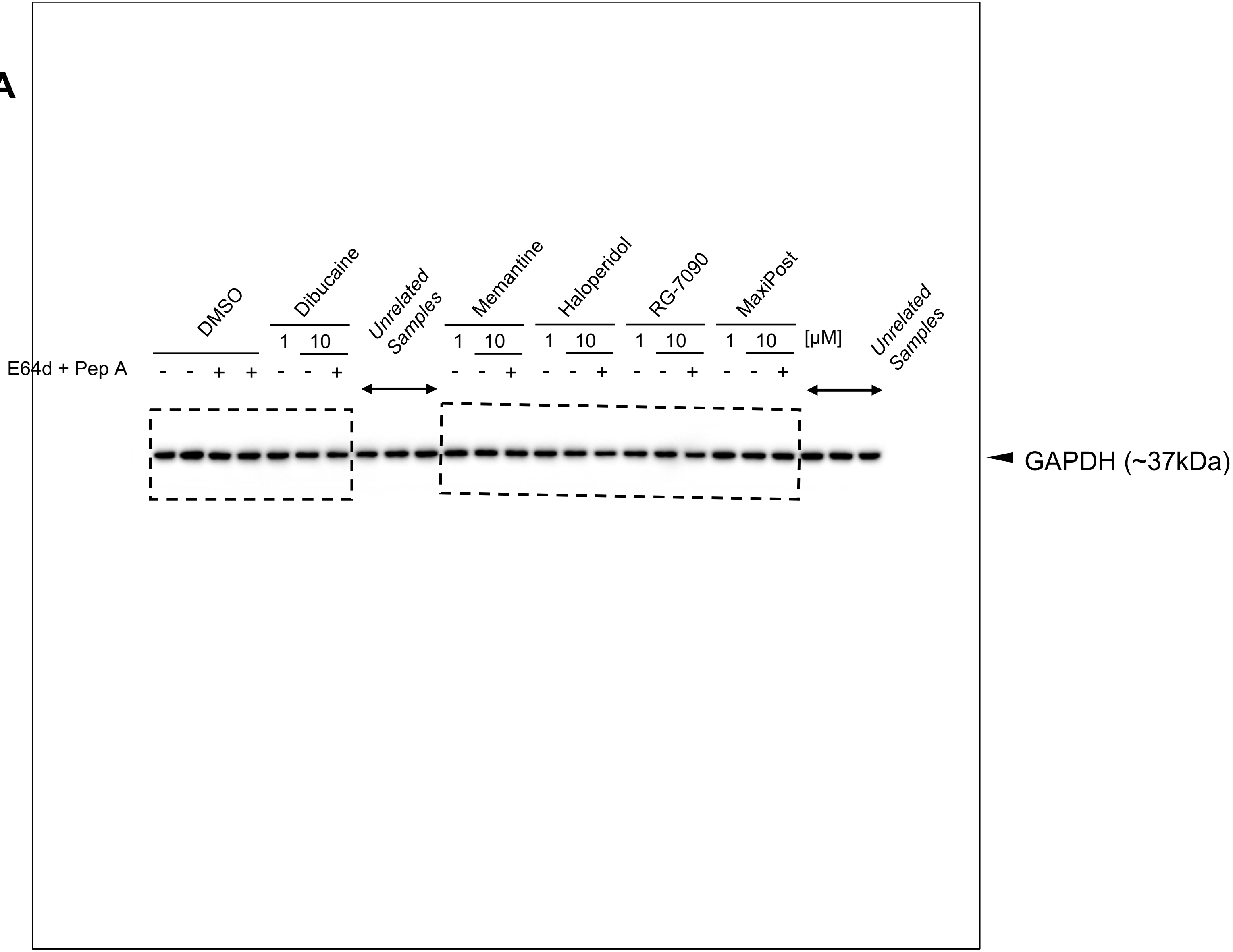

The original uncropped blot is shown. A molecular-weight marker lane was not captured or retained in the available image file. The expected molecular weight of GAPDH is approximately 37 kDa. The expected molecular weight is provided for reference and does not represent a molecular-weight marker measured in this image. Lanes outside the dotted box contain unrelated samples.

Western Blot Source Data
for Supplementary Figure S5A (Left)

LC3,
none / Dibucaine – Lidocaine

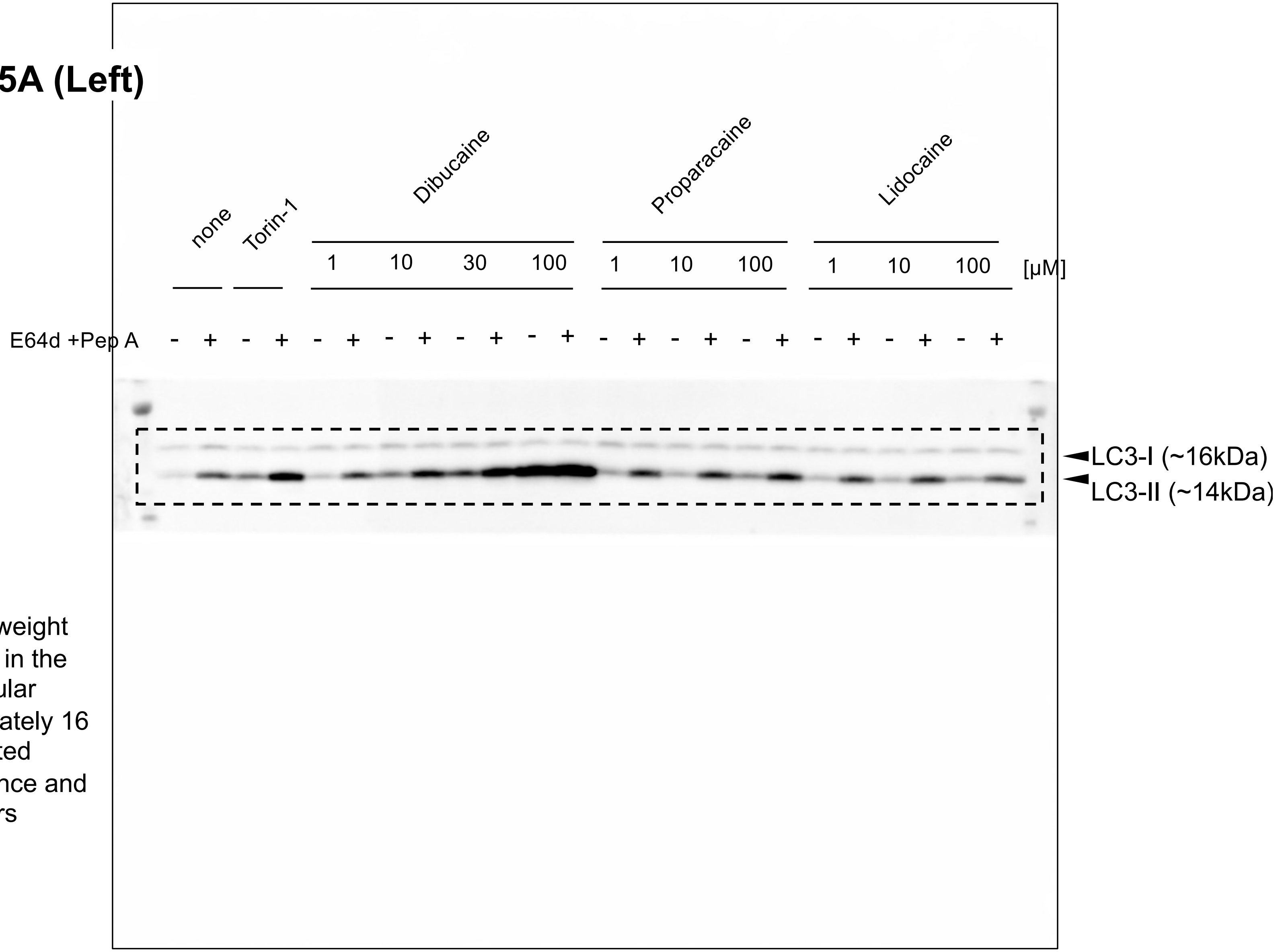

Uncropped original membrane image for Supplementary Figure S5A. A molecular-weight marker lane was not captured or retained in the available image file. The expected molecular weights of LC3-I and LC3-II are approximately 16 kDa and 14 kDa, respectively. The expected molecular weights are provided for reference and do not represent molecular-weight markers measured in this image.

Western Blot Source Data

for Supplementary Figure S5A (Left)

GAPDH,
none, Torin-1 / Dibucaine – Lidocaine

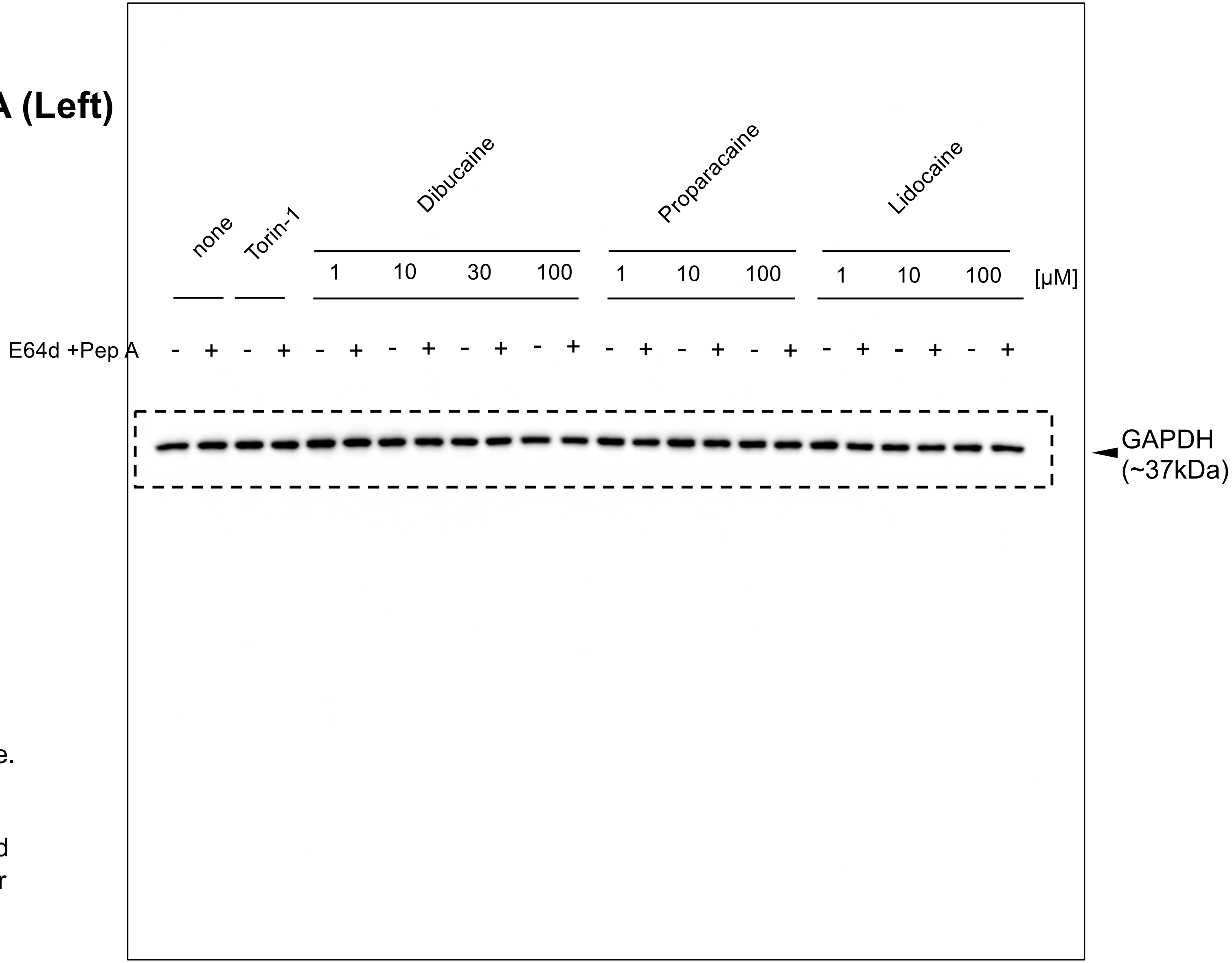

The original uncropped blot is shown. A molecular-weight marker lane was not captured or retained in the available image file. The expected molecular weight of GAPDH is approximately 37 kDa. The expected molecular weight is provided for reference and does not represent a molecular-weight marker measured in this image.

Western Blot Source Data

for Supplementary Figure S5A (Right)

LC3,
none / Torin-1 / Tetracaine

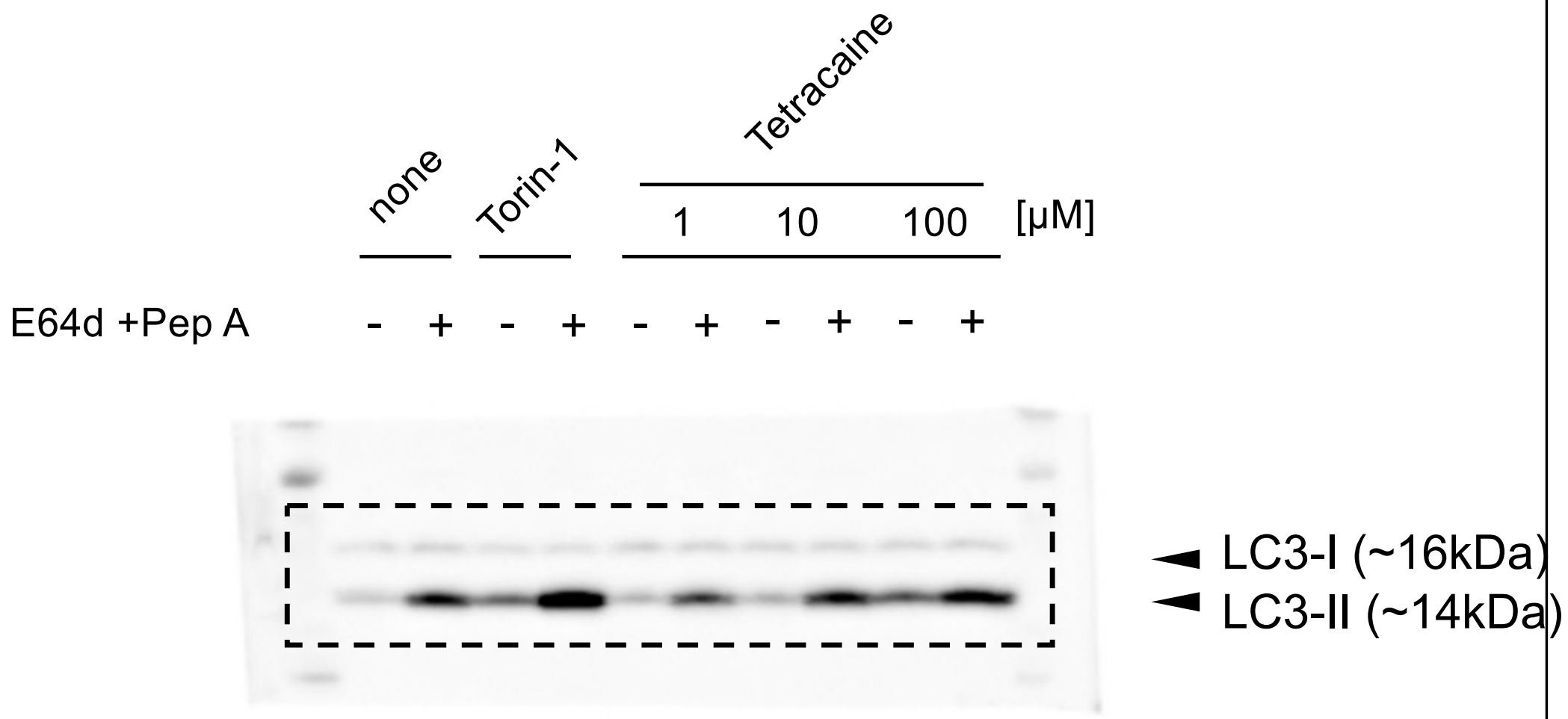

Uncropped original membrane image for Supplementary Figure S5A. A molecular-weight marker lane was not captured or retained in the available image file. The expected molecular weights of LC3-I and LC3-II are approximately 16 kDa and 14 kDa, respectively. The expected molecular weights are provided for reference and do not represent molecular-weight markers measured in this image.

Western Blot Source Data

for Supplementary Figure S5A (Right)

GAPDH
none / Torin-1 / Tetracaine

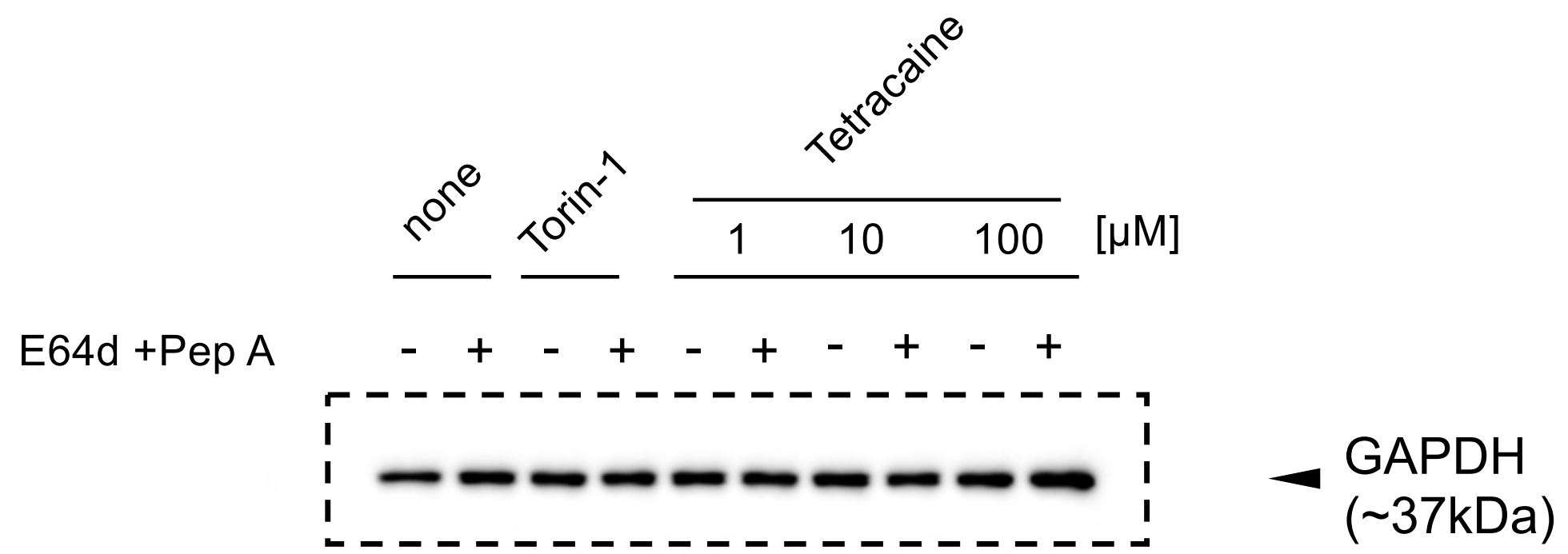

The original uncropped blot is shown. A molecular-weight marker lane was not captured or retained in the available image file. The expected molecular weight of GAPDH is approximately 37 kDa. The expected molecular weight is provided for reference and does not represent a molecular-weight marker measured in this image.

Western Blot Source Data

for Supplementary Figure S5B

p-mTOR (S2448)

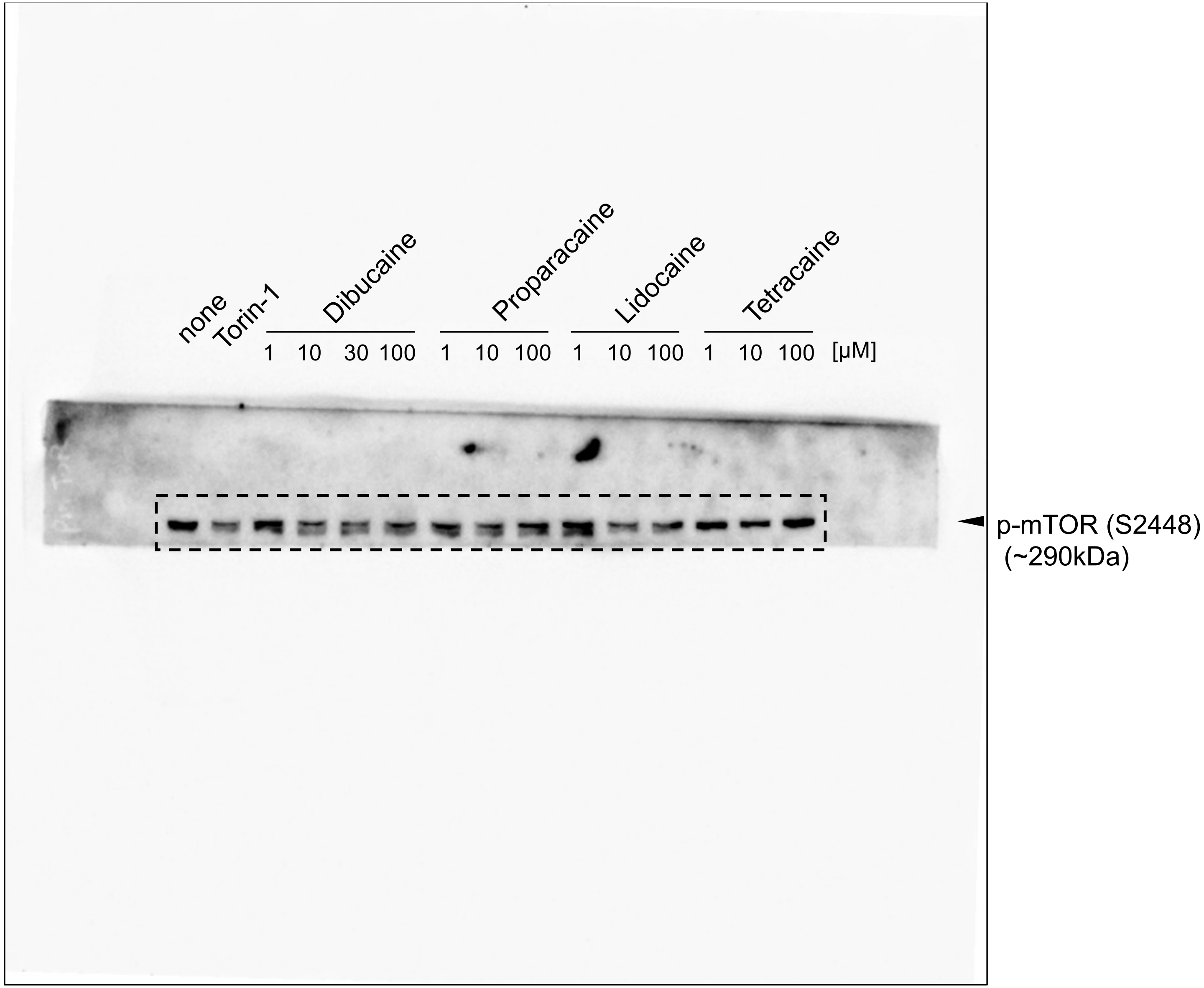

Uncropped original membrane image for Supplementary Figure S5B. A molecular-weight marker lane was not captured or retained in the available image file. The expected molecular weight of p-mTOR(S2448) is approximately 290 kDa. The expected molecular weight is provided for reference and does not represent a molecular-weight marker measured in this image.

Western Blot Source Data

for Supplementary Figure S5B

mTOR

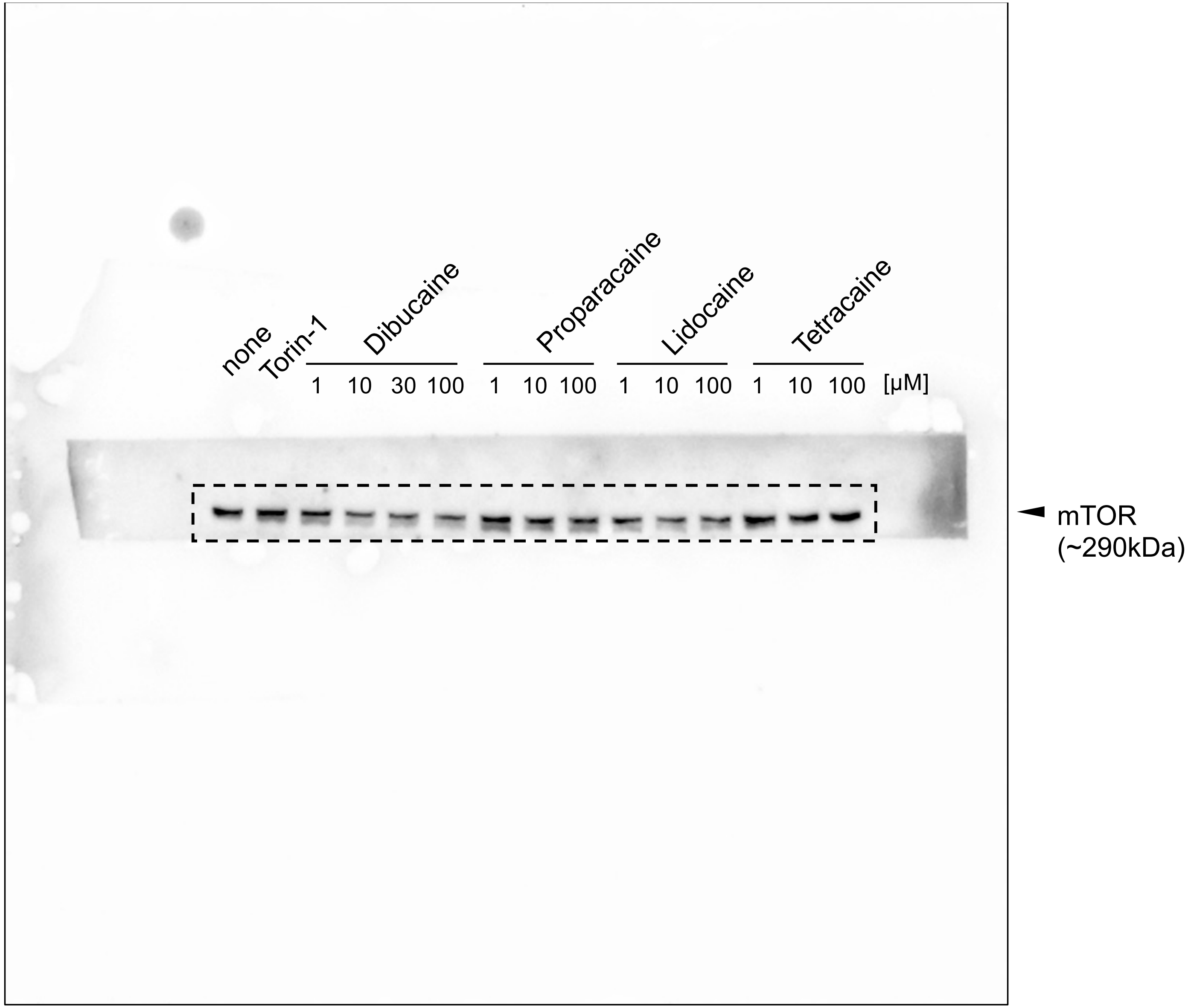

Uncropped original membrane image for Supplementary Figure S5B. A molecular-weight marker lane was not captured or retained in the available image file. The expected molecular weight of mTOR is approximately 290 kDa. The expected molecular weight is provided for reference and does not represent a molecular-weight marker measured in this image.

Western Blot Source Data

for Supplementary Figure S5B

p-p70S6 (T389)

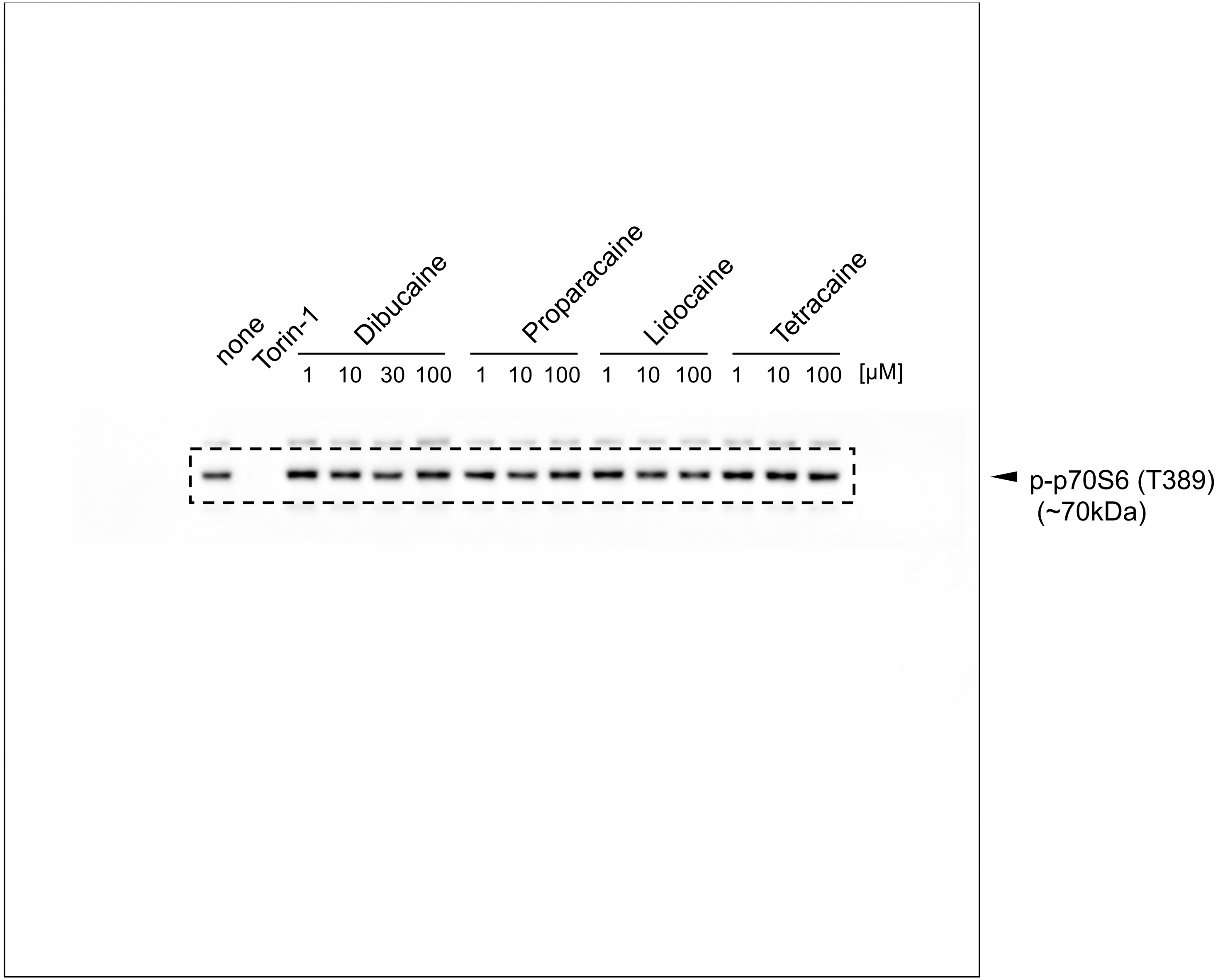

Uncropped original membrane image for Supplementary Figure S5B. A molecular-weight marker lane was not captured or retained in the available image file. The expected molecular weight of p-p70S6(T389) is approximately 70 kDa. The expected molecular weight is provided for reference and does not represent a molecular-weight marker measured in this image.

Western Blot Source Data

for Supplementary Figure S5B

p70S6

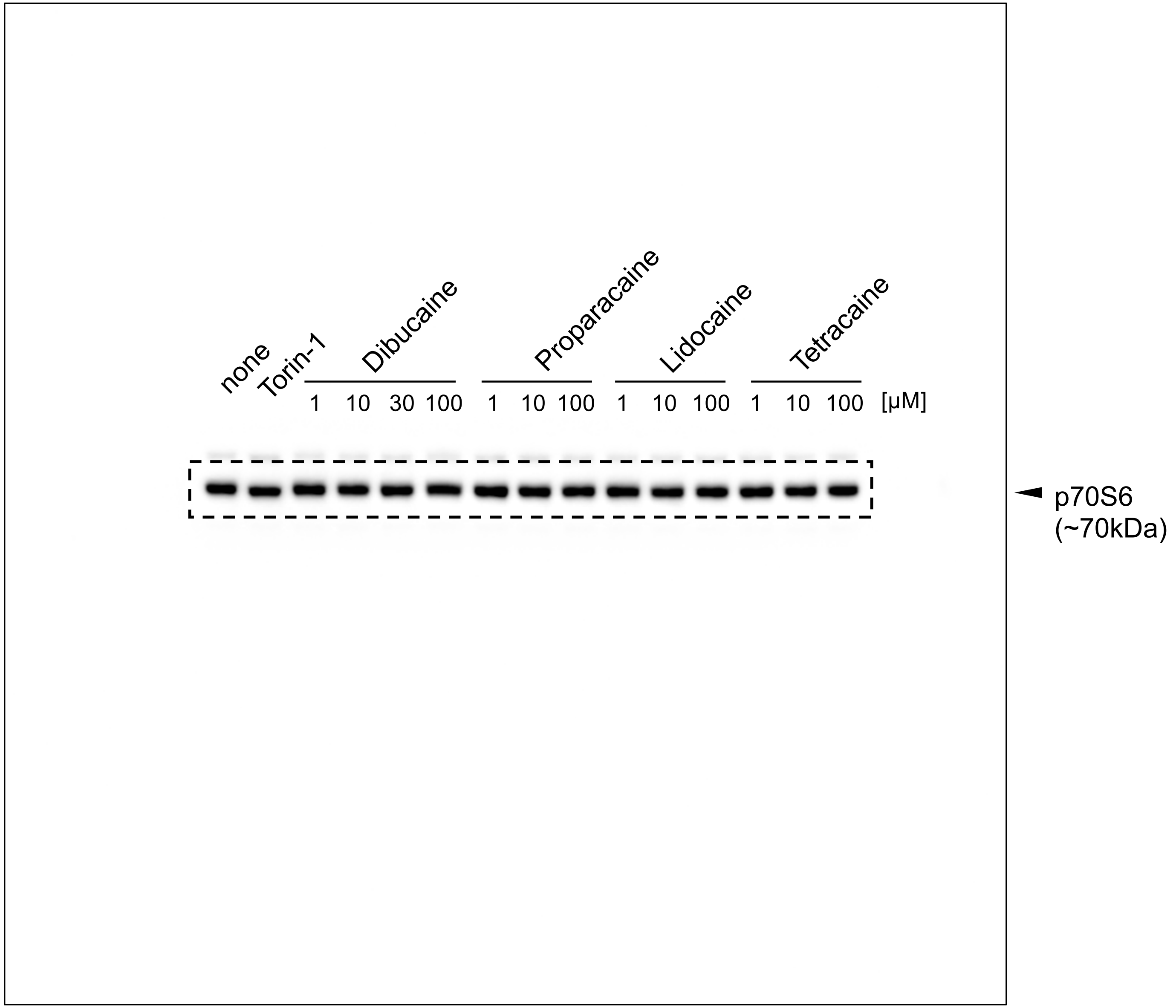

Uncropped original membrane image for Supplementary Figure S5B. A molecular-weight marker lane was not captured or retained in the available image file. The expected molecular weight of p70S6 is approximately 70 kDa. The expected molecular weight is provided for reference and does not represent a molecular-weight marker measured in this image.

Western Blot Source Data

for Supplementary Figure S5B

p-S6 (S235/236)

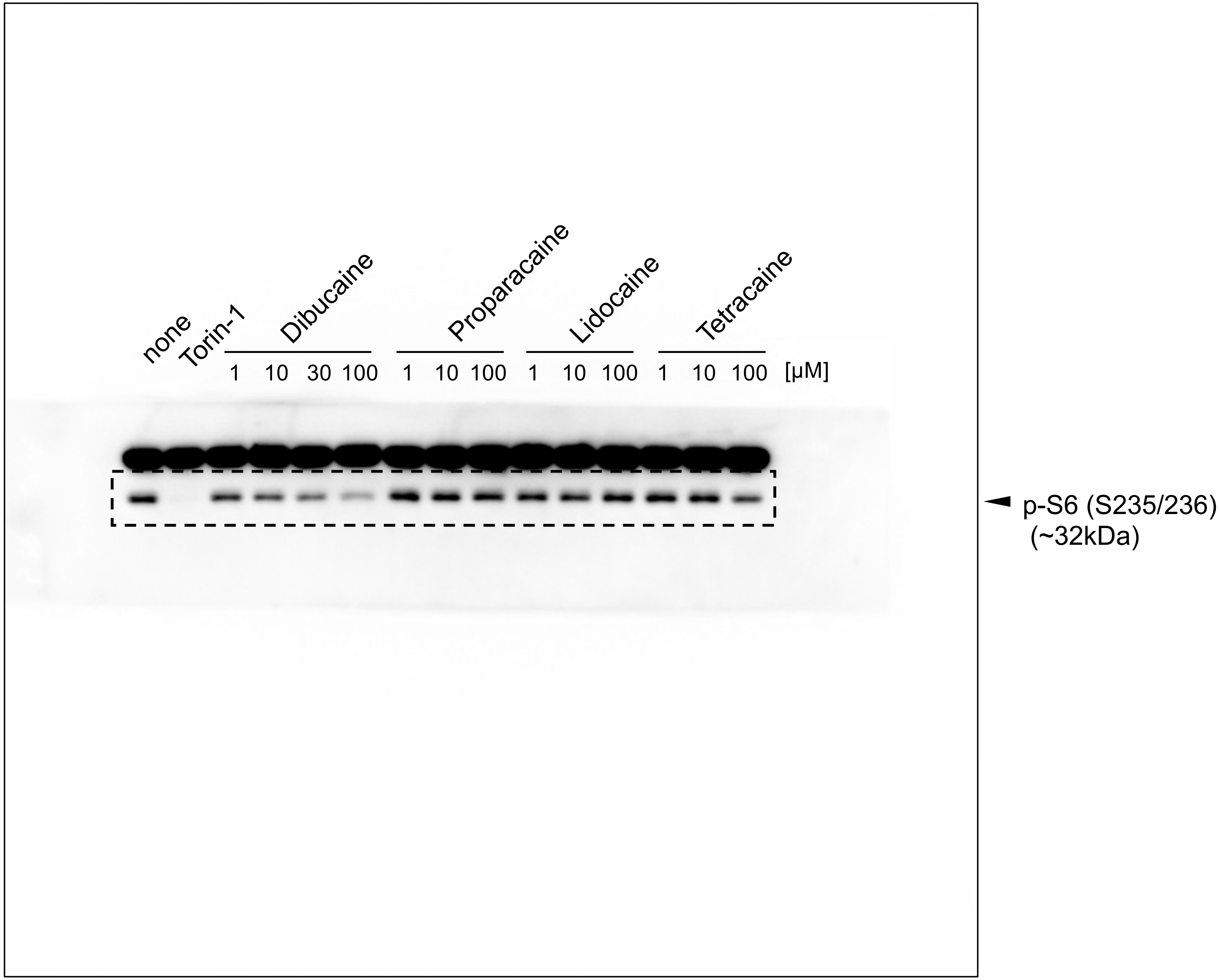

Uncropped original membrane image for Supplementary Figure S5B. A molecular-weight marker lane was not captured or retained in the available image file. The expected molecular weight of p-S6(S235/236) is approximately 32 kDa. The expected molecular weight is provided for reference and does not represent a molecular-weight marker measured in this image.

Western Blot Source Data

for Supplementary Figure S5B

S6

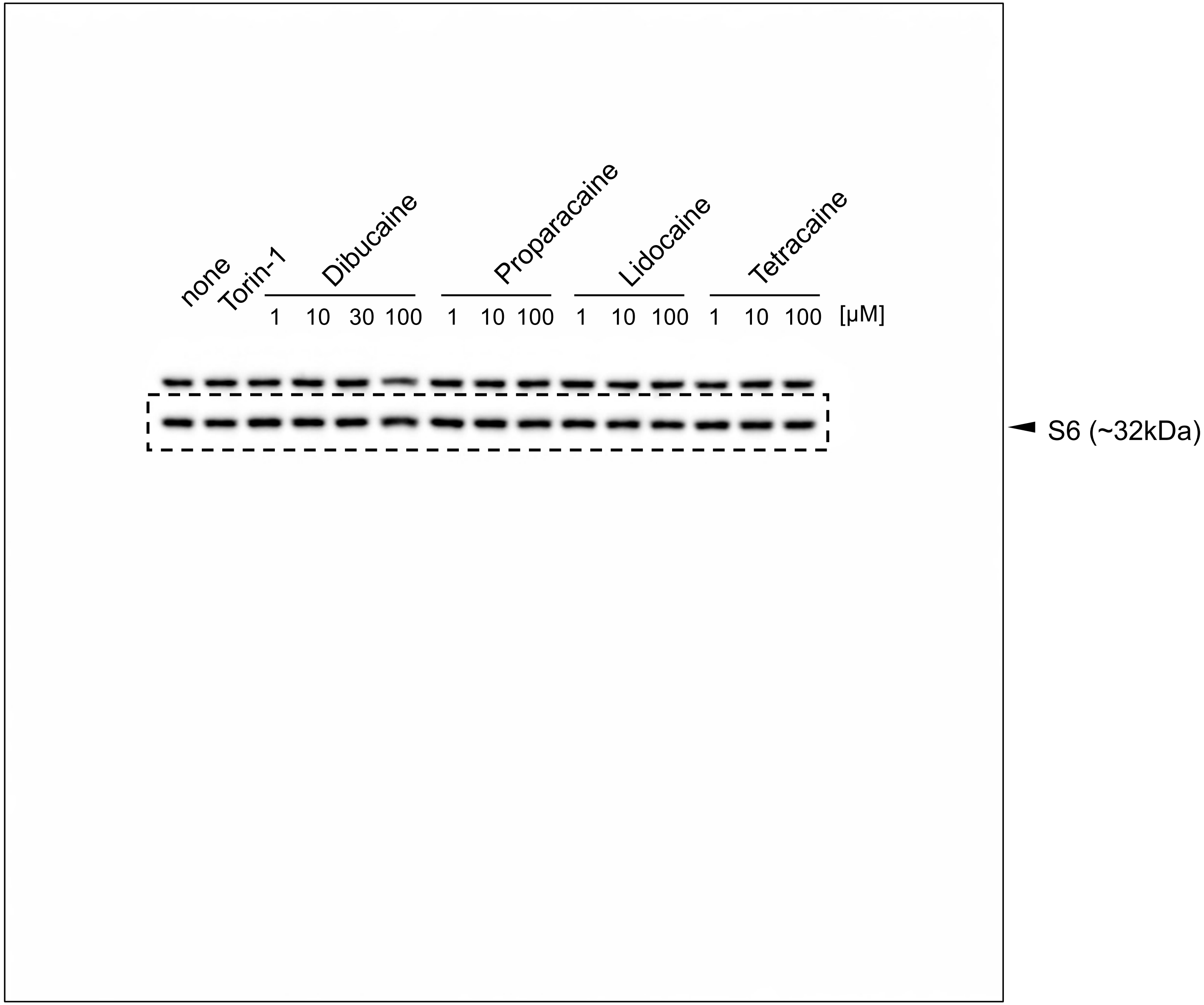

Uncropped original membrane image for Supplementary Figure S5B. A molecular-weight marker lane was not captured or retained in the available image file. The expected molecular weight of S6 is approximately 32 kDa. The expected molecular weight is provided for reference and does not represent a molecular-weight marker measured in this image.

Western Blot Source Data

for Supplementary Figure S5B

p-ULK1 (S757)

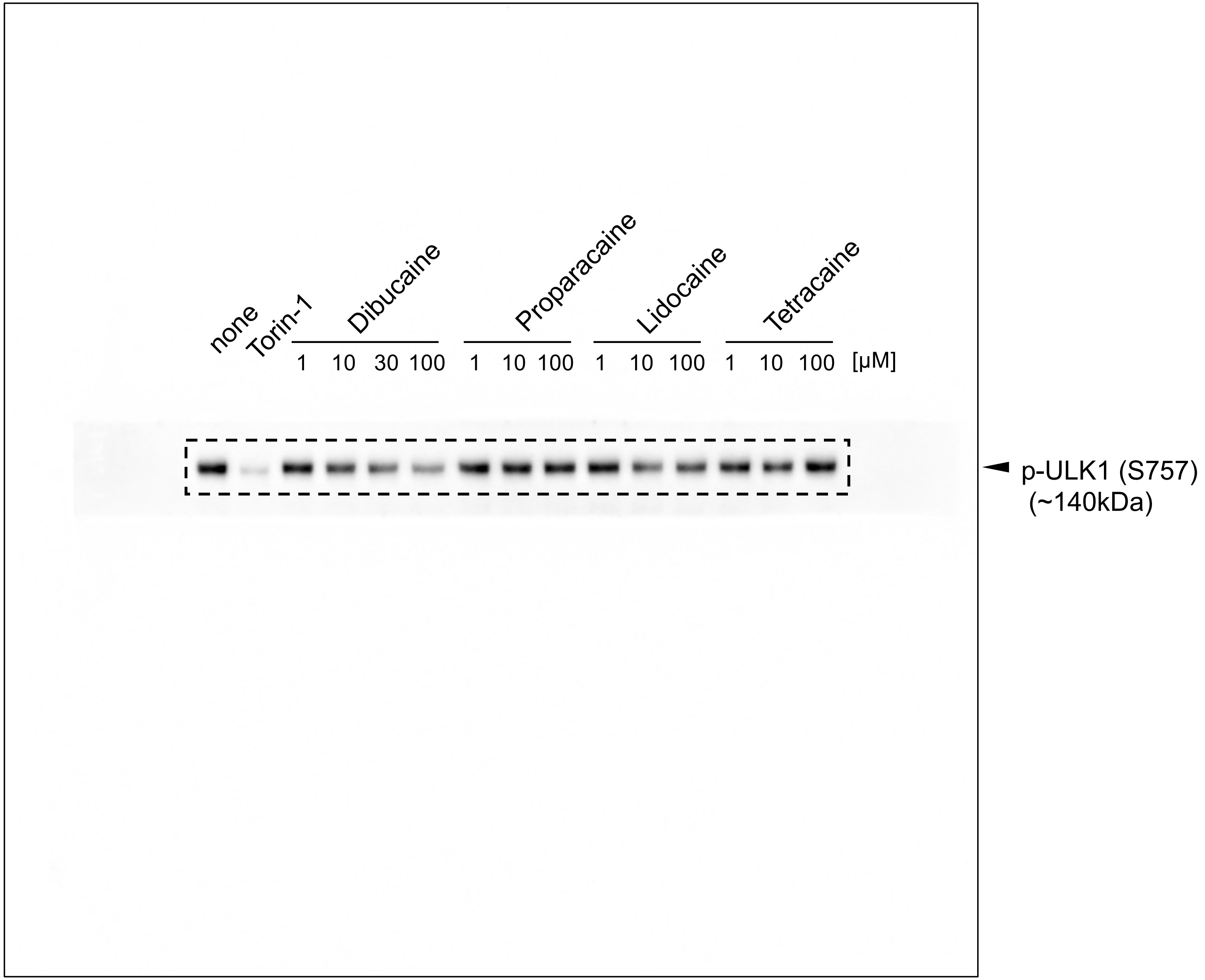

Uncropped original membrane image for Supplementary Figure S5B. A molecular-weight marker lane was not captured or retained in the available image file. The expected molecular weight of p-ULK1(S757) is approximately 140 kDa. The expected molecular weight is provided for reference and does not represent a molecular-weight marker measured in this image.

Western Blot Source Data

for Supplementary Figure S5B

ULK1

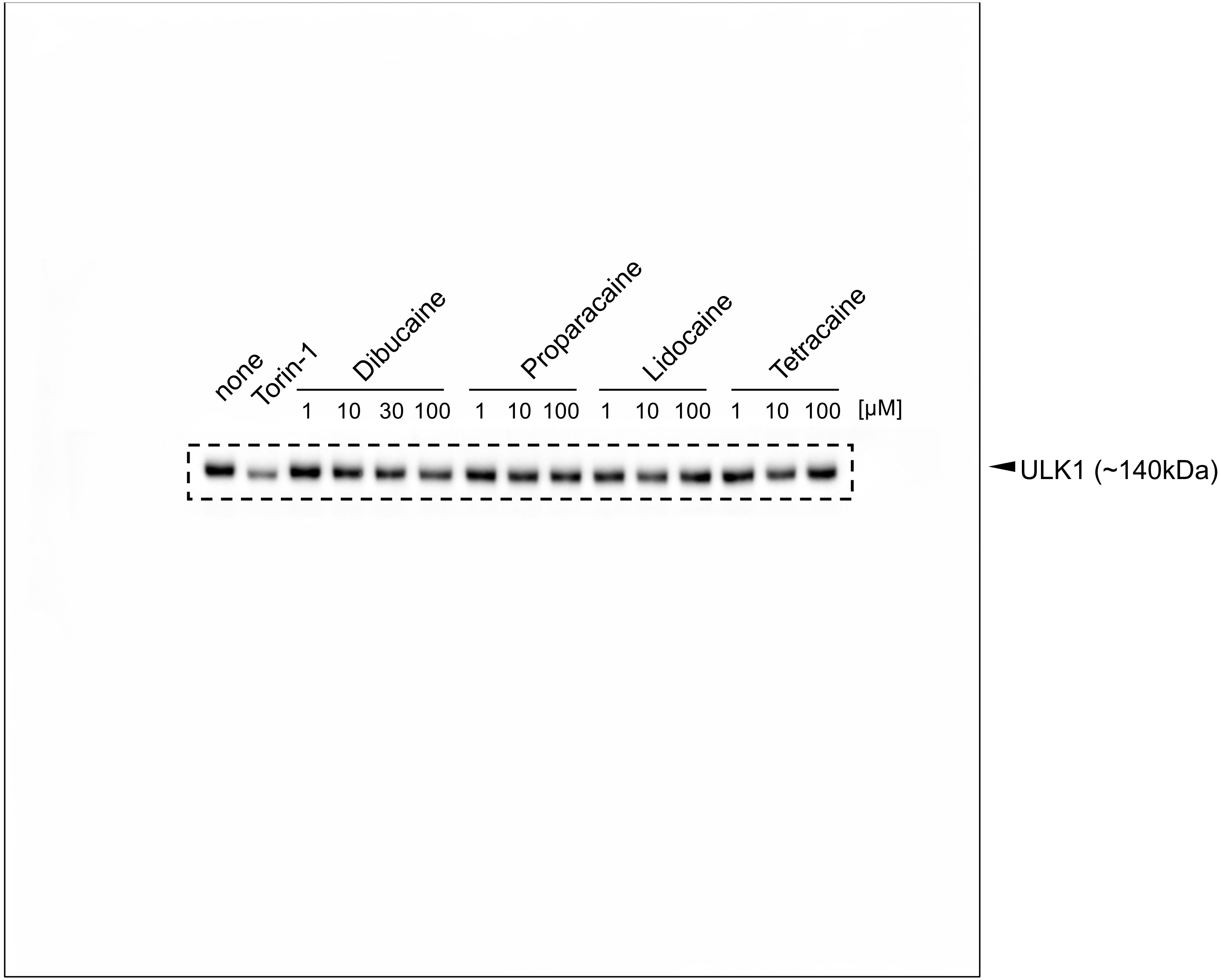

Uncropped original membrane image for Supplementary Figure S5B. A molecular-weight marker lane was not captured or retained in the available image file. The expected molecular weight of ULK1 is approximately 140 kDa. The expected molecular weight is provided for reference and does not represent a molecular-weight marker measured in this image.

Western Blot Source Data

for Supplementary Figure S5B

GAPDH

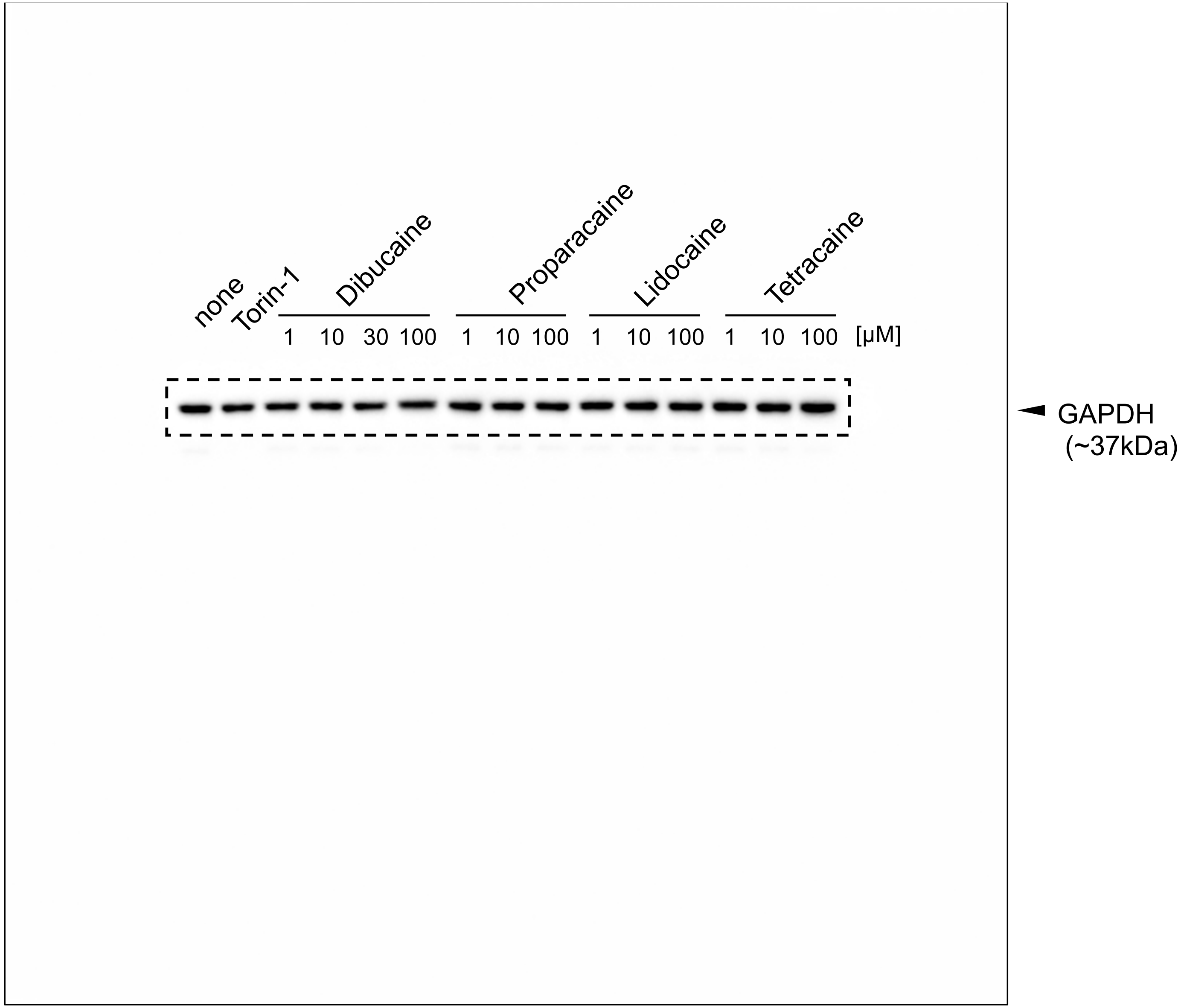

Uncropped original membrane image for Supplementary Figure S5B. A molecular-weight marker lane was not captured or retained in the available image file. The expected molecular weight of GAPDH is approximately 37 kDa. The expected molecular weight is provided for reference and does not represent a molecular-weight marker measured in this image.

Western Blot Source Data for Supplementary Figure S6

LC3,
Jak3 inhibitor VI

Uncropped source data for Supplementary Figure S6 (LC3). Left: Brightfield image of the membrane with molecular markers. Right: Corresponding chemiluminescence image. Lanes outside the dotted box contain unrelated samples. Dotted box indicates the region displayed in Supplementary Figure S6.

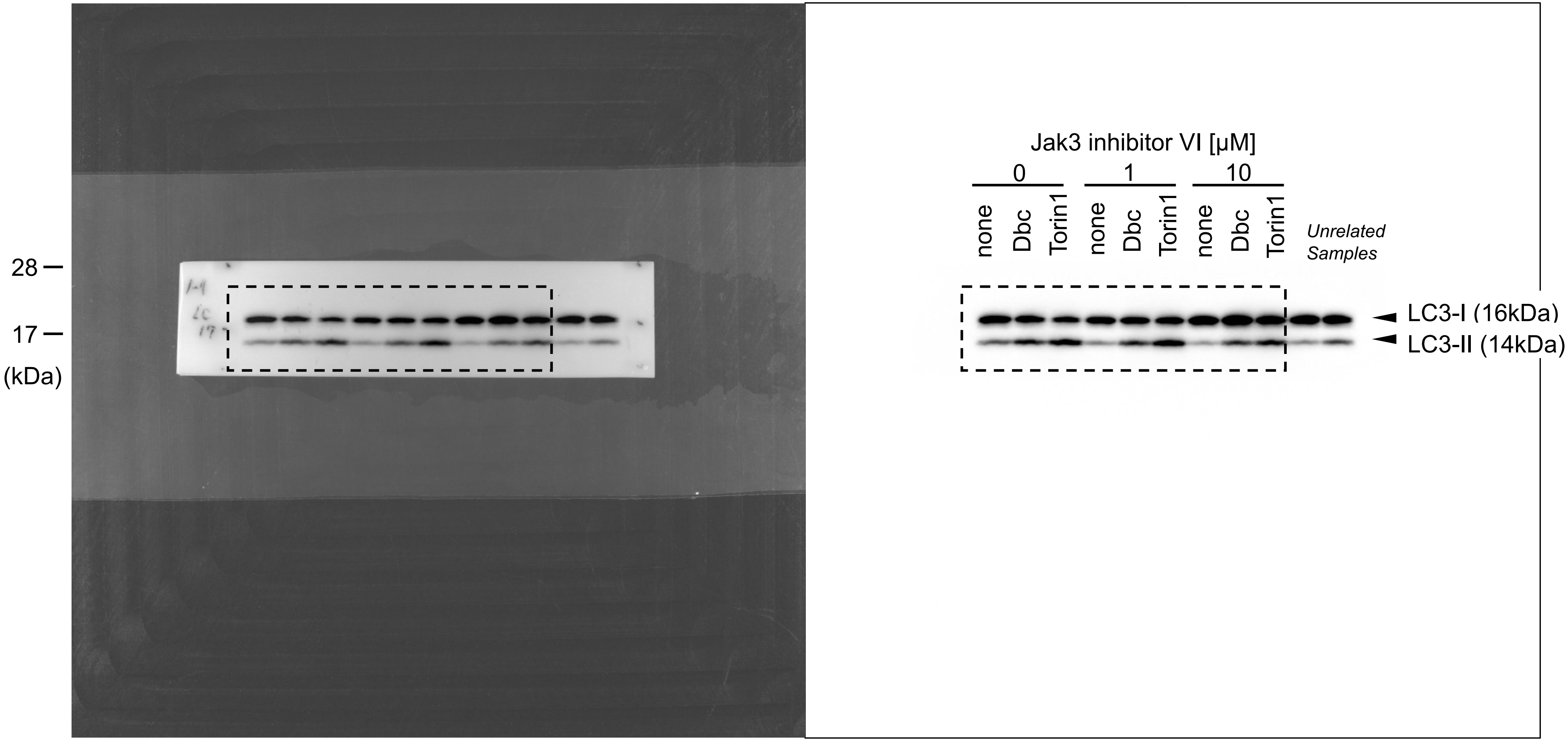

Western Blot Source Data for Supplementary Figure S6

GAPDH
Jak3 inhibitor VI

Uncropped source data for Supplementary Figure S6 (GAPDH). Left: Brightfield image of the membrane with molecular markers. Right: Corresponding chemiluminescence image. Lanes outside the dotted box contain unrelated samples. Dotted box indicates the region displayed in Supplementary Figure S6.

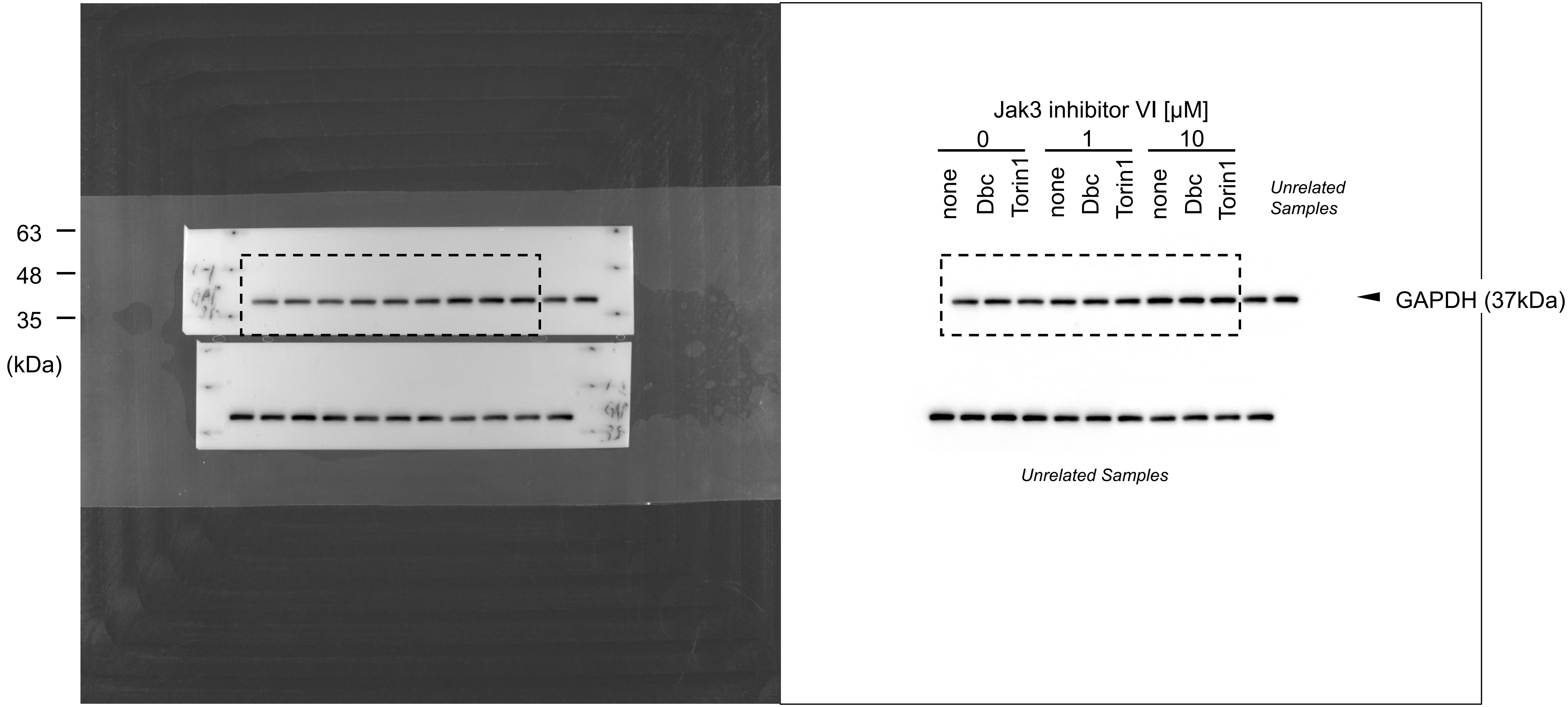

Western Blot Source Data for Supplementary Figure S6

Uncropped source data for Supplementary Figure S6 (LC3). Left: Brightfield image of the membrane with molecular markers. Right: Corresponding chemiluminescence image. Dotted boxes indicate the regions displayed in Supplementary Figure S6.

LC3,
AG1478

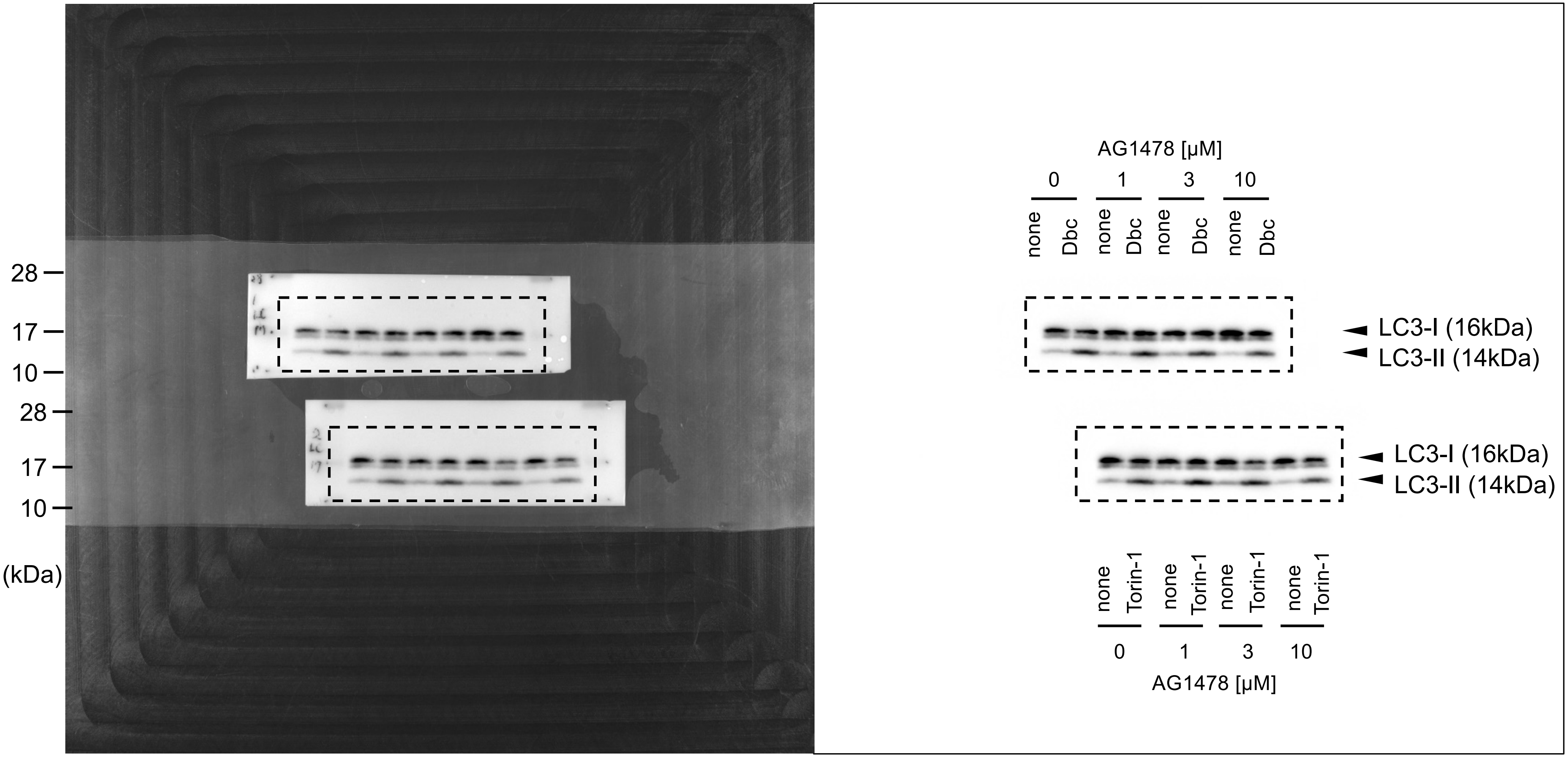

Western Blot Source Data for Supplementary Figure S6

Uncropped source data for Supplementary Figure S6 (GAPDH). Left: Brightfield image of the membrane with molecular markers. Right: Corresponding chemiluminescence image. Dotted boxes indicate the regions displayed in Supplementary Figure S6.

GAPDH,
AG1478

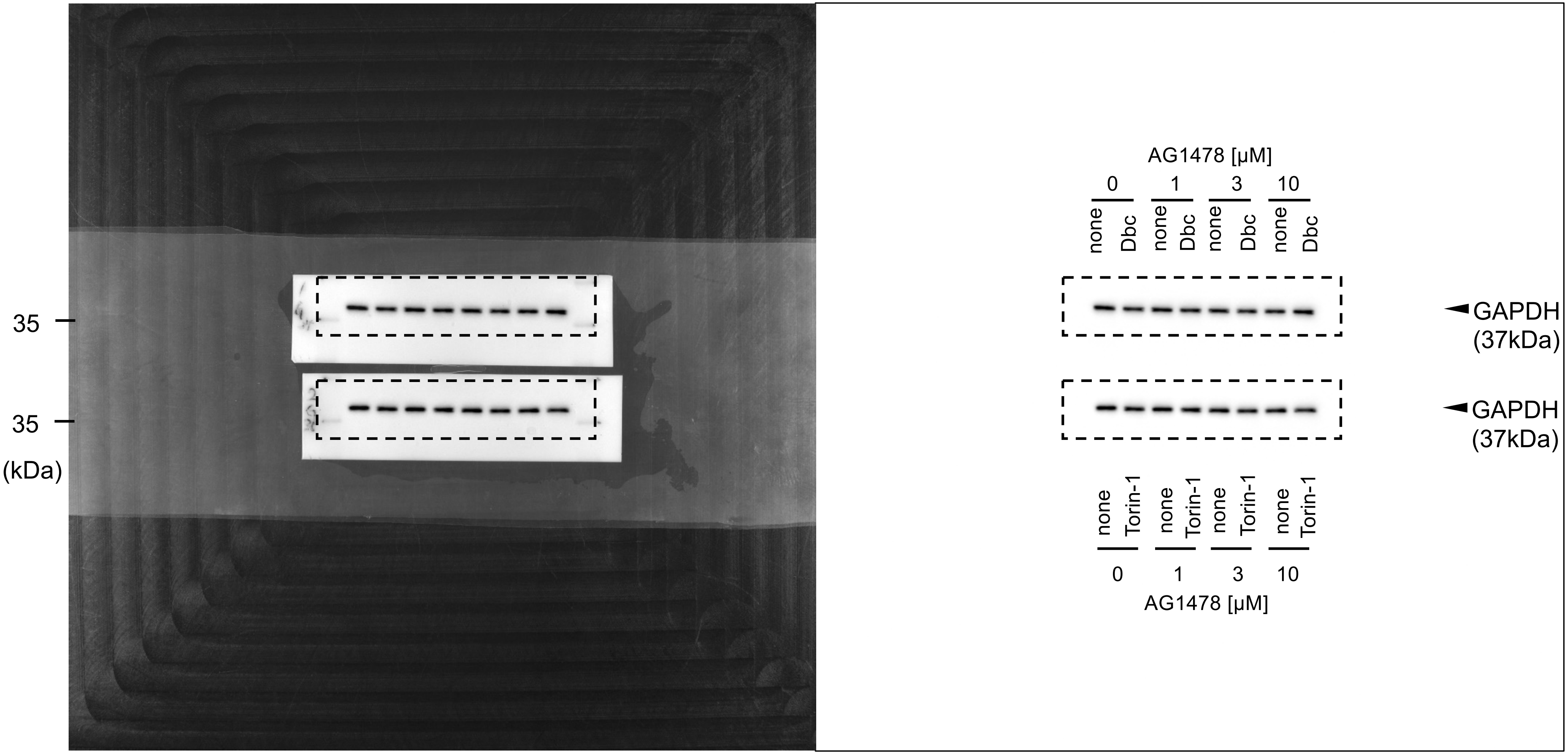

Western Blot Source Data for Supplementary Figure S6

LC3,
Sorafenib

Uncropped source data for Supplementary Figure S6 (LC3). Left: Brightfield image of the membrane with molecular markers. Right: Corresponding chemiluminescence image. Dotted boxes indicate the regions displayed in Supplementary Figure S6.

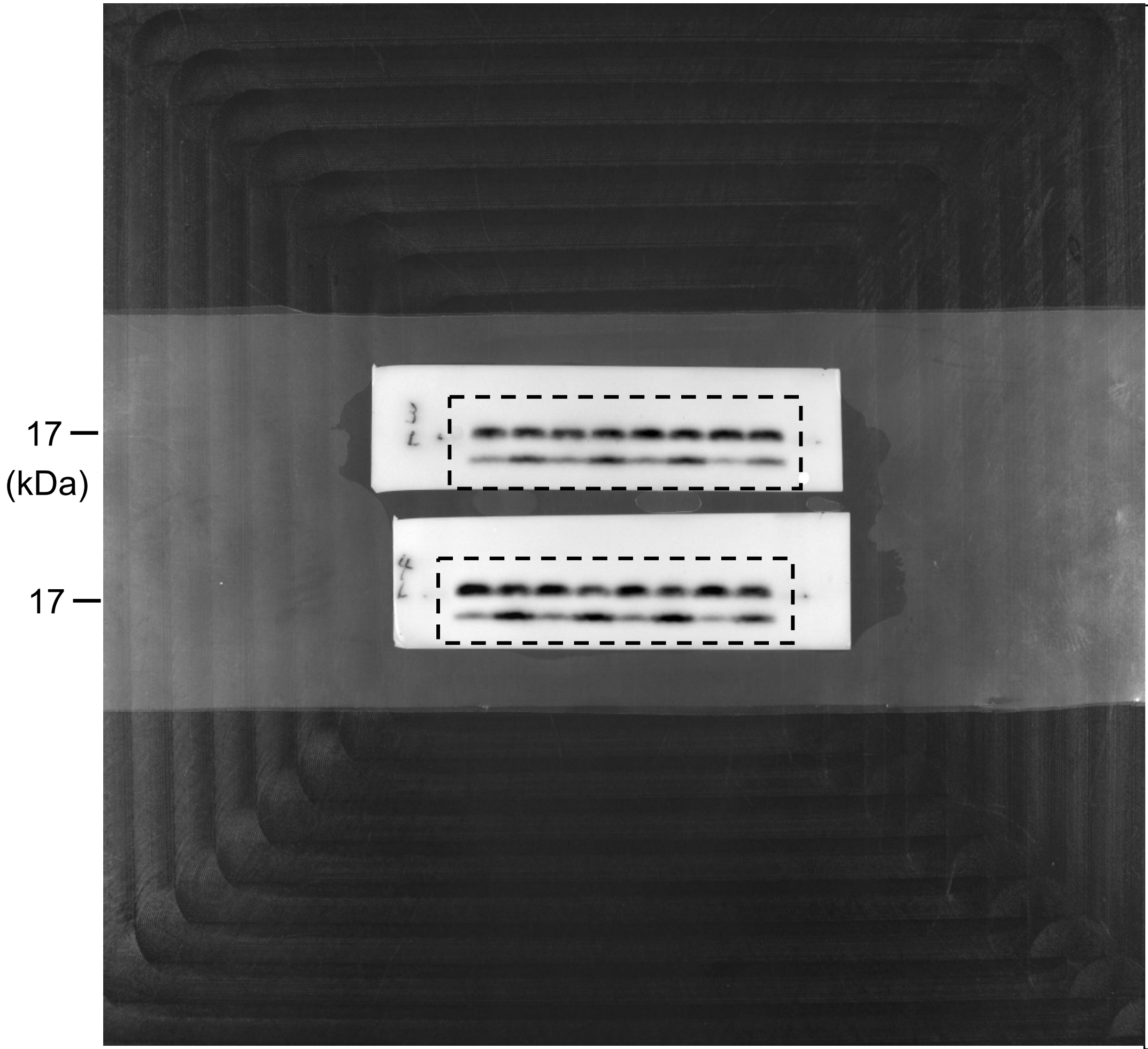

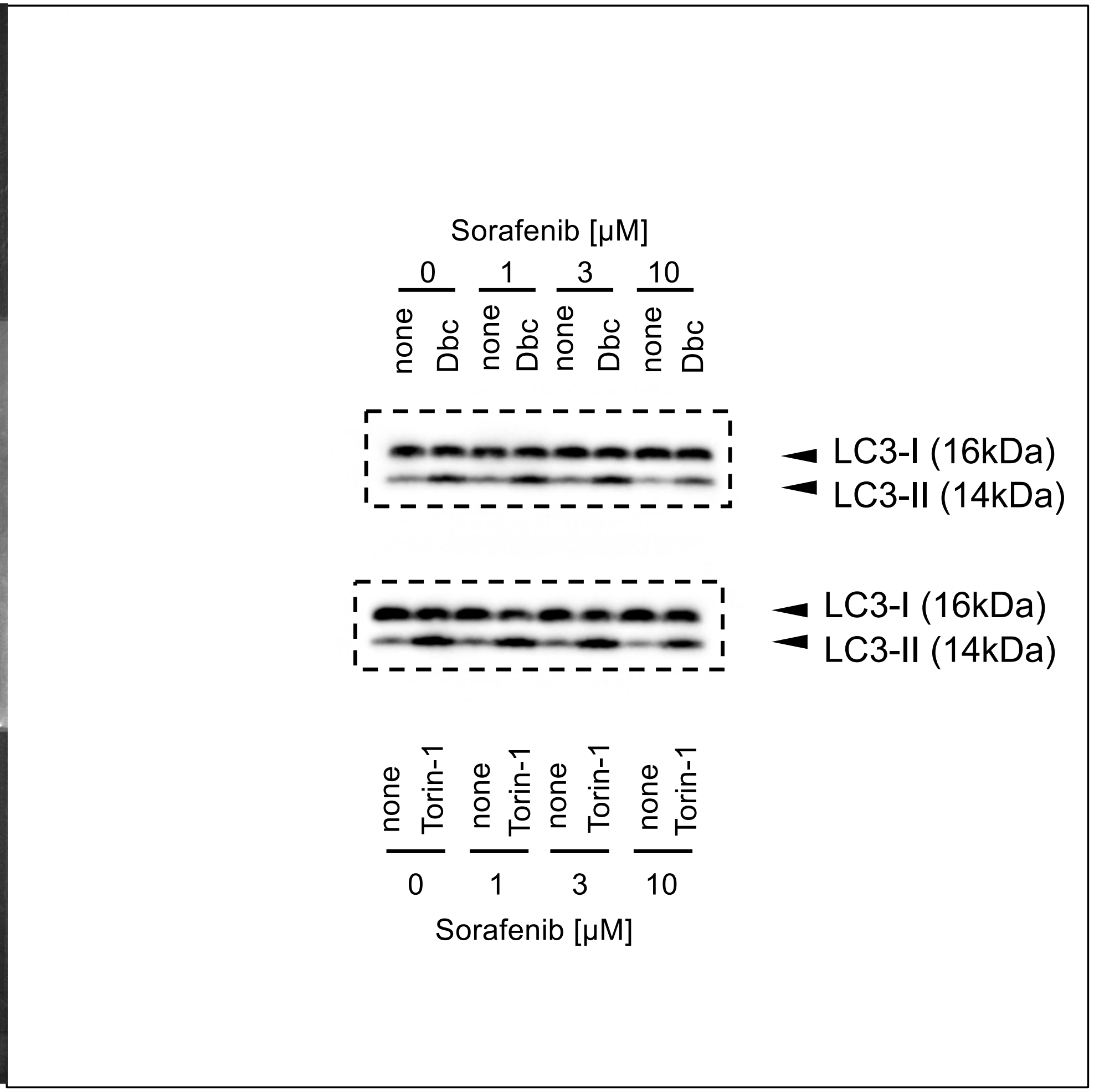

Western Blot Source Data for Supplementary Figure S6

LC3,
Sorafenib (non-saturated)

Uncropped source data for Supplementary Figure S6 (LC3, non-saturated exposure). Left: Brightfield image of the membrane with molecular markers. Right: Corresponding chemiluminescence image (non-saturated). Dotted box indicates the region displayed in Supplementary Figure S6.

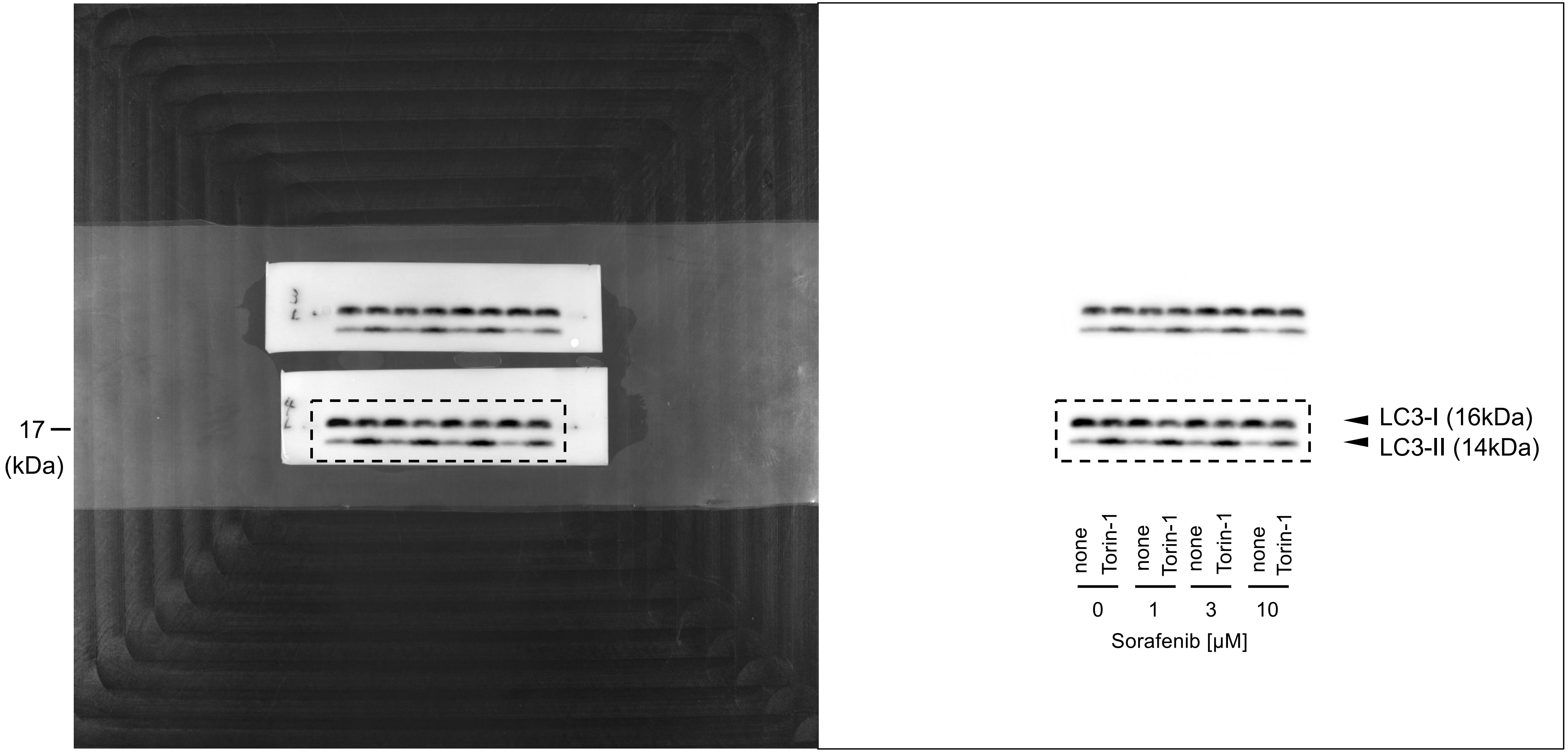

Western Blot Source Data for Supplementary Figure S6

GAPDH,
Sorafenib

Uncropped source data for Supplementary Figure S6 (GAPDH). Left: Brightfield image of the membrane with molecular markers. Right: Corresponding chemiluminescence image. Dotted boxes indicate the regions displayed in Supplementary Figure S6.

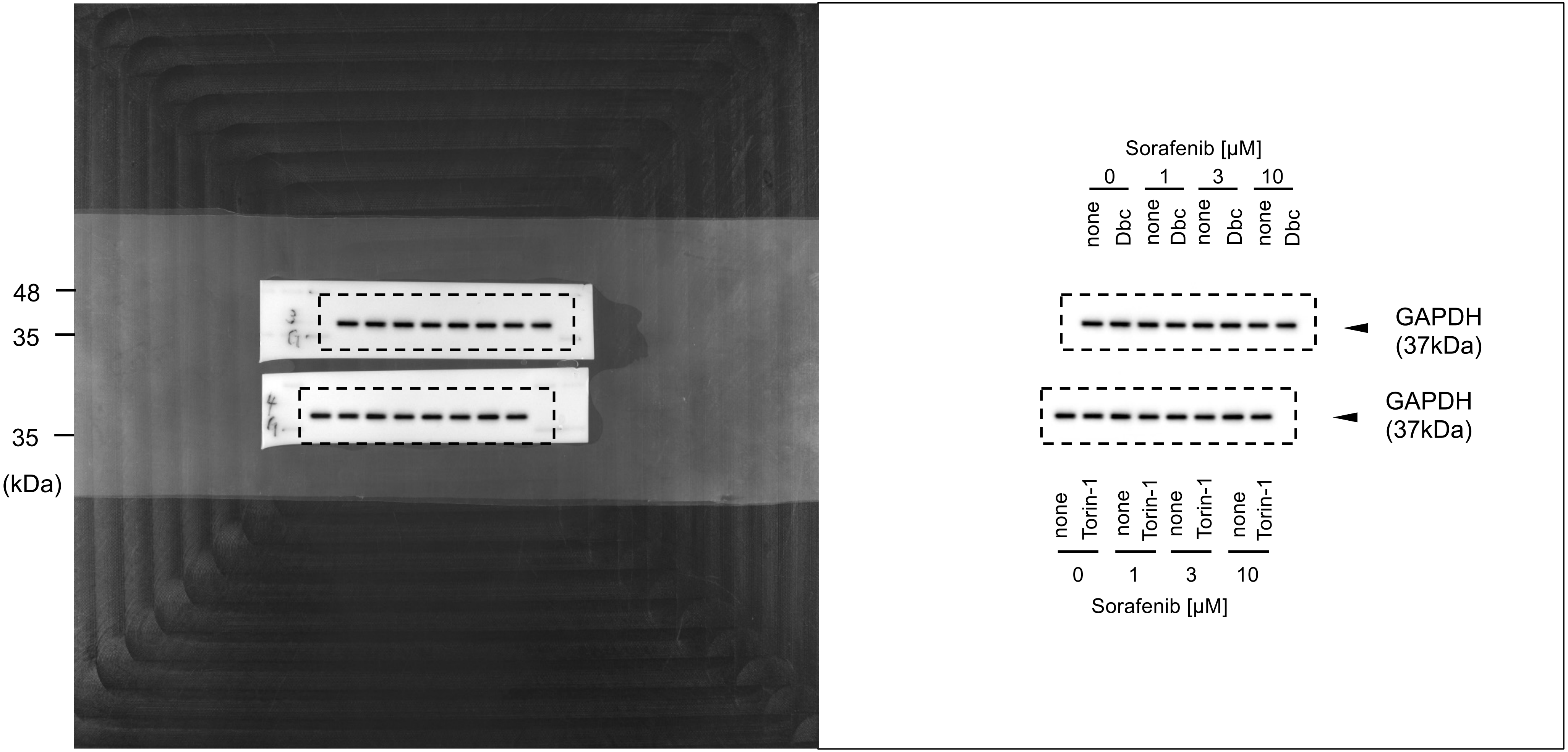

Western Blot Source Data for Supplementary Figure S7

ALK5,
siALK5

Uncropped source data for Supplementary Figure S7 (ALK5). Left: Brightfield image of the membrane with molecular markers. Right: Corresponding chemiluminescence image. Dotted box indicates the region displayed in Supplementary Figure S7.

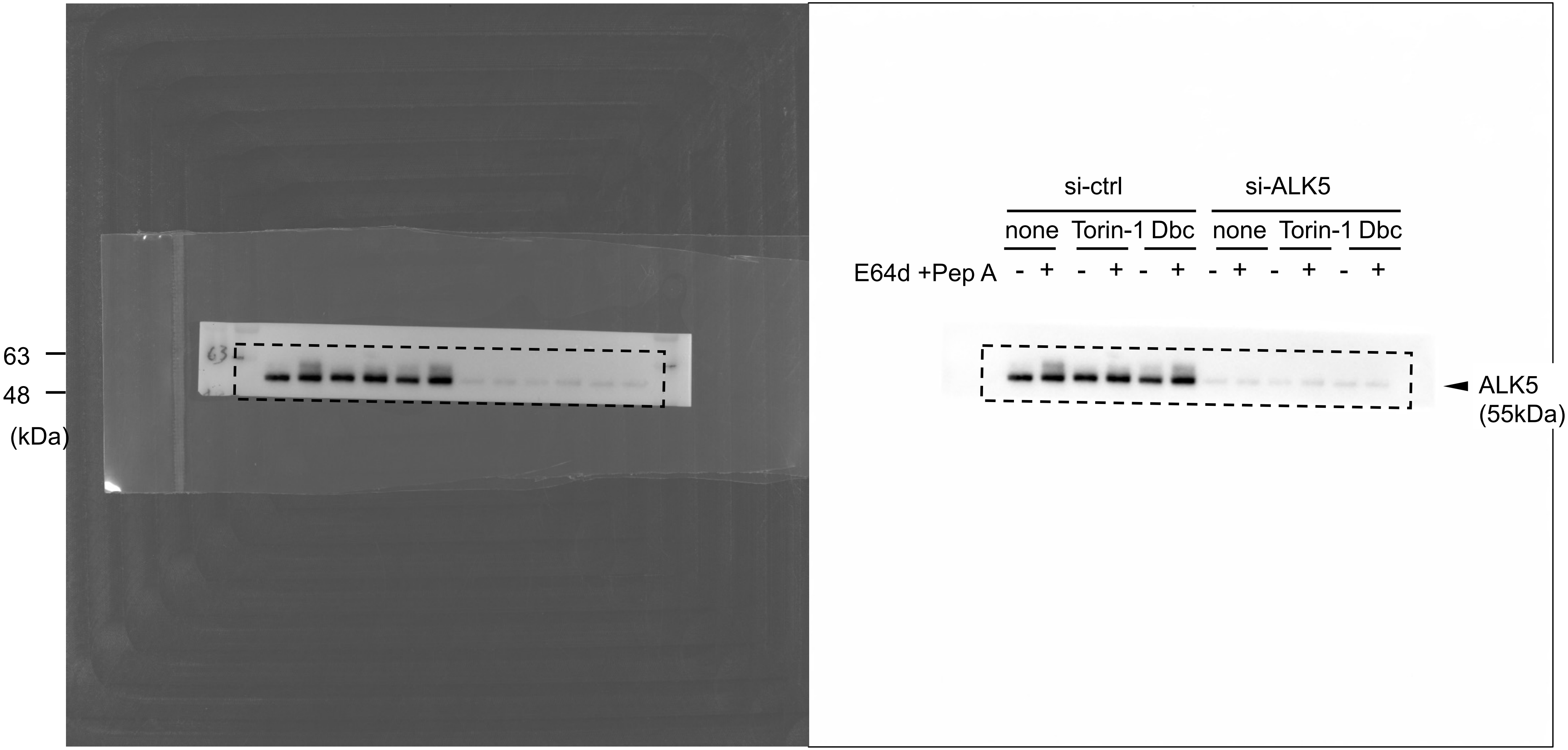

Western Blot Source Data for Supplementary Figure S7

LC3,
siALK5

Uncropped source data for Supplementary Figure S7 (LC3). Left: Brightfield image of the membrane with molecular markers. Right: Corresponding chemiluminescence image. Dotted box indicates the region displayed in Supplementary Figure S7.

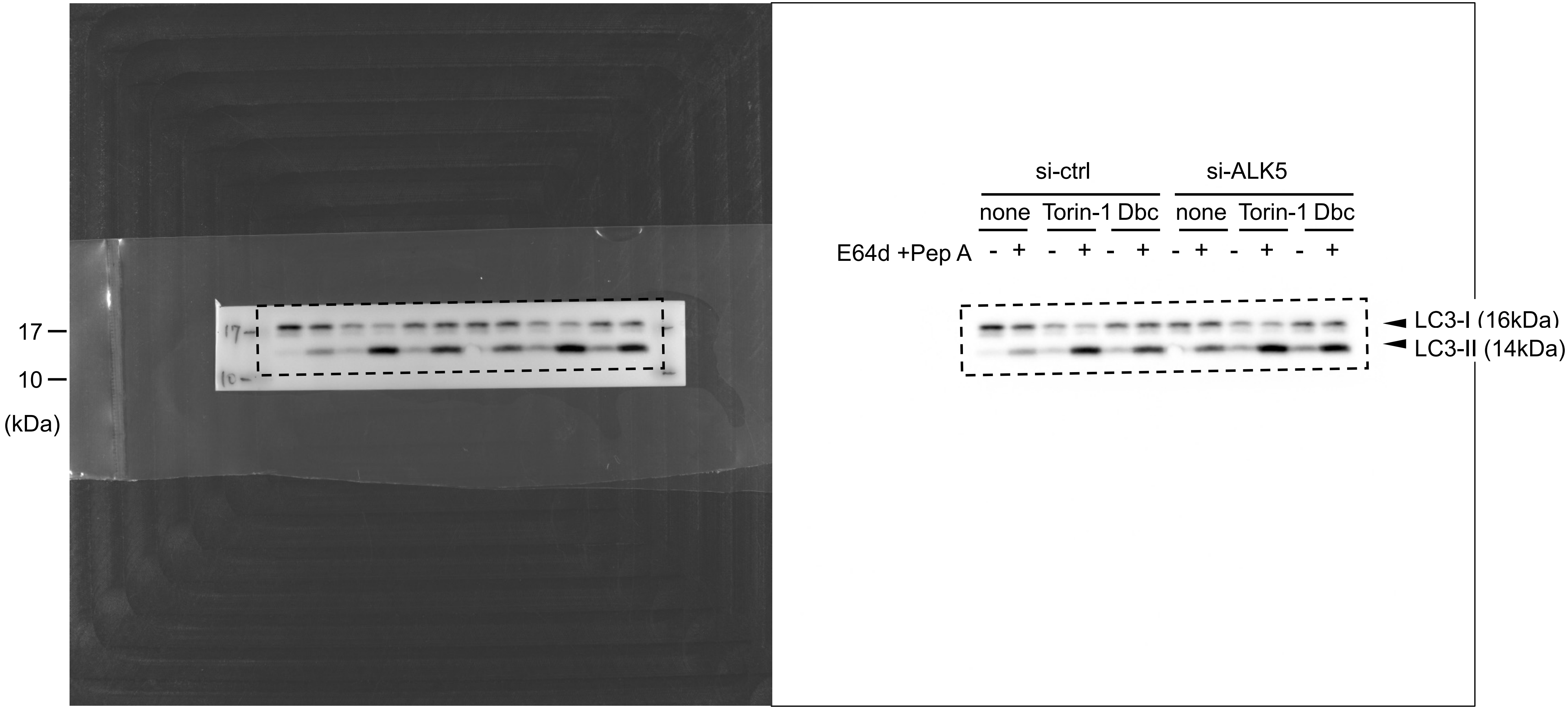

Western Blot Source Data for Supplementary Figure S7

GAPDH,
siALK5

Uncropped source data for Supplementary Figure S7 (GAPDH). Left: Brightfield image of the membrane with molecular markers. Right: Corresponding chemiluminescence image. Dotted box indicates the region displayed in Supplementary Figure S7.

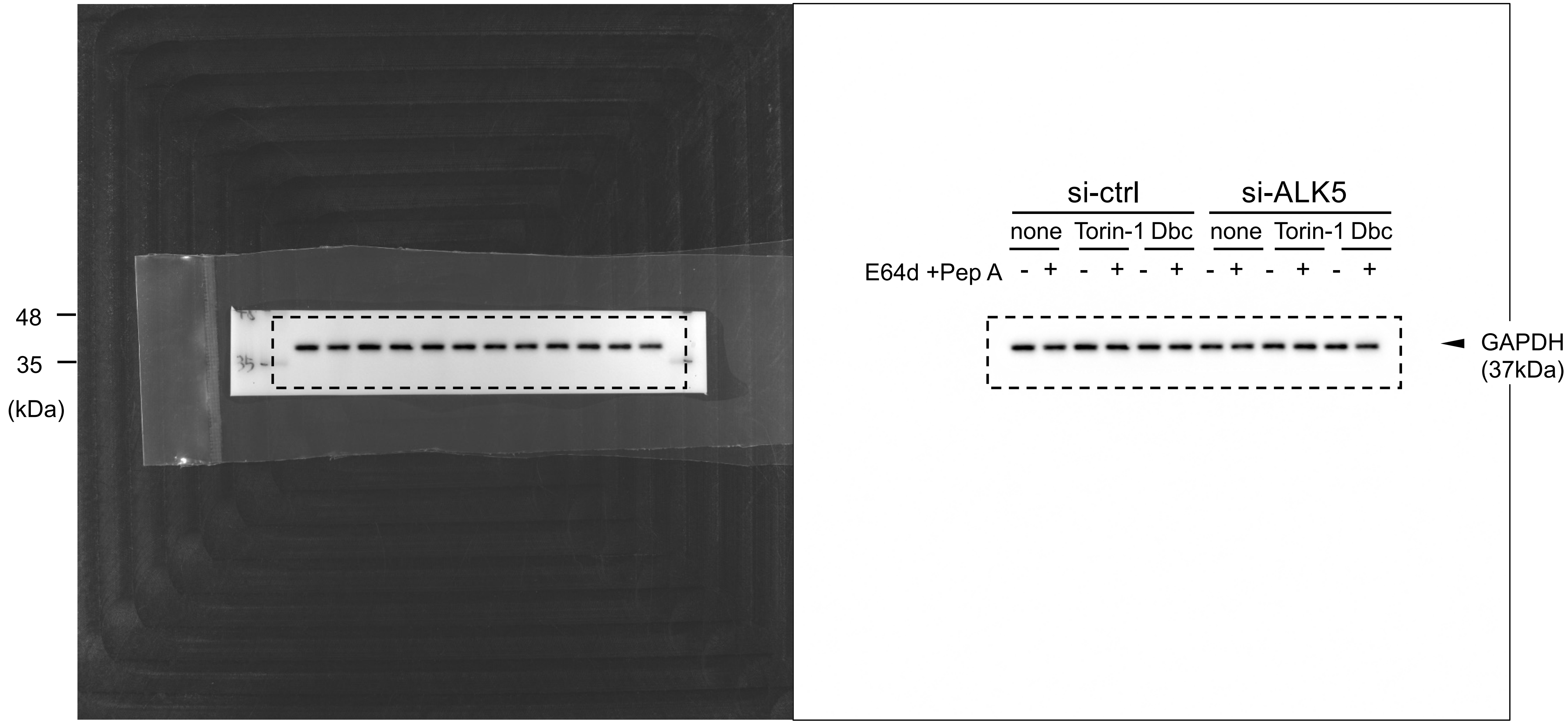

Western Blot Source Data for Supplementary Figure S7

RIPK2,
siRIPK2

Uncropped source data for Supplementary Figure S7 (RIPK2). Left: Brightfield image of the membrane with molecular markers. Right: Corresponding chemiluminescence image. Dotted box indicates the region displayed in Supplementary Figure S7.

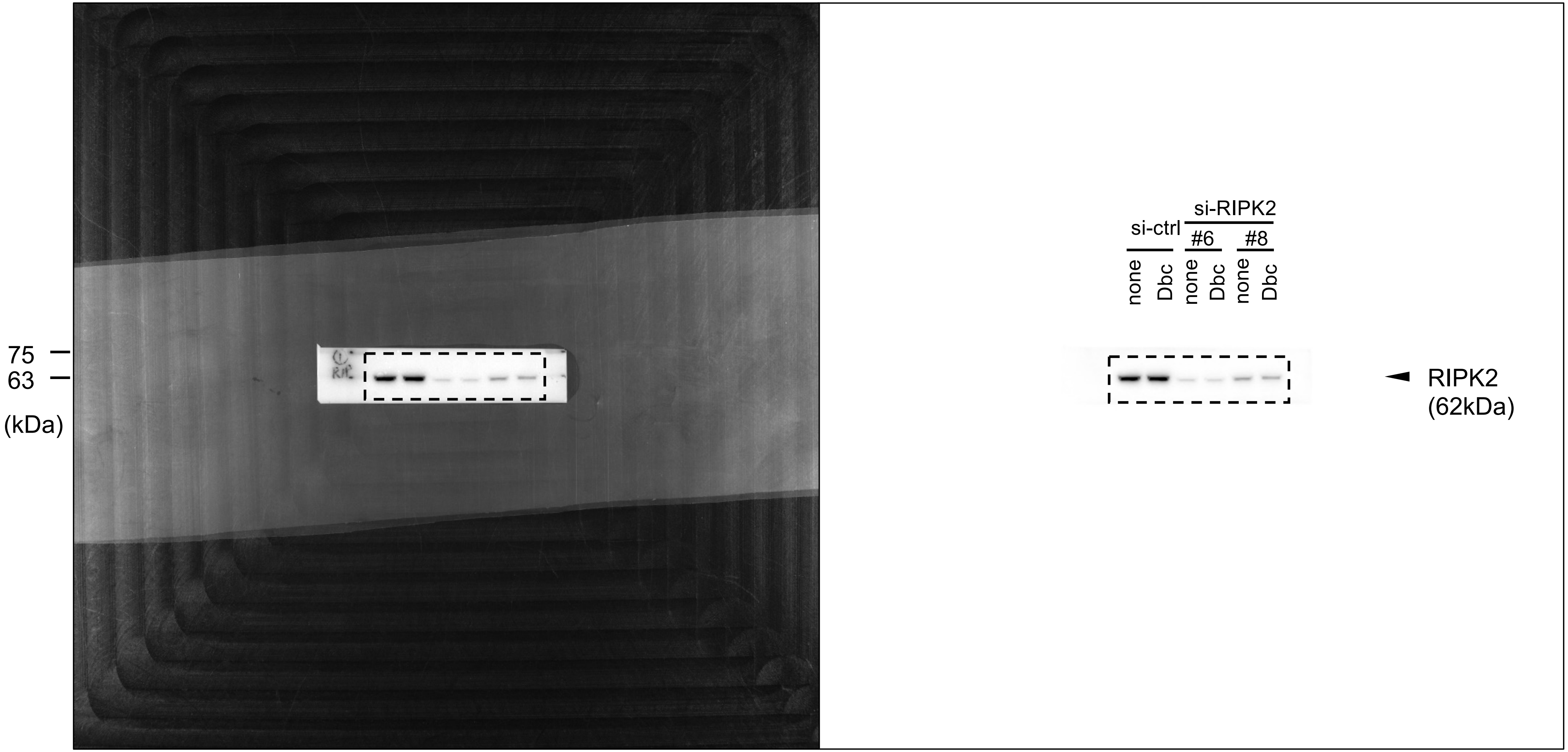

Western Blot Source Data for Supplementary Figure S7

LC3,
siRIPK2

Uncropped source data for Supplementary Figure S7 (LC3). Left: Brightfield image of the membrane with molecular markers. Right: Corresponding chemiluminescence image. Dotted box indicates the region displayed in Supplementary Figure S7.

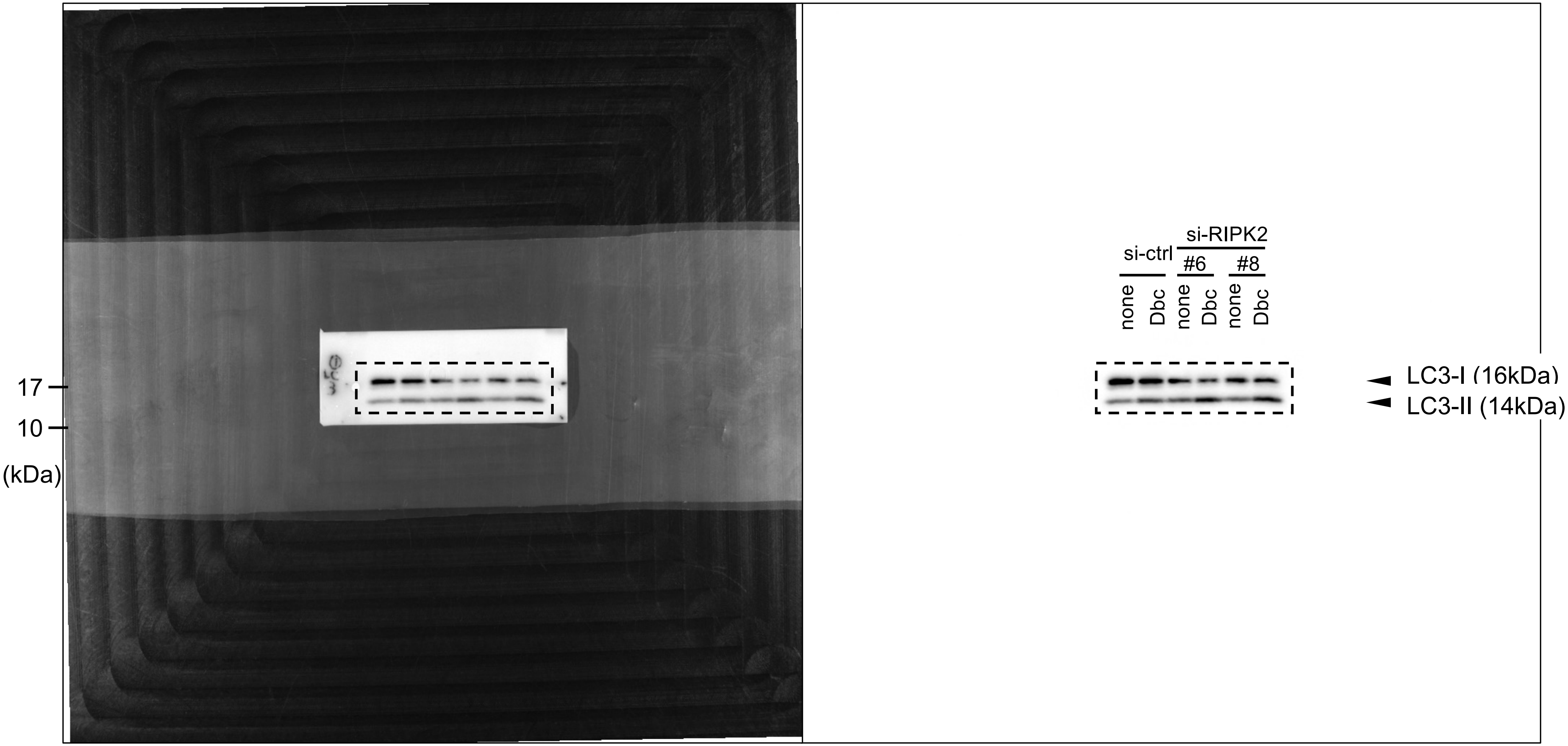

Western Blot Source Data for Supplementary Figure S7

GAPDH,
siRIPK2

Uncropped source data for Supplementary Figure S7 (GAPDH). Left: Brightfield image of the membrane with molecular markers. Right: Corresponding chemiluminescence image. Dotted box indicates the region displayed in Supplementary Figure S7.

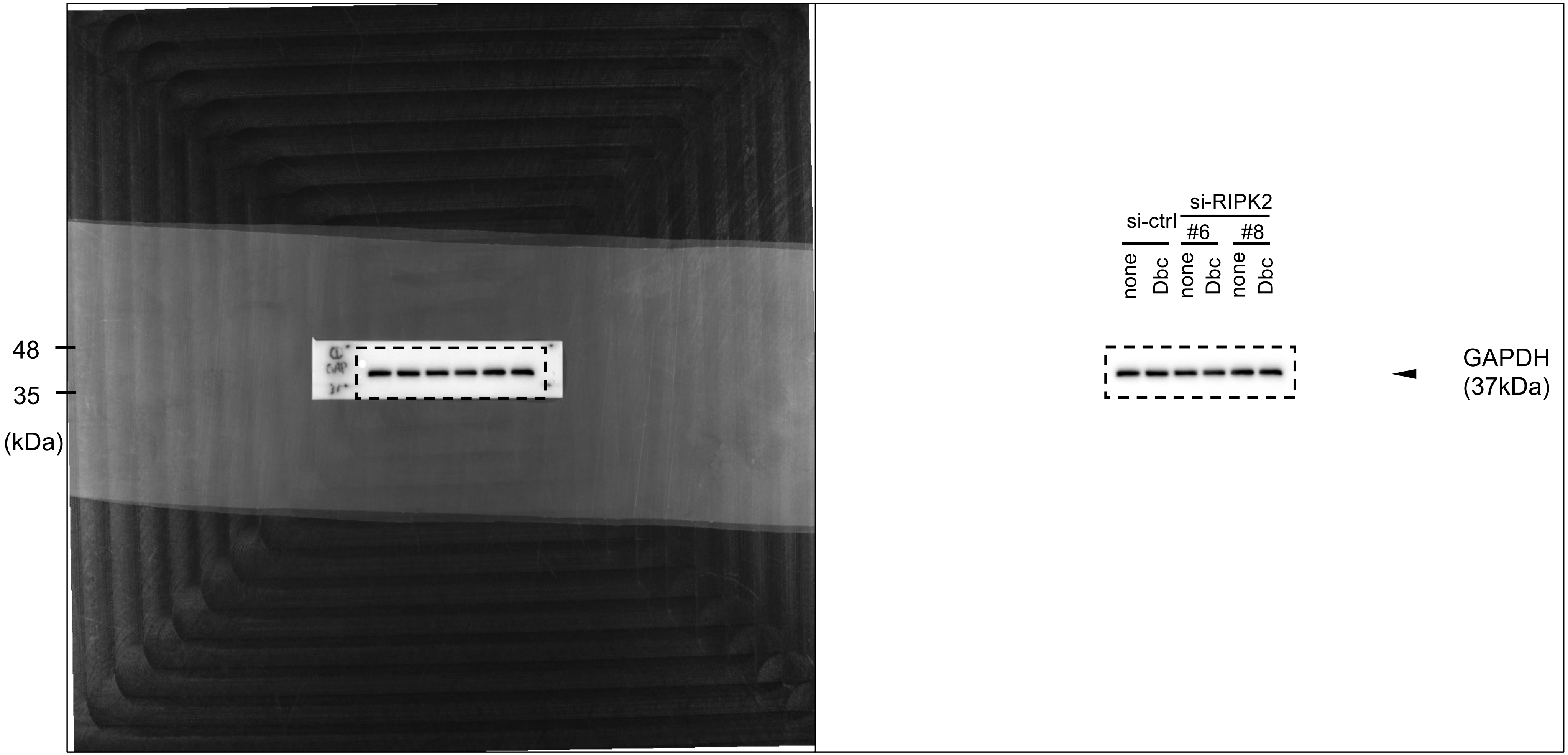

Western Blot Source Data
for Supplementary Figure S7

CK1δ,
siCK1δ

Uncropped source data for Supplementary Figure S7 (CK1δ). Left: Brightfield image of the membrane with molecular markers. Right: Corresponding chemiluminescence image. Dotted box indicates the region displayed in Supplementary Figure S7.

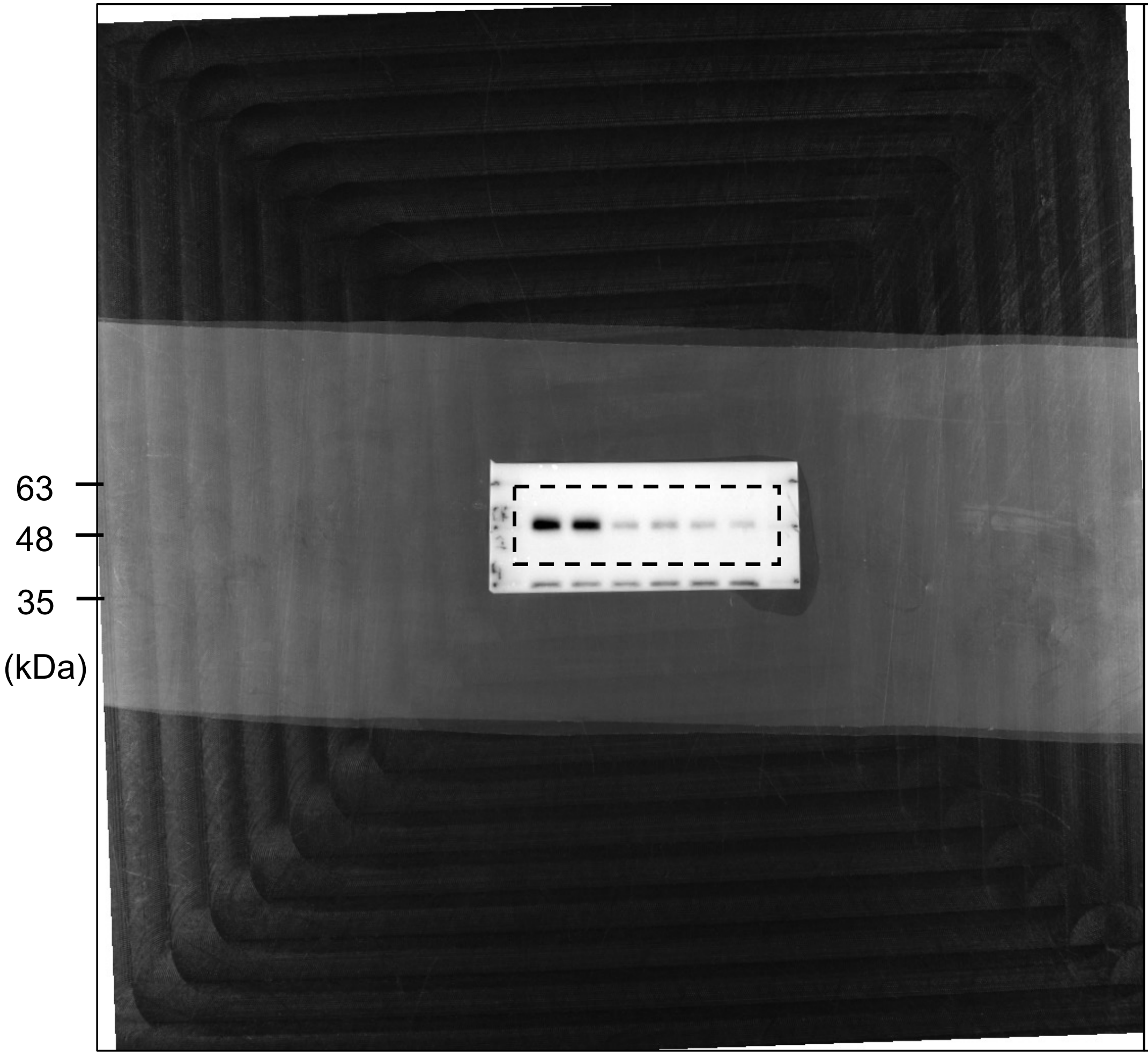

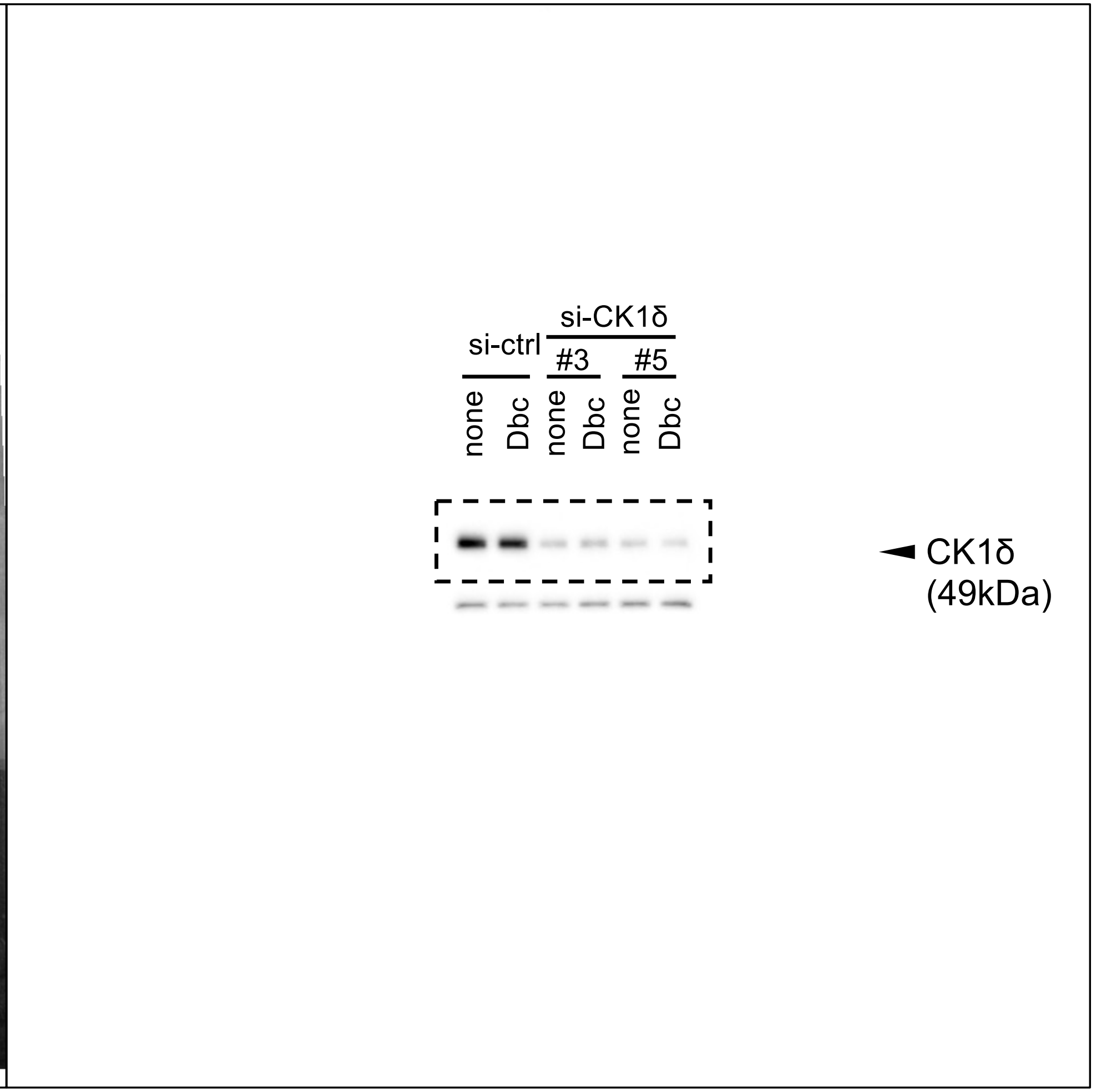

Western Blot Source Data for Supplementary Figure S7

LC3,
siCK1δ

Uncropped source data for Supplementary Figure S7 (LC3). Left: Brightfield image of the membrane with molecular markers. Right: Corresponding chemiluminescence image. Dotted box indicates the region displayed in Supplementary Figure S7.

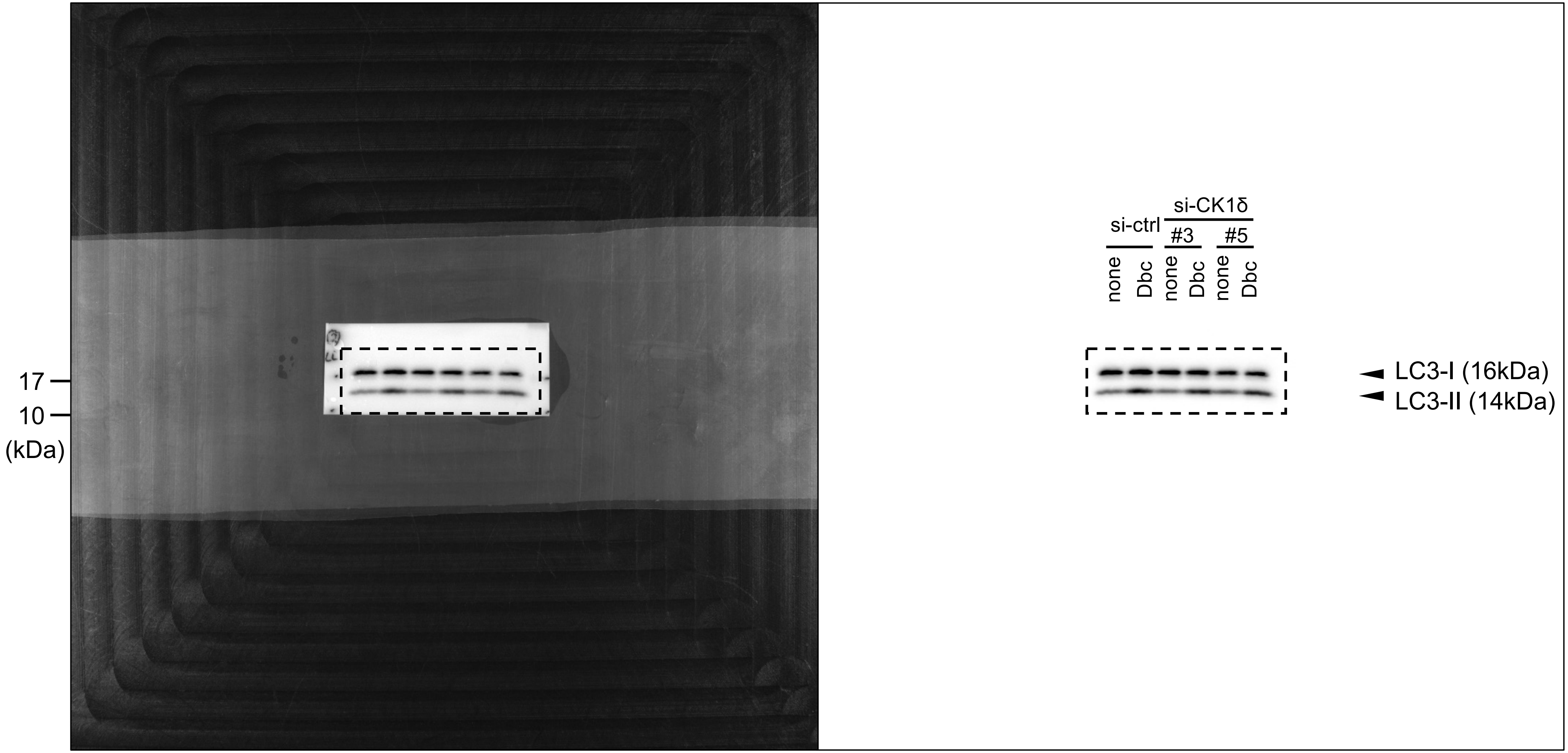

Western Blot Source Data for Supplementary Figure S7

GAPDH,
siCK1δ

Uncropped source data for Supplementary Figure S7 (GAPDH). Left: Brightfield image of the membrane with molecular markers. Right: Corresponding chemiluminescence image. Dotted box indicates the region displayed in Supplementary Figure S7.

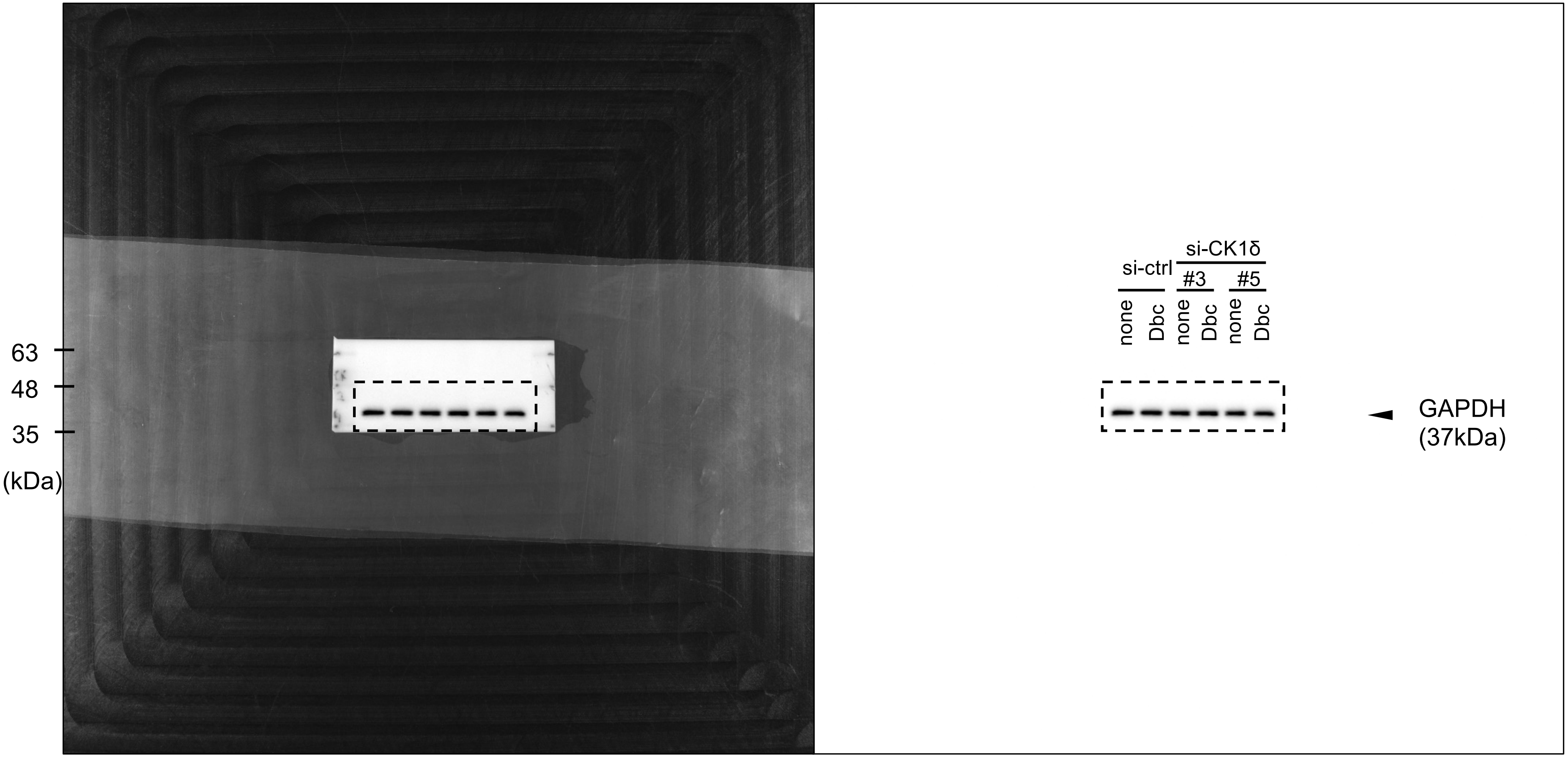

Supplement: Supplementary file 1 [file Data_Sheet_1.pdf]
